# Highly Selective SERCA2a Activators: Preclinical Development of a Congeneric Group of First-in-Class Drug Leads against Heart Failure

**DOI:** 10.1021/acs.jmedchem.2c00347

**Published:** 2022-05-17

**Authors:** Andrea Luraghi, Mara Ferrandi, Paolo Barassi, Martina Arici, Shih-Che Hsu, Eleonora Torre, Carlotta Ronchi, Alessio Romerio, Gwo-Jyh Chang, Patrizia Ferrari, Giuseppe Bianchi, Antonio Zaza, Marcella Rocchetti, Francesco Peri

**Affiliations:** † Department of Biotechnology and Biosciences, University of Milano-Bicocca, Milano 20126, Italy; ‡ Windtree Therapeutics Inc., Warrington, Pennsylvania 18976, United States; § CVie Therapeutics Limited, Taipei 11047 Taiwan; ∥ Cardiovascular Medicine, Chang Gung University, Tao-Yuan 333323 Taiwan; ⊥ Università Vita-Salute San Raffaele, Milano 20132, Italy

## Abstract

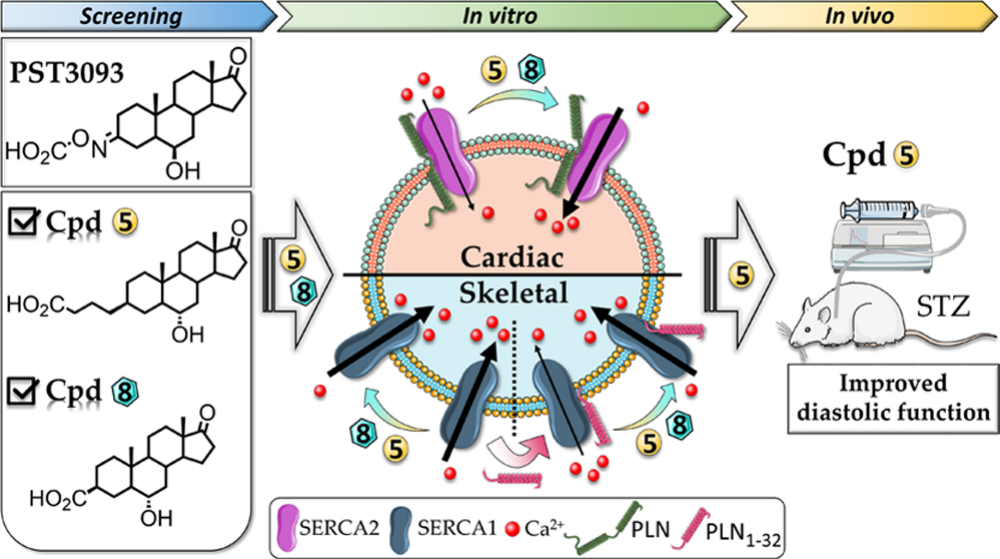

The stimulation of sarcoplasmic reticulum calcium ATPase SERCA2a emerged as a novel therapeutic strategy to efficiently improve overall cardiac function in heart failure (HF) with reduced arrhythmogenic risk. Istaroxime is a clinical-phase IIb compound with a double mechanism of action, Na^+^/K^+^ ATPase inhibition and SERCA2a stimulation. Starting from the observation that istaroxime metabolite PST3093 does not inhibit Na^+^/K^+^ ATPase while stimulates SERCA2a, we synthesized a series of bioisosteric PST3093 analogues devoid of Na^+^/K^+^ ATPase inhibitory activity. Most of them retained SERCA2a stimulatory action with nanomolar potency in cardiac preparations from healthy guinea pigs and streptozotocin (STZ)-treated rats. One compound was further characterized in isolated cardiomyocytes, confirming SERCA2a stimulation and in vivo showing a safety profile and improvement of cardiac performance following acute infusion in STZ rats. We identified a new class of selective SERCA2a activators as first-in-class drug candidates for HF treatment.

## Introduction

Heart failure (HF) is a life-threatening syndrome characterized by an inability of the heart to meet the metabolic demands of the body. It is age-dependent, ranging from less than 2% of people younger than 60 years to more than 10% of individuals older than 75 years. Most patients with HF have a history of hypertension, coronary artery disease, cardiomyopathies, valve disease, or a combination of these disorders.^1^ The calculated lifetime risk of developing HF is expected to increase, and those with hypertension are at higher risk.^2^ Clinical symptoms in HF are caused by a cardiac double pathological feature that consists of diminished systolic emptying (systolic dysfunction) and impaired ability of the ventricles to receive blood from the venous system (diastolic dysfunction). The impaired contractility and relaxation are also caused by an abnormal distribution of intracellular Ca^2+^, resulting from reduced Ca^2+^ uptake by the sarcoplasmic reticulum (SR).^3^ SR Ca^2+^ uptake is operated by the SR Ca^2+^ ATPase, SERCA2a, a 110 kDa membrane protein. During ion transport across the membrane, SERCA2a undergoes large conformational changes switching between Ca^2+^-bound E1 and Ca^2+^-free E2 states.^4^ SERCA2a activity is physiologically inhibited by the interaction with phospholamban (PLN), a small protein^5,6^ that stabilizes the E2 state, incompatible with Ca^2+^ binding.^7^ SERCA2a inhibition by PLN is normally relieved by PLN phosphorylation with protein kinase A, a signaling pathway severely depressed as a consequence of myocardial remodeling.^8^ SERCA2a activators are therefore promising drugs that might improve overall cardiac function in HF with reduced arrhythmogenic risk. Various therapeutic approaches that increase SERCA2a function have been recently investigated.^9−13^ Small-molecule SERCA2a activators have been recently discovered. Among them, a pyridone derivative directly binds to PLN displacing it from SERCA2a^14^ and a small molecule activates SERCA2a by promoting its SUMOylation [small ubiquitin-related modifier (SUMO)].^15^ Overall, new SERCA2a activators might be very useful in HF treatment together with the first line of therapeutic agents, β-blockers, and angiotensin-converting enzyme (ACE) inhibitors.

The work of our laboratory led to the successful completion of phase IIb clinical trials of the steroid derivative istaroxime,^16^ which is endowed with a double mechanism of action, that is, Na^+^/K^+^ ATPase inhibition^17^ and SERCA2a activation.^18^ Istaroxime is an inotropic/lusitropic agent, which is capable of improving both systolic and diastolic functions (HORIZON study)^19^ with a much lower proarrhythmic effect than digoxin, a pure Na^+^/K^+^ ATPase inhibitor.^18^ This suggests that, by improving Ca^2+^ clearance from the cytosol,^20^ SERCA2a stimulation may minimize the proarrhythmic effect of Na^+^/K^+^ ATPase blockade^18,19,21^ while preserving its inotropic effect. Although having an excellent pharmacodynamic profile, istaroxime is not optimal for chronic administration because of its poor gastrointestinal absorption, high clearance rate, and extensive metabolic transformation^19^ which leads to the formation of a final metabolite, named PST3093. A recent study from our laboratory^22^ indicates that PST3093 behaves as a selective SERCA2a activator showing a longer half-life than istaroxime and it is likely to account for the lusitropic effect of istaroxime in patients; nonetheless, the presence of the oxime function may still limit the chronic usage of PST3093. The main aim of this work has been the rational design and synthesis of bioisosteric PST3093 analogues with metabolically stable groups replacing the oxime function, with the purpose to maintain the selective activity on SERCA2a. We present here the ligand-based rational design, the synthesis, and the biochemical and pharmacological in vitro and in vivo characterization of a new class of alkene-based PST3093 derivatives that turned out to have selective activity on SERCA2a.

## Results

### Ligand-Based Rational Design and Synthesis of PST3093-Derived Compounds

**Figure 1:**
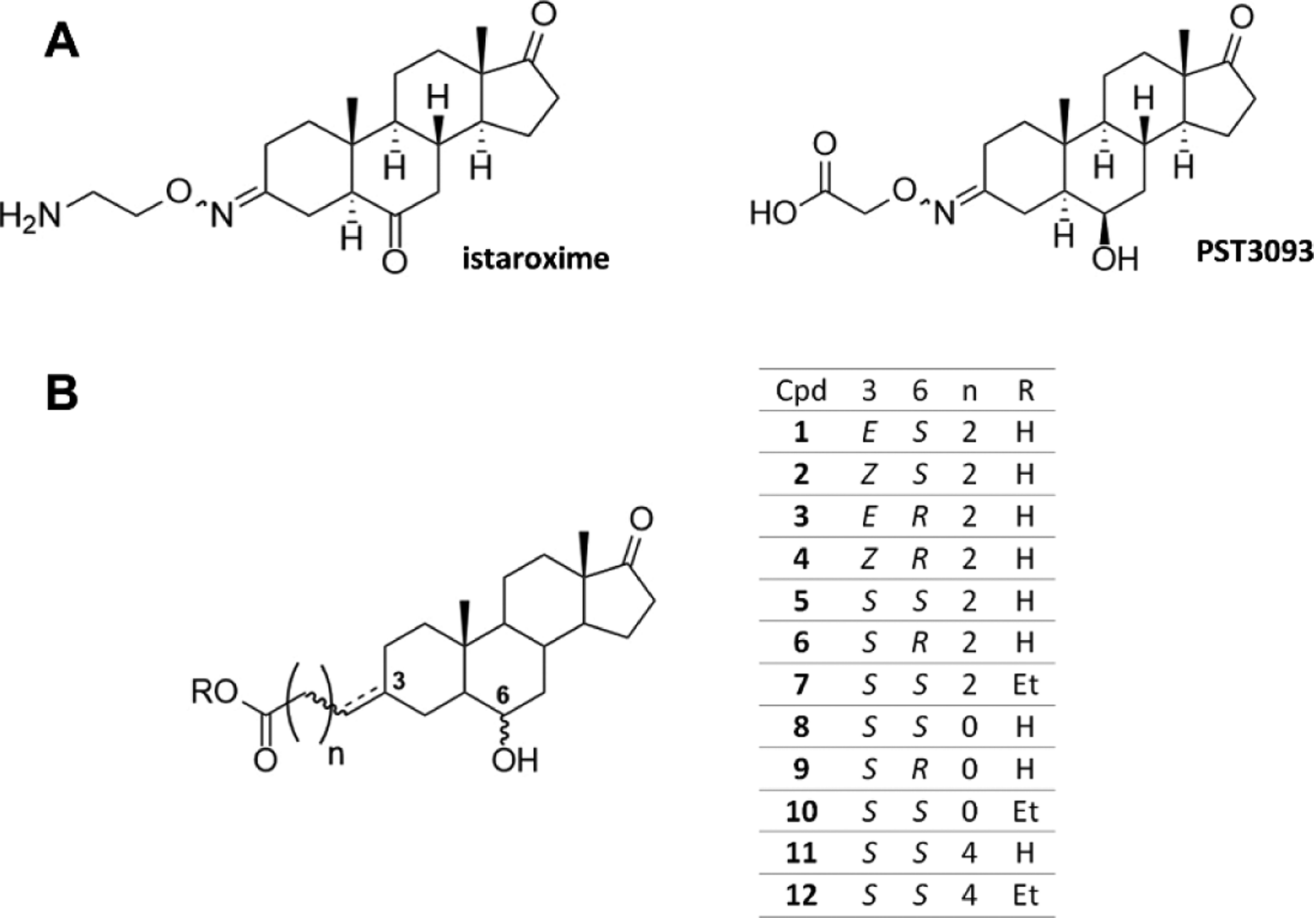
Chemical structures of (A) istaroxime and its metabolite PST3093, and (B) PST3093 synthetic variants **1–12**.

The metabolite PST3093 (Figure 1A) possesses a 17-androstanone core of istaroxime with two main structural differences: a carboxylic acid group instead of amino group on the C3-oxime linker and a hydroxyl group with *R* configuration (or β-configuration) at C6 instead of istaroxime’s carbonyl. A series of PST3093 variants (compounds **1–12**, Figure 1B) has been designed by a ligand-based approach to carry a carboxylic group (acid or ethyl ester) attached through a linker to the C3 position of the 6-hydroxy-17-oxo androstane core. Although structural information and quantitative structure–activity relationship data allowed the identification of the pharmacophore of istaroxime derivatives that binds to Na^+^/K^+^ ATPase,^16,23^ no structural information is available on the interaction between compound PST3093 and SERCA2a and/or PLN. The ligand-based design of compounds **1–12** involved the selective variation of substituents at positions C3 and C6, allowing the pharmacophore identification. The 12 compounds presented here belong to a larger library of synthetic androstane derivatives that were screened for their SERCA2a activity. The need to replace the oxime function with a bioisosteric group guided the design of all derivatives. Namely, the oxime double bond in steroid C3 was replaced with an alkene in compounds **1–4** (in the *Z* or *E* configuration) and a saturated C–C bond in the β configuration in compounds **5–12**.

The single or double carbon–carbon bonds in C3 would ensure a greater metabolic stability in all compounds compared to PST3093. The length of the C3 linker was also varied: a four-atom linker is present in compounds **1–7** exactly reproducing the PST3093 structure, while a shorter two-atom linker is present in compounds **8**, **9** and **10**, and a longer six-atom linker is present in compounds **11** and **12**. All synthetic compounds have a hydroxyl group at C6 as in PST3093: compounds **3**, **4**, **6**, and **9** are in the (*R*) configuration as PST3093, all the others are in the (*S*) configuration.

**Scheme 1:**
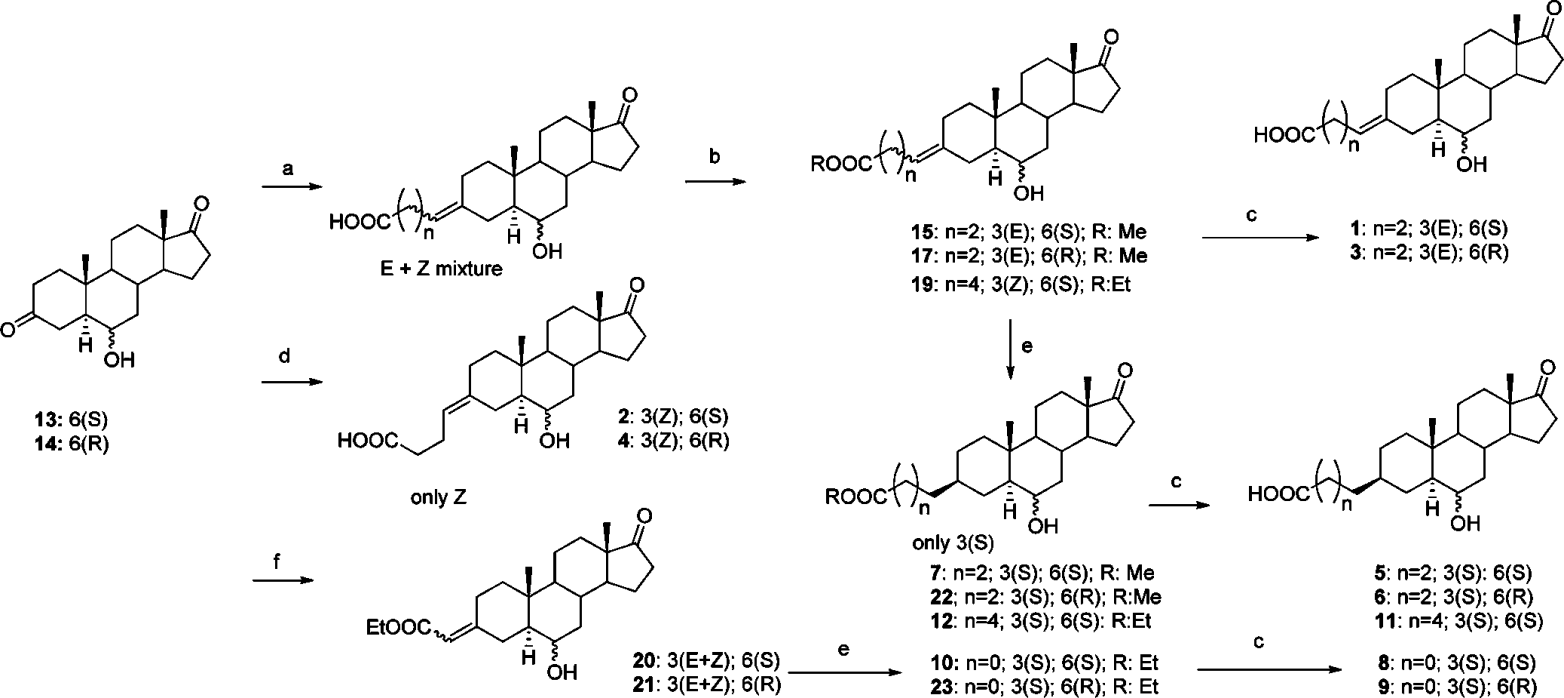
Synthesis of Compounds **1–12** from the Common Precursors **13** and **14** Reagents and conditions: (a) NaH, (3-carboxypropyl)triphenylphosphonium or (3-carboxypentyl)triphenylphosphonium bromide, DMSO. (b) EDC, EtOH. *E*/*Z* 1:2. (c) aq LiOH 1 M, THF. (d) LiHMDS, (3-carboxypropyl)triphenylphosphonium bromide, THF. (e) H_2_/Pd–C, EtOAc. (f) triethylphosphonoacetate, NaH, dimethylformamide.

Compounds **1–12** were synthesized through the synthetic strategy depicted in Scheme 1. Compounds **13** and **14**, 6*R*- and 6*S*-hydroxyandrostane-3,17-dione, respectively, were prepared as described from the commercially available prasterone (3β-hydroxyandrost-5-en-17-one).^16^ The lower reactivity of C17 carbonyl compared to that of C3 allowed for the regioselective reaction of the C3 ketones in Wittig or Horner–Emmons reactions, obtaining the insertion of the carboxylic acid or ester functionalities at position C3.

Compounds **1** and **3** were synthesized by reacting compounds **13** and **14**, respectively, with (3-carboxypropyl)triphenylphosphonium bromide and sodium hydride in dry dimethylsulfoxide (DMSO), giving the corresponding carboxylic acids (mixture of *E* + *Z* isomers 1:2 at C3 alkene) as products of the Wittig reaction that were converted into methyl esters by dissolving in methanol and treating with *N*-(3-dimethylaminopropyl)-*N*-ethylcarbodiimide (EDC) and 4-(dimethylamino)pyridine. The *E* and *Z* isomers of esters were separated by chromatography, giving compounds **15** and **17,** respectively. Ethyl ester hydrolysis with aqueous lithium hydroxide in tetrahydrofuran (THF) afforded compounds **1** and **3**. The same Wittig reaction using (3-carboxypentyl)triphenylphosphonium bromide followed by ethanol esterification gave compound **19** as a mixture of *E* + *Z* isomers. Although the use of sodium hydride as a base for the Wittig reaction gave a mixture of *E* + *Z* isomers, by reacting **13** and **14** with the same phosphonium salt in the presence of lithium bis(trimethylsilyl)amide (LiHMDS) in THF, it was possible to obtain stereoselectively only the *Z* olefins, compounds **2** and **4**. The reaction of **13** and **14** with triethylphosphonoacetate in the presence of sodium hydride (Horner–Wadsworth–Emmons reaction) gave the esters **20** and **21** (*E* + *Z* mixture).

The catalytic hydrogenation (H_2_, Pd/C) of compounds **15**, **17**, **19**, **20**, and **21** afforded compounds **7**, **22**, **12**, **10**, and **23,** respectively (Scheme 1). Interestingly, the hydrogenation at the C3 carbon–carbon double bond was found to be completely stereoselective for all derivatives, affording only the β (*S*) isomers. This is very likely due to the steric hindrance of the C19 methyl group on the upper face of the androstane A ring. Hydrolysis of the esters **7**, **22**, **12**, **10**, and **23** by treatment with aqueous lithium hydroxide in THF gave final carboxylic acids **5**, **6**, **11**, **8**, and **9**, respectively.

### New Synthetic Compounds Do Not Affect Na^+^/K^+^ ATPase and Stimulate SERCA2a in a PLN-dependent Way

It has been recently shown that although istaroxime inhibits the Na^+^/K^+^ ATPase activity (IC_50_ 0.14 μM in dog renal preparations), its metabolite PST3093 is inactive against Na^+^/K^+^ ATPase. In contrast, both molecules improve disease-induced SERCA2a depression with a similar potency.^22^ To assess whether the new PST3093 derivatives retain the pharmacological activity of the parent compound, Na^+^/K^+^ ATPase and SERCA2a activities were evaluated in in vitro assays.

**Figure 2:**
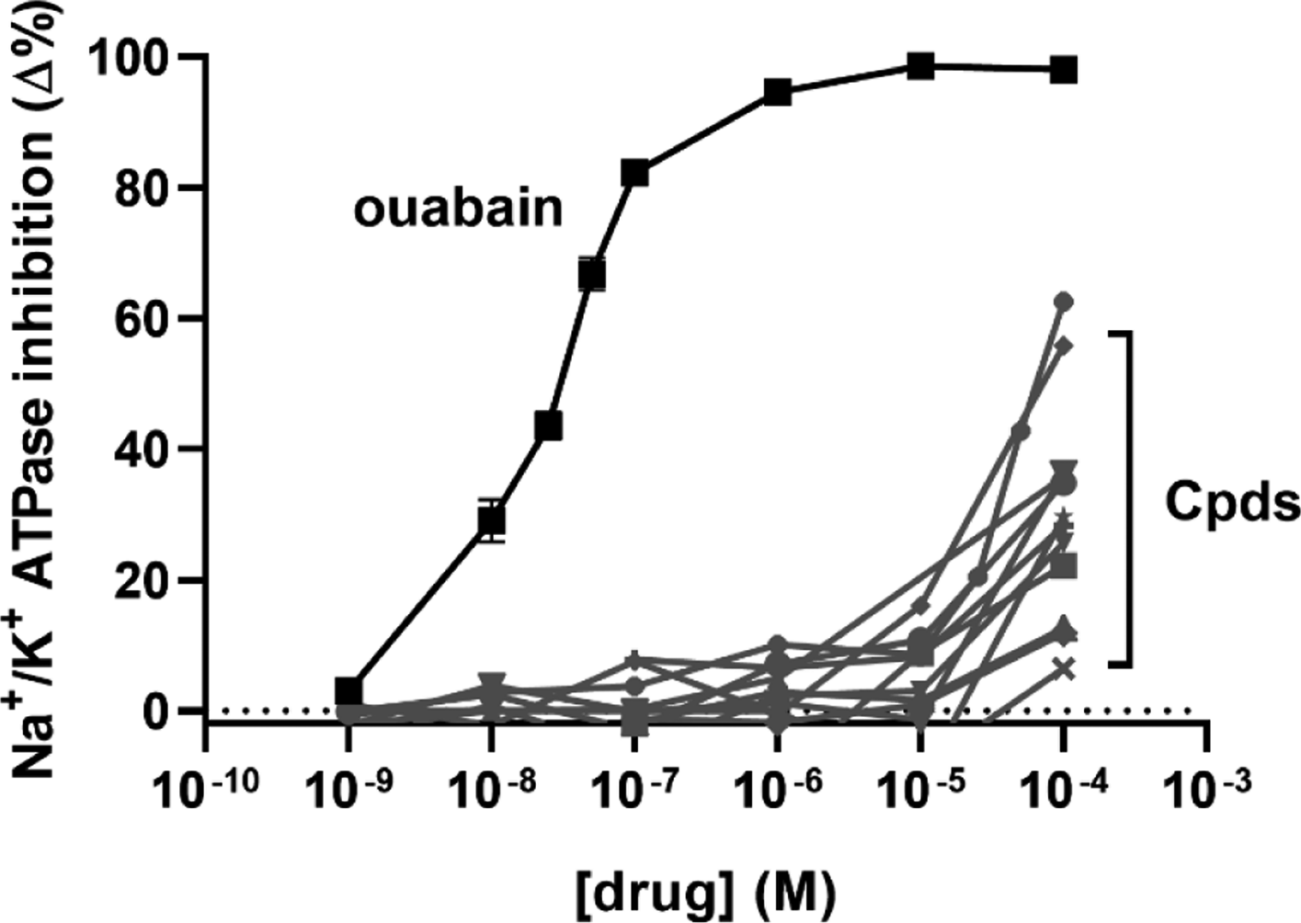
Na^+^/K^+^ ATPase activity inhibition in a dog purified enzyme preparation. All compounds were tested in a concentration range from 10^–9^ to 10^–4^ M in comparison to ouabain. (•) Cpd 9 (−62% at 10^–4^ M), (⧫) Cpd 3 (−55% at 10^–4^ M), and all the other compounds showed <40% inhibition at 10^–4^ M (*N* = 2).

Compounds were tested in a concentration range from 10^–9^ to 10^–4^ M on a purified renal Na^+^/K^+^ ATPase preparation with a specific activity of 14 μmol/min/mg protein. None of the tested molecules inhibited Na^+^/K^+^ ATPase activity up to 10^–4^ M (Figure 2), similar to PST3093.^22^

**Figure 3:**
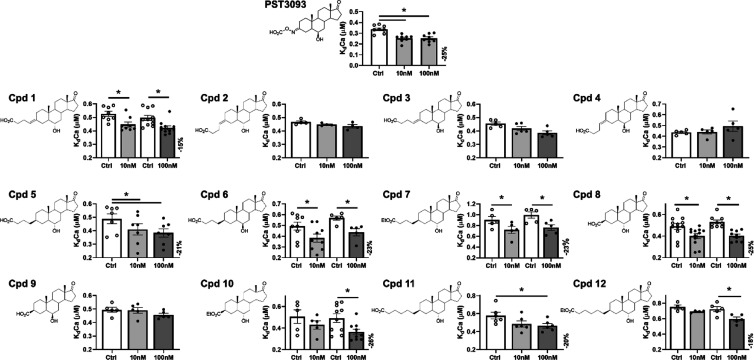
Screening of the compounds by testing their activity on SERCA2a Ca^2+^ dependency in guinea pig microsomal preparations. Concentration dependency of SERCA2a Ca^2+^ affinity (*K*_d_Ca) modulation by all compounds and PST3093 (*N* = 4–11). All compounds were tested at the concentrations of 10 and 100 nM. PST3093, compounds **5** and **8** were also tested at 1 nM (see text). Data are the mean ± SEM. *p < 0.05 (one-way RM ANOVA plus post hoc Tukey’s multiple comparisons or paired *t*-test).

SERCA2a ATPase activity was assessed in SR preparations from healthy guinea pig hearts. Ca^2+^ dependency of ATPase activity was measured, and kinetic parameters (*K*_d_Ca and *V*_max_) were estimated at compound concentrations of 10 and 100 nM. Four compounds (**2**, **3**, **4**, and **9**) were inactive, while the other eight compounds significantly increased SERCA2a–Ca^2+^ affinity (decreased *K*_d_Ca) at nanomolar concentrations (Figure 3). The maximal effect of compounds on *K*_d_Ca reached −26% at 100 nM, close to that of PST3093 (−25% at 100 nM). No compound affected SERCA2a *V*_max_ activity in healthy guinea pig SR preparations.

Compounds **5–8** and **10** had similar potencies in increasing SERCA2a–Ca^2+^ affinity (i.e., in stimulating SERCA2a). The activity of compounds **5** and **8** on SERCA2a was further investigated at 1 nM in comparison to PST3093. Also, at this concentration, compounds **5** and **8** increased SERCA2a–Ca^2+^ affinity (*K*_d_Ca reduced by 16%, *N* = 7, *p* < 0.05 and 14%, *N* = 12, *p* < 0.05, respectively) similar to PST3093 (18%, *N* = 8, *p* < 0.05), without affecting *V*_max_; this makes them good candidates for SERCA2a activation.

**Figure 4:**
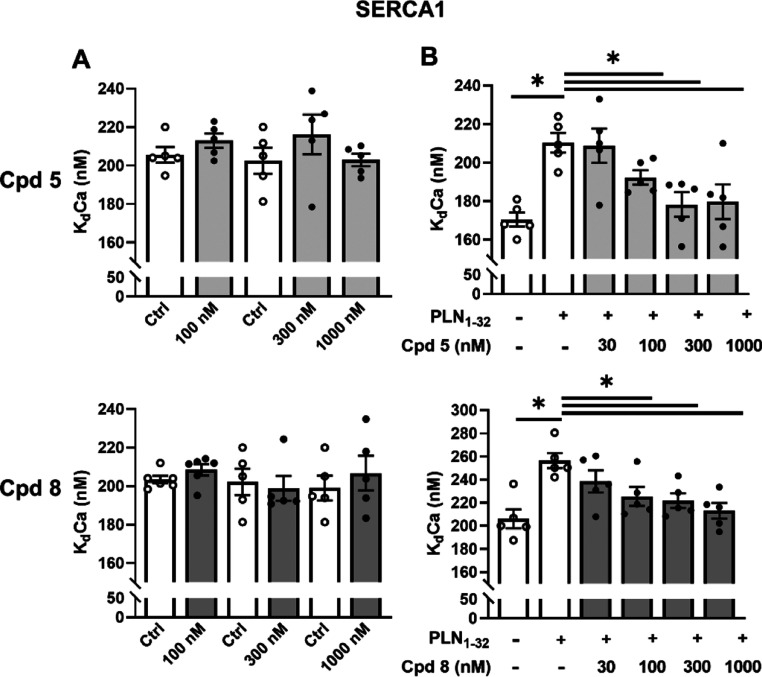
Effects of compounds **5** and **8** on SERCA1 ATPase activity and its PLN dependency in guinea pig microsomal preparations. Concentration dependency of SERCA1–Ca^2+^ affinity (*K*_d_Ca) modulation with compounds **5** and **8** in skeletal muscle microsomes containing SERCA1 alone (A) and after reconstitution with PLN_1–32_ fragments (B) (*N* = 5). Data are the mean ± SEM. *p < 0.05 (one-way RM ANOVA plus post hoc Tukey’s multiple comparisons or paired *t*-test).

Istaroxime^24^ and PST3093^22^ stimulated SERCA2a by relieving SERCA2a–PLN interaction. In a range of concentrations from 30 to 1000 nM, compounds **5** and **8** failed to affect skeletal SERCA1 activity in the absence of PLN (Figure 4A). As expected, reconstitution with the PLN_1–32_ fragment markedly reduced SERCA1 affinity for Ca^2+^ (*K*_d_Ca increased by 23–25%), without affecting *V*_max_. Under this condition, compounds **5** and **8** dose-dependently reversed the PLN-induced shift in *K*_d_Ca, leaving SERCA1 *V*_max_ unchanged, as previously reported for istaroxime^24^ and PST3093.^22^ The present results indicate that in the absence of PLN, SERCA1 is insensitive to compounds **5** and **8**; however, sensitivity is restored after reconstitution of SERCA1 with PLN, suggesting that the compounds act by weakening SERCA–PLN interaction, similar to istaroxime^24^ and PST3093.^22^

Compounds **5** and **8** were further characterized by testing their effects in cardiac preparations from a diabetic rat model [streptozotocin (STZ)-induced] with impaired SERCA2a function.^22,25^

**Figure 5:**
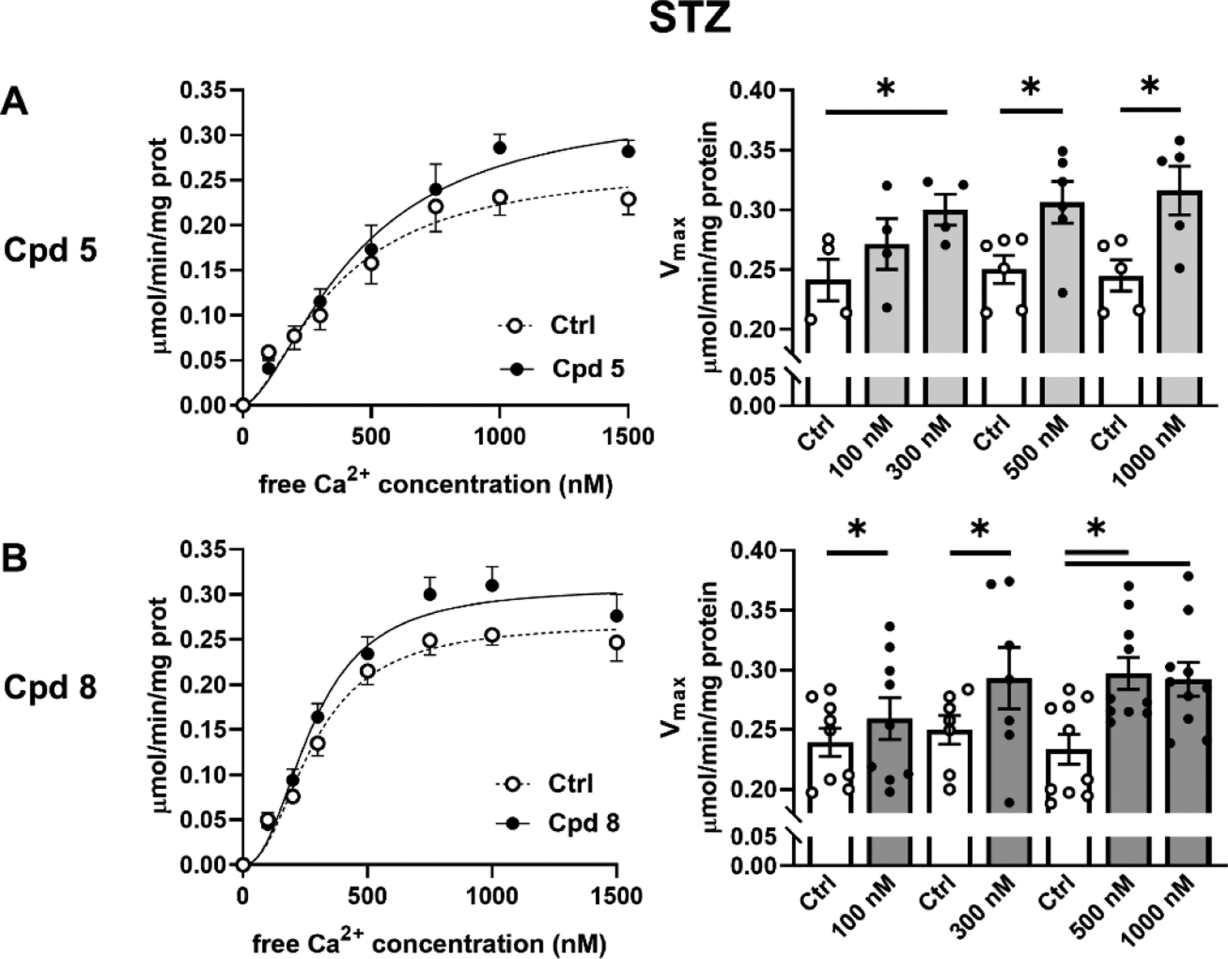
Modulation of SERCA2a ATPase activity in diseased cardiac preparations. Effect of compounds **5** (A) and **8** (B) (concentration range from 100 to 1000 nM) on SERCA2a maximal activity (*V*_max_) in cardiac homogenates from diabetic (STZ) rats (*N* = 4–10). Data are the mean ± SEM. *p < 0.05 (one-way RM ANOVA or paired *t*-test).

Consistent with our previous reports,^22,25^ baseline SERCA2a *V*_max_ activity was 30% lower in STZ (0.239 ± 0.012 μmol/min/mg protein, *N* = 9) than in healthy rats (0.343 ± 0.02 μmol/min/mg protein, *N* = 8, *p* < 0.05); SERCA2a *K*_d_Ca was instead unchanged in STZ rats (healthy 259 ± 22 nM, STZ 293 ± 23 nM, NS). Thus, as previously reported,^22,25^*V*_max_ may represent a better readout of SERCA2a activity in this species. Over the whole range of concentrations tested, compounds **5** and **8** increased SERCA2a *V*_max_ in STZ rats (+26% and +25%, +17% and +28%, at 300 and 500 nM, respectively) (Figure 5), thus reversing STZ-induced SERCA2a depression. SERCA2a *K*_d_Ca was unchanged by all compounds. Both compounds failed to affect ATPase Ca^2+^ dependency in healthy rats in terms of both parameters *K*_d_Ca and *V*_max_ (Table S1).

In summary, compounds **5** and **8** displayed similar potency in recovering disease-induced depression of SERCA2a ATPase activity in STZ rats, as previously shown for istaroxime^25^ and PST3093.^22^

The absence of effects on Na^+^/K^+^ ATPase implies that compounds **5** and **8** may represent new selective SERCA2a activators, similar to their precursor PST3093. Compound **5** was selected for further in vitro and in vivo effects.

### Compound **5** Stimulates SR Ca^2+^ Uptake in Isolated STZ Cardiomyocytes

A proof-of-principle evidence that compound **5** stimulates SERCA2a was provided by testing its effects on SR Ca^2+^ uptake function in isolated STZ cardiomyocytes through the “SR loading” protocol. This protocol (see Methods, Figure S2 and refs (22) and (25)) is suitable to assess SR Ca^2+^ uptake kinetics following caffeine-induced SR depletion under conditions emphasizing the SERCA2a role, that is, in the absence of the Na^+^/Ca^2+^ exchanger (NCX) function. In particular, through this protocol, we recently showed^22^ that voltage-induced SR Ca^2+^ reloading is significantly depressed in STZ myocytes, a functional readout of the depressed SERCA2a function in this model.

**Figure 6:**
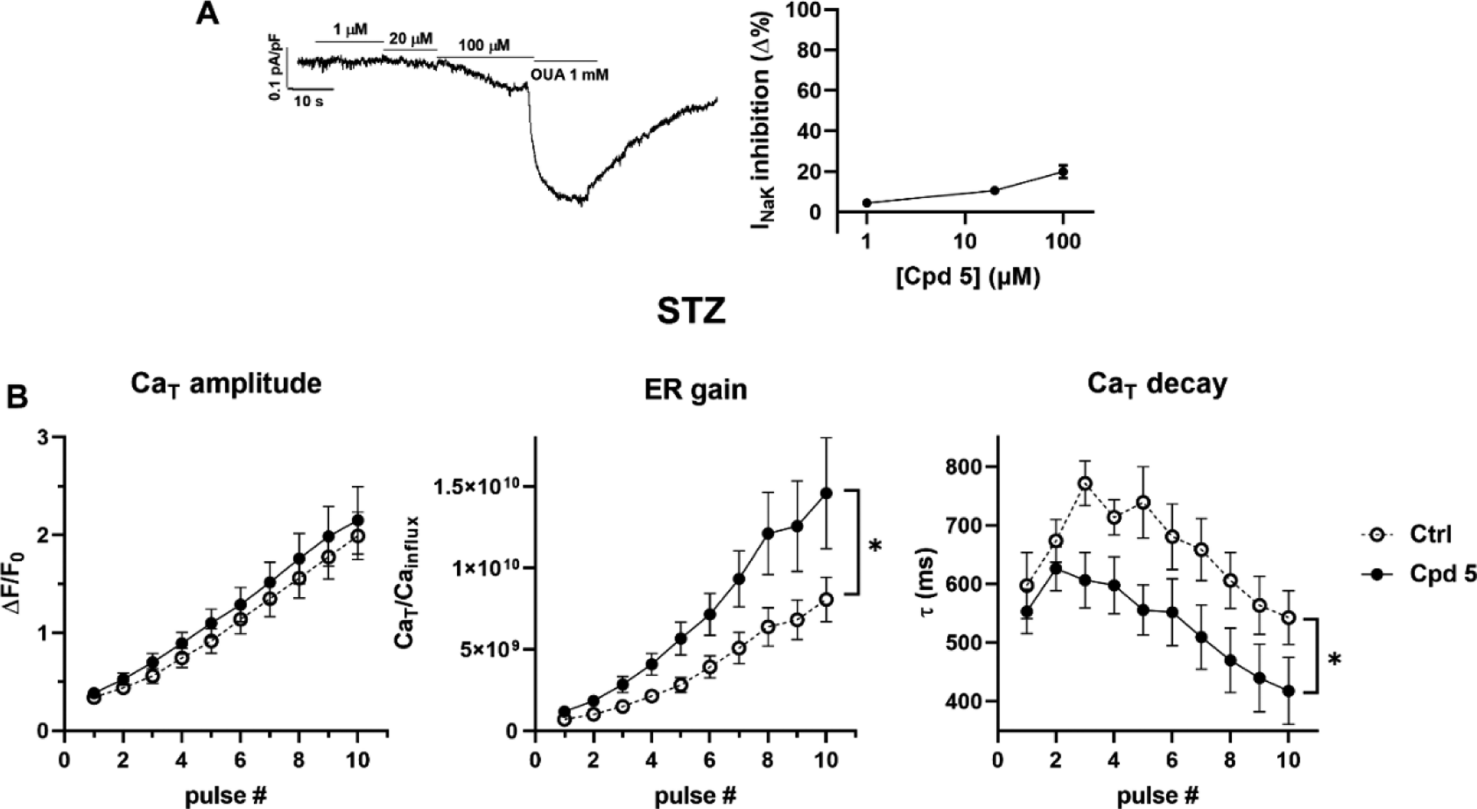
Modulation of SR Ca^2+^ uptake under NCX inhibition in V-clamped myocytes from STZ hearts. (A) Na^+^/K^+^ ATPase current (*I*_NaK_) inhibition by compound **5** (*n* = 22) in rat LV myocytes; *I*_NaK_ recording at increasing concentrations of compound **5** and finally to ouabain (OUA as reference) is shown on the left. Data are the mean ± SEM. (B) Effect of compound **5** on SR Ca^2+^ loading in patch-clamped STZ myocytes. SR Ca^2+^ loading by a train of V-clamp pulses was initiated after caffeine-induced SR depletion; NCX was blocked by Na^+^ substitution to identify SERCA2a-specific effects (see Methods and Figure S2); *N* = 3, ctrl *n* = 14, with compound 5, *n* = 11. Panels from left to right: Ca_T_ amplitude, ER gain (the ratio between Ca_T_ amplitude and Ca^2+^ influx through *I*_CaL_), and the time constant (τ) of Ca_T_ decay. *p ≤ 0.05 for the “interaction factor” in RM two-way ANOVA, indicating a different steepness of curves.

Cells were incubated for at least 30 min with compound **5** at 1 μM, a concentration not affecting Na^+^/K^+^ ATPase. Indeed, as shown in Figure 6A, in isolated rat cardiomyocytes, a detectable inhibition of the Na^+^/K^+^ ATPase current (*I*_NaK_) was observed at concentrations higher than 20 μM (−19.8 ± 3.1% at 100 μM, *N* = 22), likely for PST3093.^22^

In STZ myocytes, compound **5** (1 μM) sharply accelerated Ca^2+^ transient (Ca_T_) decay (reducing the Ca_T_ decay time constant) and increased excitation release (ER) gain at each pulse of the reloading protocol; Ca_T_ amplitude was not significantly affected by the drug (Figure 6B). Comparable results have been obtained with 1 μM PST3093.^22^

Overall, compound **5** stimulates SR function in diseased myocytes, most likely through SERCA2a enhancement, within the context of an intact cellular environment.

### In Vivo Administration of Compound **5** is Safe and Improves STZ-Induced Diastolic Dysfunction

#### Toxicity

Compound **5** was selected to evaluate in vivo effects of the new class. Acute toxicity (LD_50_) was preliminarily evaluated in mice following i.v. and oral drug administration. For i.v. administration, compound **5** had an LD_50_ of 300 mg/kg; for comparison, the LD_50_ of PST3093 and istaroxime was >250^22^ and 23 mg/kg,^22^ respectively. Oral toxicity of compound **5** was >800 mg/kg, as compared to >200 and 200 mg/kg for PST3093 and istaroxime, respectively. The main signs of toxicity were prostration, gasping, and convulsions. No overt signs of acute toxicity were observed in the surviving animals.

Collectively, the present data indicate low toxicity of compound **5**, particularly as compared to i.v. istaroxime. This is likely due to lack of inhibitory activity on the Na^+^/K^+^ pump, which also applies to istaroxime after its first-pass conversion to PST3093 in the case of oral administration.

#### In Vivo Hemodynamics

Features of the STZ-induced diabetic cardiomyopathy have been previously assessed by comparing morphometric, echocardiographic, and cellular parameters between healthy and STZ-treated rats under urethane anesthesia.^22^ Echo measurements in STZ rats indicated primarily an impairment of diastolic function, evidenced by decreased early filling velocity (*E*), increased E wave deceleration time over the *E* ratio (DT/*E*), and decreased protodiastolic TDI relaxation velocity (*e*′). The systolic function was moderately altered, as shown by larger LV end-systolic diameter (LVESD), reduced ejection fraction, depressed fractional shortening (FS) and systolic tissue velocity (*s*′).^22^

**Figure 7:**
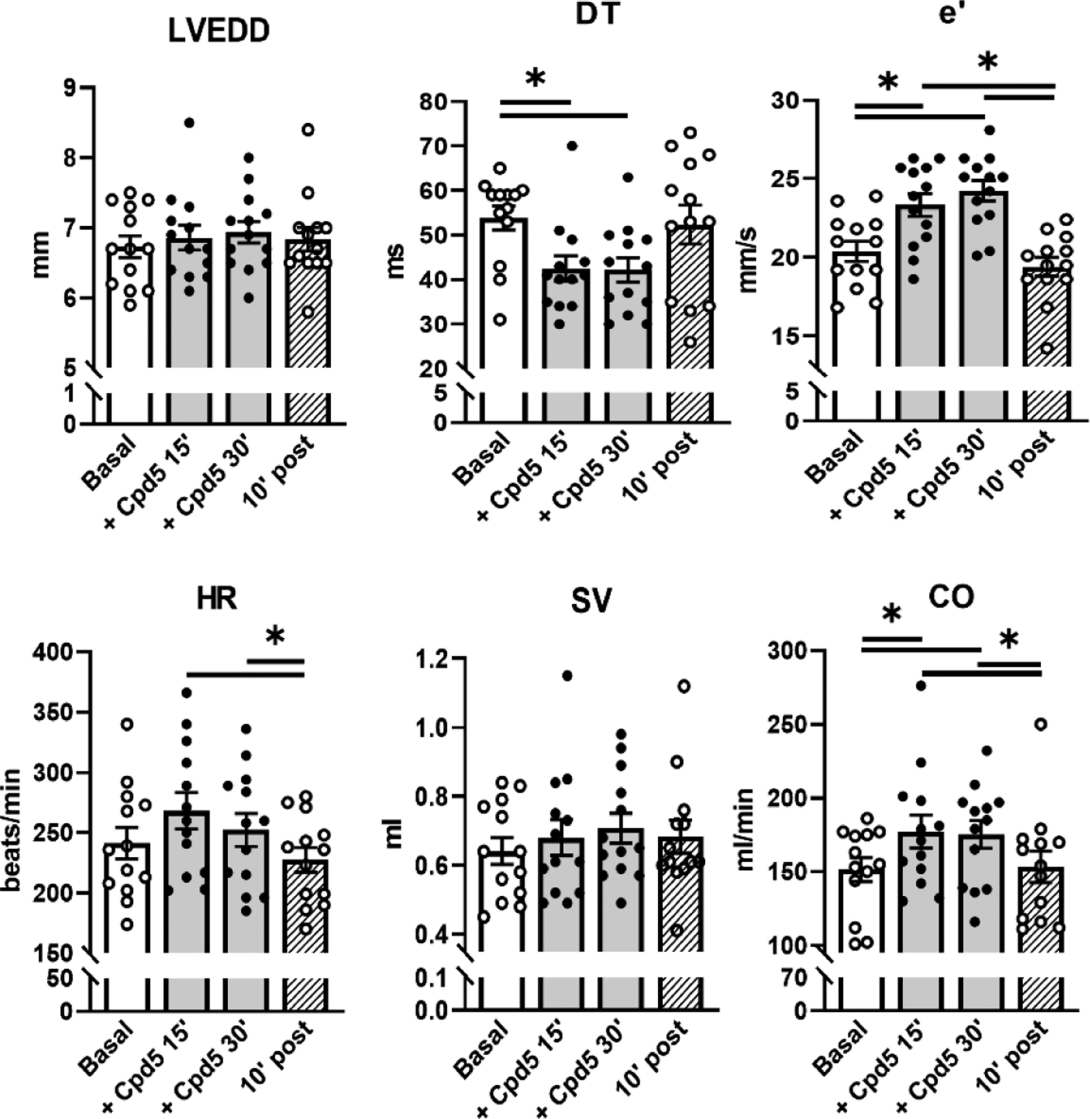
Effect of compound **5** on in vivo echocardiographic parameters in STZ diabetic rats. Compound **5** was i.v. infused (0.2 mg/kg/min) in rats 8 weeks after STZ treatment. Echocardiographic parameters were measured before (basal) and at 15 and 30 min during drug infusion and 10 min after drug interruption under urethane anesthesia. Data are the mean ± SEM; all the results of echocardiographic indexes are summarized in Table S2; *N* = 13; *p < 0.05 (one-way RM ANOVA).

Compound **5** was i.v. infused in STZ rats at a rate of 0.2 mg/kg/min, and the effects on echo parameters were investigated at 15 and 30 min of infusion and 10 min after discontinuation (Figure 7 and Table S2 for all echo parameters). Compound **5** positively affected transmitral Doppler flow indexes by increasing E and A waves; it shortened the DT, reduced the DT/*E* ratio, and increased both protodiastolic (*e*′) and telediastolic (*a*′) TDI relaxation velocities. Compound **5** increased CO, without significantly affecting HR or systolic indexes, such as FS, systolic TDI velocity (*s*′), or LVESD. Drug effects reached a plateau at 15 min of infusion; 10 min after discontinuation of the infusion, most echo indexes affected by the compound returned to the basal level.

To summarize, in vivo hemodynamic data indicate a specific lusitropic effect of compound **5** in STZ rats, compatible with rescue of SERCA2a function and largely comparable to the in vivo effect of the parent drug PST3093.^22^

## Discussion and Conclusions

In spite of the intense research targeting the discovery of small molecules or gene therapy aimed at selectively activating SERCA2a, recognized to be depressed in HF, no promising clinical outcomes have been reached so far. Therefore, there is still a compelling medical need for a compound with positive lusitropic activity. In this context, we successfully developed a new class of derivatives of PST3093 (the long-lasting metabolite of istaroxime)^22^ with a selective stimulatory action on SERCA2a but devoid of inhibitory activity on Na^+^/K^+^ ATPase. This was achieved by replacing the metabolically unstable oxime of PST3093 with more stable saturated and unsaturated carbon–carbon bonds, without altering the molecular geometry. Biological effects of the new compounds have been investigated by in vitro and in vivo assays.

The in vitro tests on SERCA2a clearly point out that replacing the C=N group of PST3093 with an alkene C=C, the activity on SERCA2a is partially retained in the case of *E* isomers (Figure 3). Indeed, although compound **1** (100 nM), carrying the alkene bond in the *E* configuration, reduced SERCA2a *K*_d_Ca by 15%, compound **2** with the same bond in the *Z* configuration was inactive. However, albeit in the *E* configuration, compound **3** was less active than compound **1**, that is, it showed only a trend to SERCA2a *K*_d_Ca reduction. For comparison, PST3093 (100 nM) reduced SERCA2a *K*_d_Ca by 25% in the same guinea pig preparation. The fact that compounds with C=C in the *E* configuration are active while *Z* isomers are inactive parallels the observation that the oxime isomer *E* is the most active in istaroxime.^16^ The replacement of oxime with a saturated C–C bond in C3 in the beta configuration led to compounds **5–12**. Compounds **5–8** and **10**, in which the C3 linker is two- and four-carbon atom long, have similar activities. Compounds **11** and **12** with a six-carbon linker are slightly less active.

Among the compounds with significant SERCA2a stimulatory activity, compounds **5** and **8**, had the best balance between potency and efficiency of chemical synthesis; thus, they were selected as leads for further evaluations. At nanomolar concentrations, the new compounds enhanced in vitro SERCA2a activity in healthy guinea pig preparations and in diseased (STZ) rat preparations; furthermore, the stimulatory effect on SERCA2a depended on the presence of PLN. This pattern is superimposable on that previously reported for the parent compound PST3093 and suggests that the new compounds may also act by partially relieving SERCA2a from PLN-induced inhibition.^22^

SR Ca^2+^ uptake function in diseased (STZ) myocytes was stimulated by compound **5** at a concentration not affecting Na^+^/K^+^ ATPase. According to the protocol specificity, this effect is surely attributable to SERCA2a stimulation under conditions of depressed function (STZ-induced SERCA2a downregulation). The parent compound PST3093 also in this case shows similar effects.^22^

In vivo studies investigated the effects of compound **5** on cardiac function in STZ diabetic rats, a disease model characterized by diastolic dysfunction, as assessed by echocardiography.^22^ Compound **5** i.v. infusion at a single dose improved diastolic echo indexes and, because of a small increase in the heart rate, cardiac output; however, it did not affect systolic function significantly (Table S2). Although improvement of diastolic indexes mimics the effect of the parent compound, PST3093 also improved systolic indexes.^22^ Similar to compound **5**, PST3093 does not inhibit the Na^+^/K^+^ pump; therefore, the reason for this difference remains to be verified.

Evaluation of in vivo acute toxicity after i.v. administration yielded an LD_50_ of compound **5** comparable to that of PST3093 whereas the oral one was higher, indicating a lower toxicity. The difference between i.v. and oral toxicity may result from the absence of enteric transit/absorption and first-pass hepatic metabolism, which characterize the former administration route. The remarkable reduction of istaroxime toxicity when orally administered can be accounted for by its fast conversion to PST3093, missing Na^+^/K^+^ pump inhibition.

Overall, the new PST3093 derivatives provide a tool for pharmacological enhancement of SERCA2a function, leading to improvement of in vivo diastolic function. With respect to PST3093, its derivatives are devoid of the oxime function and thus suitable for chronic usage and have a lower acute oral toxicity. They further differ from istaroxime, the progenitor of “PLN antagonists” already tested for clinical use because of lack of Na^+^/K^+^ pump inhibition. Considering the proarrhythmic potential of the latter, this may represent a substantial advantage in terms of safety; nonetheless, at variance with istaroxime, the new compounds should be seen as purely “lusitropic” agents, that is, devoid of “inotropic” effects beyond that expected from systo–diastolic coupling.

Although the mechanism of action of PST3093 and synthetic analogues has still to be investigated, the reversal of electrostatic properties from cationic ammonium for istaroxime to anionic carboxylate for PST3093 and analogues could account for the inactivity toward Na^+^/K^+^ ATPase.

Because PST3093 has a half-life (∼10 h) much longer than that of istaroxime (∼1 h)^22^ and its derivatives have been obtained by introducing groups with higher chemical stability, pharmacokinetics of the new compounds is likely compatible with chronic oral dosing. Nonetheless, suitability of the new compounds for chronic therapy, their natural destination, remains to be directly tested.

## Experimental Section

Methods are succinctly described here; details are given in the Supporting Information.

### Animal Models

All experiments involving animals confirmed to the guidelines for Animal Care endorsed by the Milano-Bicocca and Chang Gung Universities and to the Directive 2010/63/EU of the European Parliament on the protection of animals used for scientific purposes.

### Chemistry

#### General

All reagents and solvents were purchased from commercial sources and used without further purification. Reactions were carried out under an argon atmosphere unless otherwise noted and were monitored by thin-layer chromatography performed over silica gel 60 F_254_ plates (Merck), revealed using UV light or with molybdate staining [molybdate phosphorus acid and Ce(IV) sulfate in 4% H_2_SO_4_]. Flash chromatography purifications were performed on silica gel 60 (40–63 μm) purchased from commercial sources. ^1^H and ^13^C NMR spectra of compounds were recorded with Bruker ADVANCE 400 with TopSpin software or with NMR Varian 400 with VnmrJ software. Chemical shifts are reported in parts per million (ppm) relative to the residual solvent; coupling constants are expressed in Hz. The multiplicity in the ^13^C spectra was deduced from an attached proton test pulse sequence; peaks were assigned with the help of 2D-COSY and 2D-HSQC and 2D-NOESY experiments. Exact masses were recorded with Orbitrap Fusion Tribrid. Purity of final compounds was about 95% as assessed by high-performance liquid chromatography analysis. Reaction conditions and complete compounds characterization are reported in the Supporting Information.

### Biochemical Measurements

Total ATPase activity was assessed by measuring the rate of ^32^P-ATP release (μmol/min/mg protein) at 37 °C.

#### Na^+^/K^+^ ATPase Activity Assay

The inhibitory effect of compounds was tested at multiple concentrations on Na^+^/K^+^ ATPase-alpha1 isoform purified from dog kidneys. Na^+^/K^+^ ATPase activity was identified as the ouabain (1 mM)-sensitive component of total one; compound efficacy was expressed as the concentration exerting 50% inhibition (IC_50_).

#### SERCA ATPase Activity Assay

Measurements were performed in cardiac SR microsomes (guinea pig) and in whole tissue homogenates (rat). Cardiac preparations included SERCA2a and PLN. To test for PLN involvement in the effect of compounds, SERCA1 activity was measured in PLN-free microsomes (from guinea pig skeletal muscle) before and after reconstitution with the synthetic PLN_1–32_ inhibitory fragment at a ratio of 300:1 for PLN/SERCA. The SERCA component, identified as the cyclopiazonic acid (10 μM)-sensitive one, was measured at multiple Ca^2+^ concentrations (100–1500 nM),^13^ and Ca^2+^ dose-response curves were fitted to estimate SERCA maximal hydrolytic velocity (*V*_max_, μmol/min/mg protein) and the Ca^2+^ dissociation constant (*K*_d_Ca, nM). Either an increase in *V*_max_ (rat) or a decrease in *K*_d_Ca (increased Ca^2+^ affinity) (guinea pig) stands for enhancement of SERCA function.

### Functional Measurements in Isolated Cardiomyocytes

LV myocytes were isolated from healthy and STZ rats as previously described^26^ with minor modifications.

#### Na^+^/K^+^ ATPase Current (*I*_NaK_)

*I*_NaK_ was recorded in isolated myocytes from healthy rats as the holding current at −40 mV under conditions enhancing *I*_NaK_ and minimizing contamination by other conductances.^27^*I*_NaK_ inhibition by compound **5** was expressed as percentage reduction of ouabain (1 mM)-sensitive current.

#### Intracellular Ca^2+^ Dynamics

Ca^2+^-dependent fluorescence (Fluo4-AM) was recorded in patch-clamped LV myocytes from STZ rats and quantified by normalized units (*F*/*F*_0_). The SR Ca^2+^ uptake rate was evaluated through a “SR loading” voltage protocol, specifically devised to examine the system at multiple levels of SR Ca^2+^ loading and to rule out NCX contribution. Current through the L-type Ca^2+^ channel (*I*_CaL_) was simultaneously recorded, and the ER “gain” was calculated as the ratio between Ca_T_ amplitude and Ca^2+^ influx through *I*_CaL_ up to the Ca_T_ peak^22,25^ (protocol in Figure S2).

### In Vivo Studies

#### Acute Drug Toxicity Studies in Mice

Acute toxicity of compound **5** was preliminarily evaluated in male Albino Swiss CD-1 mice for determining the dose causing 50% mortality (LD_50_, mg/kg body weight) at 24 h after i.v. injection or oral treatment. Compound **5** was dissolved in saline solution and i.v. injected at 100, 200, 250, 275, and 300 mg/kg (one–four animals for each group) or orally administered at 100, 200, 400, 600, and 800 mg/kg (four animals for each group). Control animals received the vehicle only.

Acute toxicity following oral treatment with istaroxime and PST3093 was also evaluated for comparison; istaroxime was administered at 30, 100, and 300 mg/kg (four animals for each group) and PST3093 at 200 mg/kg (four animals for each group).

#### Hemodynamic Studies in Rats with Diabetic Cardiomyopathy

Diabetes was selected as the in vivo model because of its association with reduced SERCA2a function.^22,25^ Diabetes was induced in Sprague Dawley male rats (150–175 g) by a single i.v. STZ group (50 mg/kg in citrate buffer) injection in the tail vein. Control (healthy group) rats received vehicle (citrate buffer). Fasting glycaemia was measured after 1 week, and rats with values >290 mg/dL were considered diabetic.^22,25^

Rats were studied by transthoracic echocardiographic under anesthesia (1.25 g/kg urethane, i.p). Left-ventricular end-diastolic (LVEDD) and end-systolic (LVESD) diameter, posterior wall (PWT), and interventricular septal thickness (IVST) were measured according to the American Society of Echocardiography guidelines.^16^ FS was calculated as FS = (LVEDD – LVESD)/LVEDD. Trans-mitral flow velocity was measured (pulsed Doppler) to obtain early and late filling velocities (*E* and A waves) and E wave deceleration time (DT). DT was also normalized to E wave amplitude (DT/*E* ratio). Peak myocardial systolic (*s*′) and diastolic velocities (*e*′ and *a*′) were measured at the mitral annulus by tissue Doppler imaging (TDI).

Compound **5** was i.v. infused at 0.2 mg/kg/min (0.16 mL/min); echocardiographic parameters were measured before and at 15 and 30 min during the infusion and 10 min after drug discontinuation.

### Statistical Analysis

Individual means were compared by unpaired or paired *t*-test; multiple means were compared by one-way ANOVA for repeated measurements (RM) followed by post hoc Tukey’s multiple comparisons. Data are reported as mean ± SEM; *p* ≤ 0.05 defined statistical significance of differences in all comparisons. The number of animals or cells are specified in each figure legend.

## Supporting Information Available

Synthetic procedures, characterization of all compounds and intermediates (assignments of ^1^H and ^13^C NMR spectra, HRMS, and HPLC spectra), supplementary methods (animal models, biochemical measurements, functional measurements in isolated myocytes, and in vivo studies), supplementary tables (PDF).
Molecular formula strings (CSV).

## Abbreviations

APT: attached proton test
2D-COSY: two-dimension homonuclear correlation spectroscopy
CO: cardiac output
CPA: cyclopiazonic acid
Cpd: compound
2D-HSQC: two-dimension heteronuclear single quantum correlation
2D-NOESY: two-dimension nuclear Overhauser effect spectroscopy
DMF: dimethylformamide
DMSO: dimethylsulfoxide
DT: E wave deceleration time
*E*: early filling velocity
*e*′: TDI relaxation velocity
EDC: *N*-ethyl-*N*′-(3-dimethylaminopropyl) carbodiimide
EF: ejection fraction
EtOAc: ethyl acetate
EtOH: ethanol
FS: fractional shortening
HPLC: high-performance liquid chromatography
HF: heart failure
i.p.: intraperitoneal
i.v.: intravenous
HR: heart rate
IVST: interventricular septal thickness
LD_50_: lethal dose 50%
LVEDD: left-ventricular end-diastolic diameter
LVESD: left-ventricular end-systolic diameter
LiHMDS: lithium bis(trimethylsilyl)amide
PLN: phospholamban
PWT: posterior wall thickness
QSAR: quantitative structure–activity relationship
*s*′: systolic tissue velocity
SERCA: sarcoplasmic reticulum calcium ATPase
SR: sarcoplasmic reticulum
STZ: streptozotocin
SV: stroke volume
TDI: tissue Doppler imaging
THF: tetrahydrofuran
TLC: thin-layer chromatography
