# Supplementary material for: Highly Selective SERCA2a Activators: Preclinical Development of a Congeneric Group of First-in-Class Drug Leads against Heart Failure
Source: J Med Chem. 2022 May 17;65(10):7324–33. doi: 10.1021/acs.jmedchem.2c00347 (PMC9150102; doi:10.1021/acs.jmedchem.2c00347)
Supplement: Supplementary file 1 — jm2c00347_si_001.pdf [file jm2c00347_si_001.pdf]

Supporting Information

Highly selective SERCA2a activators: preclinical development of a congeneric group of first-in-class drug leads against heart failure

Andrea Luraghi[1], Mara Ferrandi[2], Paolo Barassi[2], Martina Arici[1], Shih-Che Hsu[3], Eleonora Torre[1], Carlotta Ronchi[1], Alessio Romerio[1], Gwo-Jyh Chang[4], Patrizia Ferrari[2], Giuseppe Bianchi[2,5], Antonio Zaza[1*], Marcella Rocchetti[1*], Francesco Peri[1*]

[1]Department of Biotechnology and Biosciences, University of Milano-Bicocca, 20126 Milano, Italy
[2]Windtree Therapeutics Inc., Warrington, Pennsylvania, 18976 USA
[3]CVie Therapeutics Limited, Taipei, 11047 Taiwan
[4]Cardiovascular Medicine, Chang Gung University, Tao-Yuan, 333323 Taiwan
[5]Università Vita-Salute San Raffaele, 20132 Milano, Italy

Corresponding authors:
francesco.peri@unimib.it; marcella.rocchetti@unimib.it; antonio.zaza@unimib.it

**CONTENTS**	**PAGES**
**Chemistry**	**S2-S10**
Scheme S1	*S2*
Scheme S2	*S2*
Figure S1	S3
**NMR spectra**	**S11-S30**
**HPLC spectra**	**S31**
**Supplementary methods**	**S32-S36**
Animal models	S32
Biochemical measurements	S32
Functional measurements in isolated myocytes	S34
Figure S2	S35
In-vivo studies	S36
**Supplementary table S1**	**S37**
**Supplementary table S2**	**S38**
**References**	**S39-S40**

Chemistry

General. All reagents and solvents were purchased from commercial sources and used without further purifications, unless stated otherwise. Reactions were monitored by thin-layer chromatography (TLC) performed over Silica Gel 60 F254 plates (Merck®). Flash chromatography purifications were performed on silica gel 60 40-63µm from commercial source. ^{1}H and ^{13}C NMR spectrum were recorded with Bruker Advance 400 with TopSpin® software, or with NMR Varian 400 with Vnmrj software. Chemical shifts are expressed in ppm respect TMS; coupling constants are expressed in Hz. The multiplicity in the ^{13}C spectra was deducted by APT experiments. Exact masses were recorded with Orbitrap Fusion™ Tribrid™.

Synthesis of 13 and 14

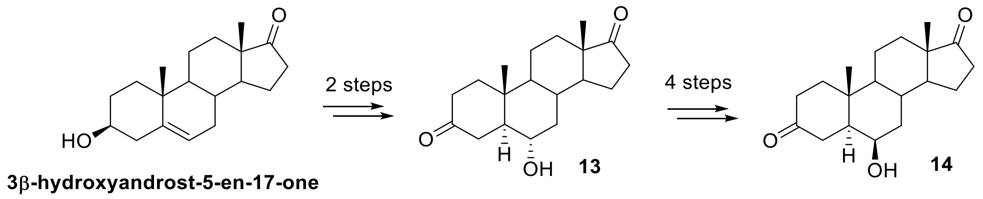

Scheme S1 Synthesis of **13** and **14** from commercial prasterone as previously published Common intermediates **13** and **14** were synthesized from commercial prasterone (3β-hydroxyandrost-5-en-17-one) already published[1].

Synthesis of compounds 1-12

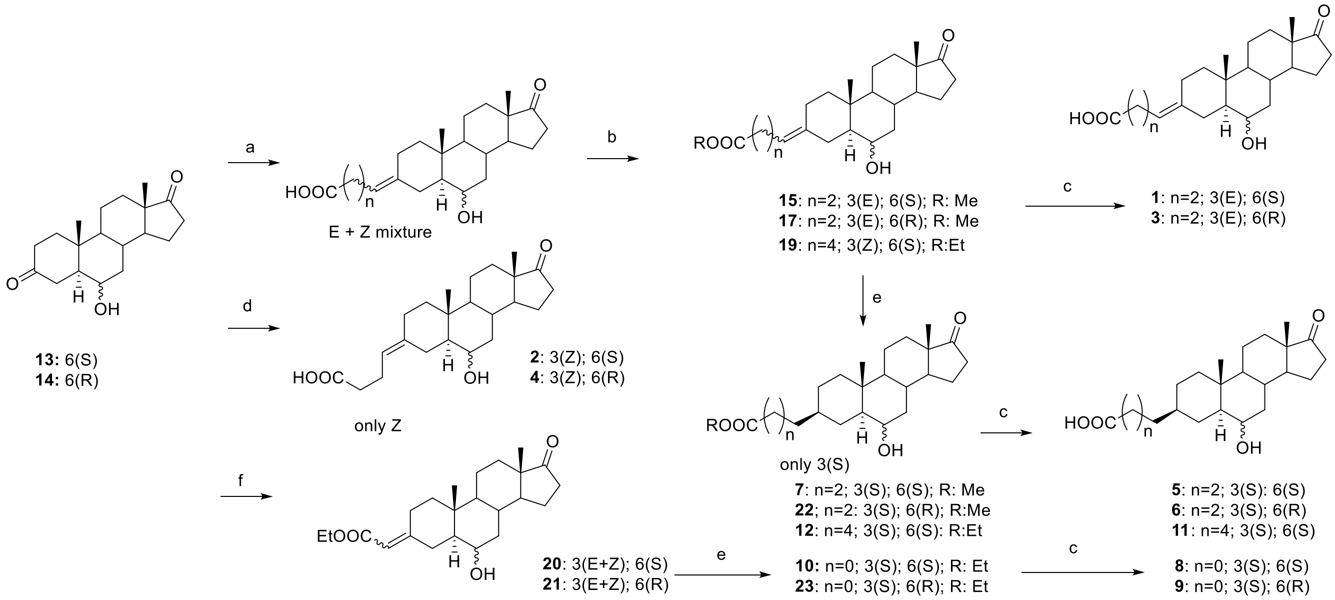

Scheme S2 Synthesis of compounds **1-12** from intermediates **13** and **14.** Reagents and conditions: a) NaH, HOOC-(CH_2)n-P(Ph)$_3$$^+Br^-$ (n = 2,4), DMSO. b) EDC, EtOH. E:Z 1:2, separation of isomers. c) aq. LiOH 1M, THF, H_2O. d) LiHMDS, (HOOC-(CH_2)$_2$-P(Ph)$_3$$^+Br^-$, THF. e) H_2/Pd-C, EtOAc.f) triethylphosphonoacetate, NaH, DMF.

Compounds numbering

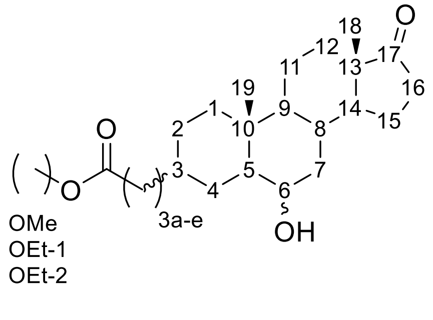

Figure S1 general position numbering for compounds **1-23**

Wittig reaction (NaH/DMSO protocol) + ester formation (conditions a+b)

NaH 60% in mineral oil (4 eq.) was carefully added to dry DMSO (1 mL) under Ar atmosphere, the resulting solution was stirred at 60°C for 20 minutes. After cooling at room temperature, phosphonium salt (4 eq.) was added. A bright orange color appears immediately. The solution was stirred for 2h then compounds **13** or **14** (1 eq.) were added to the mixture. The resulting solution was allowed to stir at room temperature for additional 4h. The reaction mixture diluted with EtOAc (25mL) was washed with aq. 1M HCl (3x30mL). The organic layer dried over

Na_2SO_4 was evaporated to dryness obtaining 25mg of crude material. The crude was dissolved in EtOH (1.5mL) then EDC hydrochloride (2eq.) and DMAP (5mg, 0.03mmol, 0.1 eq.) were added. The solution was stirred at room temperature for 3h. After concentration in vacuo. The crude solid was dissolved in EtOAc (15mL) and washed with aq. 1M HCl (3x10mL). The crude product was purified by flash chromatography over silica gel.

Compound **13** was reacted with (3-Carboxypropyl)triphenylphosphonium bromide in the conditions described obtaining an E + Z = 1:2 mixture (**15**)

Methyl (3 E, 6S)-4-(6-hydroxy-17-oxoandrostane-3-yliden)butyrate (**15**) was obtained in 85% yield from **13**:

^{1}H NMR (400 MHz, cdcl$_3$) δ 5.14 – 5.04 (m, 1H, H-3α), 3.66 (s, 3H, OMe), 3.46 (td, *J* = 10.8, 4.3 Hz, 1H, H-6), 2.91 (d, *J* = 13.7 Hz, 1H, H-16a), 2.45 (dd, *J* = 19.3, 8.9 Hz, 3H, H-16b), 2.39 – 2.25 (m, 5H), 2.17 – 2.02 (m, 3H), 1.96 (q, *J* = 12.2 Hz, 3H), 1.79 (dd, *J* = 16.5, 13.2 Hz, 3H), 1.66 (d, *J* = 11.2 Hz, 3H), 1.62 – 1.46 (m, 5H), 1.38 – 1.17 (m, 9H), 1.10 – 1.01 (m, 1H), 0.95 (td, *J* = 10.2, 5.5 Hz, 3H), 0.89 (s, 3H, Me-18), 0.86 (s, 3H, Me-19), 0.74 (tt, *J* = 8.0, 4.0 Hz, 1H).

m/z calculated for $[C_{24}H_{36}NaO_4]^+$ 411.2506. Found: 411.2509.

Ethyl (3Z, 6S)-4-(6-hydroxy-17-oxoandrostane-3-yliden)butyrate (**16**):

^{1}H NMR (400 MHz, CDCl3) δ 5.07 (t, J = 5.8 Hz, 1H, H-3α), 4.11 (q, J = 7.1 Hz, 2H, OEt-1), 3.47 (td, J = 10.8, 4.5 Hz, 1H, H-6), 2.91 (ddd, J = 13.9, 3.5, 1.7 Hz, 1H, H-16a), 2.50 – 2.36 (m, 2H, H-3γ), 2.32 (dt, J = 6.1, 1.6 Hz, 1H, H-3βa), 2.30 – 2.28 (m, 1H), 2.28 – 2.24 (m, 1H), 2.19 – 2.09 (m, 2H, H-7), 2.09 – 2.04 (m, 1H), 2.01 – 1.90 (m, 1H, H-3βb), 1.82 – 1.76 (m, 1H), 1.72 (ddd, J = 12.6, 4.8, 2.5 Hz, 1H), 1.64 (dd, J = 11.9, 3.3 Hz, 2H, H-15), 1.60 (d, J = 4.1 Hz, 1H), 1.52 (ddd, J = 12.4, 9.0, 3.3 Hz, 1H), 1.37 – 1.26 (m, 3H), 1.28 – 1.20 (m, 4H, OEt-2), 1.03 – 0.90 (m, 3H, H-5), 0.88 (s, 3H, Me-18), 0.85 (s, 3H, Me-19), 0.71 (td, J = 11.3, 4.1 Hz, 1H).^{13}C NMR (101 MHz, CDCl3) δ 173.53, 139.46, 119.80, 69.84, 60.27, 51.19, 47.74, 40.60, 40.22, 37.42, 35.78, 34.65, 32.09, 31.41, 29.67, 25.16, 22.65, 21.74, 20.21, 14.27, 13.78, 12.93.

m/z calculated for $[C_{25}H_{38}NaO_4]^+$ 425.2662. Found 425.2668.

Compound **14** was reacted with (3-Carboxypropyl)triphenylphosphonium bromide in the conditions described obtaining an E + Z = 1:2 mixture, the two isomers were separated by chromatography obtaining pure isomers **17** (E) and **18** (Z)

Methyl (3E, 6R)-4-(6-hydroxy-17-oxoandrostane-3-yliden)butyrate (**17**) was obtained in 20% yield from **14**:

^{1}H NMR (400 MHz, CDCl$_3$) δ 5.07 (s, 1H, H-3α), 3.85 (d, *J* = 2.9 Hz, 1H, H-6), 3.66 (s, 3H, OMe), 2.51 – 2.40 (m, 4H, H-16a, H-3γ), 2.32 (d, *J* = 5.8 Hz, 5H), 2.14 – 2.00 (m, 2H), 1.92 (t, *J* = 9.9 Hz, 5H), 1.81 (d, *J* = 13.5 Hz, 3H), 1.74 – 1.63 (m, 4H), 1.55 (ddd, *J* = 21.4, 12.3, 8.8 Hz, 5H), 1.40 (dd,

J = 13.0, 4.0 Hz, 2H), 1.35 – 1.16 (m, 13H), 1.10 (s, 3H, Me-18), 0.89 (s, 3H, Me-19), 0.86 (d, *J* = 11.7 Hz, 3H), 0.74 (t, *J* = 9.2 Hz, 1H).

m/z calculated for [$C_{24}H_{36}NaO_4^+$] 411.2506. Found: 411.2507.

Ethyl (3Z, 6R)-4-(6-hydroxy-17-oxoandrostane-3-yliden)butyrate (**18**) was obtained in 43% yield from **14**:

^{1}H NMR (400 MHz, $CDCl_3$) δ 5.07 (s, 1H, H-3α), 3.89 (d, *J* = 2.8 Hz, 1H, H-6), 3.66 (s, 3H, OMe), 2.46 (dd, *J* = 19.2, 8.8 Hz, 1H, H-16a), 2.33 (d, *J* = 3.4 Hz, 4H, H-3γ), 2.30 – 2.14 (m, 3H), 2.14 – 1.89 (m, 6H), 1.81 (d, *J* = 12.3 Hz, 1H), 1.72 – 1.63 (m, 3H), 1.62 – 1.48 (m, 4H), 1.43 – 1.13 (m, 11H), 1.10 (s, 3H, Me-19), 0.95 (td, *J* = 13.3, 4.6 Hz, 2H), 0.89 (s, 3H, Me-18), 0.88 – 0.80 (m, 2H), 0.79 – 0.68 (m, 1H).

m/z calculated for [$C_{25}H_{38}NaO_4$]$^+$ 425.2662. Found 425.2667.

Compound **13** was reacted with (3-Carboxypentoyl)triphenylphosphonium bromide in the conditions described obtaining an E + Z = 1:2 mixture, which, after estherification were separated, obtaining compound 19 (Z isomer)

Ethyl (3 Z, 6S)-4-(6-hydroxy-17-oxoandrostane-3-yliden)caproate (**19**) was obtained in 87% yield from **13**

^{1}H NMR (400 MHz, $CDCl_3$) δ 5.08 (td, *J* = 7.4, 1.8 Hz, 1H H-3α), 4.11 (q, *J* = 7.1 Hz, 2H, OEt-1), 3.45 (td, *J* = 10.8, 4.6 Hz, 1H, H-6), 2.87 (ddd, *J* = 14.0, 3.6, 1.8 Hz, 1H), 2.50 – 2.39 (dd, 1H, H-16a), 2.32 – 2.24 (m, 2H, H-3ε), 2.23 – 2.09 (m, 3H, H-7a, H-3β), 2.08 – 1.91 (m, 5H, H-16b, H-15a), 1.83 – 1.68 (m, 4H, H-3γ), 1.63 (dddd, *J* = 15.2, 10.6, 4.9, 2.5 Hz, 5H, H-3δ), 1.52 (ddd, *J* = 12.4, 9.0, 3.4 Hz, 1H, H-15b), 1.40 – 1.32 (m, 3H), 1.32 – 1.26 (m, 2H), 1.24 (t, *J* = 7.1 Hz, 6H, OEt-2), 1.05 – 0.91 (m, 3H, H-5), 0.89 (s, 3H, Me-18), 0.86 (s, 3H, Me-19), 0.73 (ddt, *J* = 13.8, 7.5, 3.6 Hz, 1H).

^{13}C NMR (101 MHz, $CDCl_3$) δ 174.18, 138.42, 121.40, 69.91, 60.28, 54.54, 54.03, 51.20, 47.76, 40.51, 40.25, 37.46, 35.78, 34.23, 33.88, 32.17, 31.42, 29.67, 29.26, 26.37, 25.21, 24.34, 21.75, 20.21, 14.23, 13.78, 12.90.

m/z calculated for [$C_{27}H_{42}NaO_4$]$^+$ 453.2975. Found 453.2980.

Wittig reaction (LiHMDS/THF protocol)

LiHMDS 1M solution in THF (6 eq.) was carefully added to a dry THF (33mL) suspension of (3-carboxypropyl)triphenylphosphonium bromide (6eq.) under Ar atmosphere at -40°C. The solution was stirred at -40°C until a bright orange color appears then compounds 13 or 14 (1 eq.) were added to the solution at -40°C. After stirring at room temperature overnight, the reaction mixture, quenched with aq. 1M HCl (30mL), was extracted with EtOAc (3x35mL). The combined organic layers were dried over Na2SO4 and evaporated to dryness. Crude material was dissolved in EtOH (1.5mL), then EDC hydrochloride (2eq.) and DMAP (5mg, 0.03 mmol, 0.1 eq.) were added. The solution was stirred at room temperature for 3h. After concentration in vacuo, the crude solid was dissolved in EtOAc

(15mL) and washed with aq. 1M HCl (3x10mL). The crude product was purified by flash chromatography over silica gel.

General ester hydrolysis procedure (Step c)

To a solution of the methyl or ethyl esters (1eq.) in THF (600µL) and water (200µL) aq. 1M LiOH (2.5eq.) was added. After 2h, the reaction was diluted with water (10mL) and quenched by the addition of 1M HCl until pH 1. The aqueous phase was extracted with EtOAc (3x15mL). The combined organic layers were dried over Na_2SO_4 and evaporated to dryness. Crude was purified over flash chromatography.

(6S,E)-4-(6-hydroxy-17-oxoandrostane-3-yliden)butyric acid (**1**) was obtained in 93% yield from **17**:

^{1}H NMR (400 MHz, $CDCl_3$) δ 5.12 (d, *J* = 7.0 Hz, 1H, H-3α), 3.46 (td, *J* = 10.9, 4.5 Hz, 1H, H-6), 2.52 – 2.39 (m, 3H, H-16a, H-3γ), 2.40 – 2.32 (m, 4H, H-3β), 2.32 – 2.25 (m, 1H), 2.15 – 2.07 (m, 2H, H-7), 1.95 (d, *J* = 13.2 Hz, 2H), 1.84 (d, *J* = 17.1 Hz, 1H), 1.81 – 1.73 (m, 2H), 1.72 – 1.62 (m, 2H), 1.56 – 1.49 (m, 1H), 1.36 – 1.30 (m, 1H), 1.30 – 1.24 (m, 2H), 1.24 – 1.18 (m, 1H), 1.05 (ddd, *J* = 13.4, 10.7, 3.5 Hz, 1H, H-5), 1.01 – 0.93 (m, 2H), 0.89 (s, 3H, Me-19), 0.86 (s, 3H, Me-18), 0.74 (td, *J* = 11.3, 4.0 Hz, 1H).

^{13}C NMR (101 MHz, $CDCl_3$) δ 217.25, 174.67, 135.65, 115.83, 65.88, 61.95, 50.83, 49.96, 47.21, 43.86, 36.34, 35.46, 33.42, 31.88, 30.48, 29.89, 29.47, 27.45, 26.36, 25.76, 19.96, 18.55, 17.82, 16.32, 11.32, 9.86, 8.99.

m/z calculated for $[C_{23}H_{33}O_4]^-$ 373.2384. Found 373.2386.

(6S,Z)-4-(6-hydroxy-17-oxoandrostane-3-yliden)butyric acid (**2**) was obtained in 87% yield from **13**:

^{1}H NMR (400 MHz, $CDCl_3$) δ 5.09 (d, *J* = 7.6 Hz, 1H, H-3α), 3.50 (td, *J* = 10.4, 3.6 Hz, 1H, H-6), 2.91 (d, *J* = 13.7 Hz, 1H, H-16a), 2.51 – 1.90 (m, 15H, H-3β, H-3γ, H-7), 1.81 (d, *J* = 11.4 Hz, 2H), 1.68 (dt, *J* = 25.3, 12.7 Hz, 6H, H-16b), 1.58 – 1.46 (m, 2H), 1.27 (q, *J* = 12.2 Hz, 8H), 0.97 (t, *J* = 11.8 Hz, 3H, H-5, H-15), 0.89 (s, 3H, Me-19), 0.86 (s, 3H, Me-18), 0.73 (dd, *J* = 15.3, 7.0 Hz, 2H).

^{13}C NMR (101 MHz, $CDCl_3$) δ 220.86, 177.66, 139.84, 119.75, 119.47, 70.08, 69.81, 54.85, 54.33, 54.01, 53.96, 51.23, 51.19, 47.76, 40.40, 40.34, 40.17, 39.45, 37.43, 37.36, 35.78, 34.23, 33.86, 33.81, 33.41, 32.10, 31.42, 29.68, 25.17, 22.52, 21.75, 20.21, 14.09, 13.78, 12.91, 0.99.

m/z calculated for $[C_{23}H_{33}O_4]^-$ 373.2384. Found 373.2388.

(6R,E)-4-(6-hydroxy-17-oxoandrostane-3-yliden)butyric acid (**3**) was obtained in 93% yield from **17**:

^{1}H NMR (400 MHz Acetone-*d*$_6$) δ 4.97 (d, J = 7.5 Hz, 1H, H-3α), 3.68 (q, J = 2.9 Hz, 1H, H-6), 2.47 (t, J = 13.5 Hz, 1H, H-4a), 2.38 (d, J = 14.3 Hz, 1H), 2.26 (dd, J = 18.1, 8.9 Hz, 1H, H-16), 2.21 – 2.08 (m, 4H, H-3β, H-3γ), 1.96 – 1.84 (m, 2H), 1.84 – 1.75 (m, 2H, H-7), 1.66 (t, J = 2.4 Hz, 1H, H-4b), 1.64 – 1.56 (m, 2H), 1.55 – 1.47 (m, 1H), 1.42 (dt, J = 10.8, 3.0 Hz, 0H), 1.38 – 1.34 (m, 0H), 1.33 – 1.26 (m, 1H), 1.26 – 1.19 (m, 1H), 1.14 – 1.08 (m, 1H), 1.06 (d, J = 4.3 Hz, 1H, H-5), 1.02 (s, 3H, Me-19), 0.84 – 0.76 (m, 1H), 0.74 (s, 5H, Me-18), 0.64 (ddd, J = 12.4, 10.5, 3.9 Hz, 1H).

^{13}C NMR (101 MHz, Acetone-d_6) δ 218.89, 205.38, 140.75, 119.30, 70.19, 54.63, 51.15, 51.10, 47.35, 40.88, 38.83, 36.74, 36.55, 35.15, 34.00, 31.73, 29.55, 24.02, 22.54, 21.54, 20.11, 14.74, 13.18.

m/z calculated for $[C_{23}H_{33}O_4]^-$ 373.2384. Found 373.2386.

(6R,Z)-4-(6-hydroxy-17-oxoandrostane-3-yliden)butyric acid (**4**) was obtained in 91% yield from **18**:

^1H NMR (400 MHz, Acetone-d_6) δ, ppm: 5.09 (s, 1H, H-3α), 3.89 (s, 1H, H-5), 2.46 (dd, $^3J_{H,H}$=19.3, 8.8 Hz, 1H, H-16a), 2.41-2.15 (m, 3H), 2.16-1.88 (m, 3H), 1.82 (d, $^3J_{H,H}$=12.6 Hz, 1H), 1.68 (d, $^3J_{H,H}$=11.5 Hz, 2H), 1.62-1.52 (m, 1H), 1.25 (q, $^3J_{H,H}$=8.0, 6.7 Hz, 7H), 1.10 (s, 3H, Me-19), 0.90 (s, 3H, Me-18), 0.74 (t, $^3J_{H,H}$=11.5 Hz, 1H, H-5).

^{13}C NMR (101 MHz, Acetone-d_6) δ, ppm: 216.39, 173.80, 135.48, 113.80, 66.52, 10.07, 8.70.

m/z calculated for $[C_{23}H_{33}O_4]^-$ 373.2384. Found 373.2387.

(3S,6S)-4-(6-hydroxy-17-oxoandrostane-3-yl)butyric acid (**5**) was obtained in 89% yield from **7**:

^1H NMR (400 MHz, $CDCl_3$) δ, ppm: 3.43 (bt, 1H, H-6), 2.44 (dd, $^3J_{H,H}$=19.2, 8.7 Hz, 1H, H-16a), 2.31 (bt, 2H, H-3γ), 0.84 (s, 3H, Me-19), 0.77 (s, 3H, Me-18).

^{13}C NMR (101 MHz, $CDCl_3$) δ, ppm: 221.35, 179.11, 69.90, 13.78, 13.37.

m/z calculated for $[C_{23}H_{35}O_4]^-$ 375.2541. Found 375.2544.

(3S,6R)-4-(6-hydroxy-17-oxoandrostane-3-yl)butyric acid (**6**) was obtained in 97% yield from **22**:

^1H NMR (400 MHz, $CDCl_3$) δ 3.85 (s, 1H, H-6), 2.45 (dd, J = 19.2, 8.9 Hz, 1H, H-16a), 2.34 (t, J = 7.4 Hz, 2H, H-3γ), 2.14 – 2.00 (m, 1H, H-16b), 1.93 (d, J = 10.8 Hz, 3H, H-7), 1.81 (d, J = 12.7 Hz, 1H), 1.64 (d, J = 13.2 Hz, 5H, H3-β, H-12), 1.53 (q, J = 11.3 Hz, 2H, H-15), 1.43 – 1.06 (m, 11H), 1.00 (s, 3H, Me-19), 0.94 (s, 1H), 0.89 (s, 3H, Me-18), 0.74 (d, J = 13.2 Hz, 1H).

^{13}C NMR (101 MHz, $CDCl_3$) δ 221.52, 179.06, 72.04, 54.56, 51.26, 49.15, 47.90, 40.07, 36.66, 36.19, 35.86, 34.12, 32.39, 31.50, 30.05, 28.62, 22.12, 21.77, 20.08, 15.76, 13.88.

m/z calculated for $[C_{23}H_{35}O_4]^-$ 375.2541. Found 375.2545.

(3S,6S)-2-(6-hydroxy-17-oxoandrostane-3-yl)acetic acid (**8**) was obtained in 91% yield from **10**:

^1H NMR (400 MHz, $CDCl_3$) δ 3.43 (td, *J* = 10.6, 4.4 Hz, 1H, H-6), 2.45 (dd, *J* = 19.3, 8.6 Hz, 1H, H-16a), 2.37 – 2.21 (m, 2H, H-3α), 2.17 – 1.91 (m, 4H), 1.80 (d, *J* = 10.2 Hz, 1H), 1.65 (t, *J* = 14.9 Hz, 3H), 1.56 – 1.43 (m, 1H), 1.41 – 1.13 (m, 6H), 1.01 (dq, *J* = 24.2, 11.4 Hz, 3H), 0.85 (s, 3H, Me-19), 0.80 (s, 3H, Me-18), 0.75 (d, *J* = 21.6 Hz, 3H).

^{13}C NMR (101 MHz, Acetone-d_6) δ 220.47, 207.29, 174.29, 68.57, 54.27, 54.14, 54.12, 53.27, 53.22, 51.14, 51.11, 50.86, 47.27, 41.46, 40.18, 38.55, 36.39, 35.18, 34.76, 34.72, 34.28, 33.73, 33.71, 31.45, 27.92, 21.39, 19.97, 12.99, 12.59.

m/z calculated for $[C_{21}H_{31}O_4]^-$ 347.2228. Found: 347.2230.

(3S,6R)-2-(6-hydroxy-17-oxoandrostane-3-yl)acetic acid (**9**) was obtained in 96% yield from **23**:^{1}H NMR (400 MHz, $CDCl_3$) δ 3.75 (q, *J* = 2.8 Hz, 1H, H-6), 2.34 (ddd, *J* = 19.1, 8.9, 1.0 Hz, 1H, H-16a), 2.18 (dd, *J* = 7.0, 4.1 Hz, 2H, H-3α), 2.01 – 1.89 (m, 1H), 1.85 – 1.75 (m, 4H), 1.73 – 1.67 (m, 1H), 1.59 – 1.47 (m, 4H), 1.46 – 1.25 (m, 5H), 1.25 – 1.07 (m, 15H), 0.90 (s, 3H, Me-19), 0.89 – 0.83 (m, 1H), 0.77 (s, 3H, Me-18), 0.76 – 0.67 (m, 3H).

^{13}C NMR (101 MHz, $CDCl_3$) δ 221.30, 177.79, 71.76, 54.45, 51.22, 48.96, 47.86, 41.47, 39.80, 38.33, 35.88, 35.82, 35.29, 32.07, 31.48, 30.04, 29.68, 28.39, 21.76, 20.07, 15.69, 13.86.

m/z calculated for $[C_{21}H_{31}O_4]^-$ 347.2228. Found: 347.2232.

(3S,6S)-4-(6-hydroxy-17-oxoandrostane-3-yl)caproic acid (**11**) was obtained in 93% yield from **12**:

^{1}H NMR (400 MHz, $CDCl_3$) δ 3.45 – 3.39 (m, 1H, H-6), 2.44 (dd, *J* = 19.2, 8.6 Hz, 1H, H-16a), 2.33 (t, *J* = 7.3 Hz, 3H, H-3ε), 2.15 – 2.01 (m, 3H, H-16b, H-7), 1.93 (td, *J* = 13.4, 7.9 Hz, 3H), 1.79 (dd, *J* = 8.8, 2.5 Hz, 1H), 1.71 – 1.57 (m, 7H, H-3δ), 1.57 – 1.44 (m, 3H), 1.31 (dt, *J* = 11.6, 5.8 Hz, 8H), 1.27 – 1.15 (m, 7H), 1.02 (dddd, *J* = 25.5, 22.4, 12.6, 6.5 Hz, 6H, H-5), 0.85 (s, 3H, Me-19), 0.83 (s, 1H), 0.77 (s, 3H, Me-18), 0.73 (d, *J* = 15.1 Hz, 1H).

^{13}C NMR (101 MHz, $CDCl_3$) δ 179.06, 69.91, 54.18, 53.36, 51.23, 47.81, 40.24, 38.80, 37.28, 35.81, 33.97, 33.84, 31.43, 29.25, 28.49, 26.44, 24.69, 21.76, 20.16, 13.79, 13.38.

m/z calculated for $[C_{25}H_{39}O_4]^-$ 403.2854. Found 403.2859.

Horner-Emmons protocol (step f)

To a suspension of NaH 60% in mineral oil (5 eq) in DMF (200 mL) under Ar atmosphere triethylphosphonoacetate (5 eq) was added carefully at 0°C. The resulting solution was warmed at room temperature and stirred for 20 minutes, then diketone **13** or **14** (1 eq) was added at 0°C. After stirring overnight at room temperature, the reaction was quenched by careful addition of H2O (100mL) and extracted with Et2O (3x150mL). The combined organic layers were dried over Na_2SO_4 and evaporated *in vacuo*. Crude was purified by flash chromatography over a column of silica gel to give a clear oil mixture of the two diasteroisomers.

ethyl (6S,EZ)-4-(6-hydroxy-17-oxoandrostane-3-yliden)acetate (**21**) was obtained in 89% yield from **13**:

^{1}H NMR (400 MHz, $CDCl_3$) δ, ppm: 5.60 (s, 1H, H-3α), 4.21-4.06 (m, 2H, OCH_2), 3.45 (td, $^3J_{H,H}$=10.8, 4.5 Hz, 1H, H-6), 2.41 (dd, $^3J_{H,H}$=19.3, 8.8 Hz, 1H, H-16a), 0.90 (s, 3H, Me-18), 0.82 (s, 3H, Me-19), 0.77-0.66 (m, 1H, H-5).

^{13}C NMR (101 MHz, $CDCl_3$) δ, ppm: 220.85, 166.8, 161.87, 113.75, 69.41, 13.76, 13.00.

m/z calculated for $[C_{23}H_{34}NaO_4]^-$ 397.2349.Found 397.2355.

ethyl (6R,EZ)-4-(6-hydroxy-17-oxoandrostane-3-yliden)acetate (**22**) was obtained in 85% yield from **14**:

^{1}H NMR (400 MHz, $CDCl_3$) δ, ppm: 5.64 (d, $^{3}J_{H,H}$=5.1 Hz, 1H, H-3α), 4.14 (q, $^{3}J_{H,H}$=7.1 Hz, 2H, OCH_2), 3.90 (dd, $^{3}J_{H,H}$=27.4, 2.8 Hz, 1H, H-6), 3.81-3.70 (m, 1H), 3.67-3.57 (m, 1H), 0.91 (s, 3H, Me-18), 0.86-0.73 (m, 1H, H-5).

^{13}C NMR (101 MHz, $CDCl_3$) δ, ppm: 221.01, 166.87, 166.72, 163.06, 162.87, 128.31, 127.88, 113.51, 113.21, 71.36, 71.19, 59.55, 54.31, 54.23, 51.16, 51.12, 50.51, 49.84, 41.40, 40.83, 38.62, 38.40, 37.35, 36.30, 35.83, 35.80, 33.35, 31.47, 30.01, 29.98, 29.33, 25.36, 21.77, 20.27, 20.22, 15.31, 15.29, 14.31, 13.88.

m/z calculated for $[C_{23}H_{34}NaO_4]^-$ 397.2349.Found 397.2353.

Hydrogenation procedure (Step e)

While under Ar atmosphere, 10% Pd-C (0.3 on weight) was added to a degassed solution of unsaturated compounds (1 eq) in EtOAc (20 mL). After three cycles of vacuum/hydrogen, the reaction was allowed to stir at room temperature overnight under H_2 atmosphere. After removal of hydrogen by vacuum/Ar cycle, the reaction mixture was filtered over CELITE®. The filtered solution was evaporated to dryness.

ethyl (3S,6S)-4-(6-hydroxy-17-oxoandrostane-3-yl)butyrate (**7**) was obtained in 90% yield from a mixture of **15** and **16**:

^{1}H NMR (400 MHz, $CDCl_3$) δ 4.09 (dtdd, *J* = 7.2, 6.1, 2.7, 1.2 Hz, 2H, OEt-1), 3.39 (ddd, *J* = 14.7, 9.5, 3.0 Hz, 1H, H-6), 2.42 (ddd, *J* = 18.9, 8.9, 2.8 Hz, 1H, H-16a), 2.30 – 2.19 (m, 2H, H-3γ), 2.11 – 2.00 (m, 2H), 1.96 – 1.85 (m, 2H), 1.82 – 1.71 (m, 2H), 1.64 (ddd, *J* = 15.5, 9.1, 6.1 Hz, 5H), 1.58 – 1.43 (m, 3H), 1.38 – 1.14 (m, 13H, OEt-2), 1.09 – 0.89 (m, 4H), 0.86 (d, *J* = 8.8 Hz, 1H), 0.82 (s, 3H, Me-18), 0.79 (d, *J* = 2.7 Hz, 1H), 0.75 (s, 3H, Me-19), 0.71 (d, *J* = 11.0 Hz, 1H).

^{13}C NMR (101 MHz, $CDCl_3$) δ 220.91, 173.77, 69.66, 69.62, 60.14, 54.17, 53.37, 51.30, 51.22, 47.74, 40.33, 38.74, 37.14, 36.92, 36.82, 35.77, 34.57, 34.41, 33.83, 33.80, 32.11, 31.43, 29.64, 29.60, 29.27, 28.27, 25.00, 23.32, 22.34, 21.73, 20.15, 14.23, 13.77, 13.36, 0.97.

m/z calculated for $[C_{25}H_{40}NaO_4]^+$ 427.2819. Found 427.2821.

ethyl (3S,6S)-(6-hydroxy-17-ketoandrostane-3beta-yl)acetate (**10**) was obtained in 93% yield from **21**:

^{1}H NMR (400 MHz, $CDCl_3$) δ 4.07 (q, *J* = 7.1 Hz, 2H, OEt-1), 3.37 (td, *J* = 10.1, 4.0 Hz, 1H, H-6), 2.40 (dd, *J* = 19.2, 8.8 Hz, 1H, H-16a), 2.19 (t, *J* = 6.5 Hz, 2H, H-3α), 2.06 (dd, *J* = 11.0, 5.3 Hz, 2H, H-7a), 2.00 (s, 2H, H-16b), 1.96 – 1.87 (m, 2H), 1.79 – 1.69 (m, 2H), 1.67 – 1.60 (m, 2H), 1.59 – 1.41 (m, 3H, H-15), 1.21 (td, *J* = 6.9, 2.8 Hz, 11H, OEt-2), 1.04 – 0.99 (m, 1H, H-5), 0.99 – 0.87 (m, 3H, H-7b), 0.81 (s, 3H, Me-18), 0.75 (s, 3H, Me-19), 0.68 (s, 1H).

^{13}C NMR (101 MHz, $CDCl_3$) δ 221.06, 172.95, 69.44, 60.39, 60.16, 53.98, 53.19, 51.14, 47.75, 41.99, 40.31, 38.44, 36.62, 35.77, 34.66, 33.80, 31.36, 29.66, 29.07, 27.95, 21.72, 21.03, 20.13, 14.25, 14.16, 13.76, 13.32.

m/z calculated for $[C_{23}H_{36}NaO_4]^+$ 399.2506. Found 399.2510.

ethyl (3S,6S)-4-(6-hydroxy-17-oxoandrostane-3-yl) caproate (**12**) was obtained in 92% yield from **19** and **20**:

^{1}H NMR (400 MHz, $CDCl_3$) δ 4.10 (qd, *J* = 7.2, 1.4 Hz, 2H, OEt-1), 3.40 (tdd, *J* = 10.8, 4.7, 1.7 Hz, 1H, H-6), 2.49 – 2.37 (m, 1H, H-16a), 2.26 (td, *J* = 7.5, 1.4 Hz, 2H, H-3ε), 2.12 – 2.07 (m, 1H, H-15a), 2.07 – 1.99 (m, 1H, H-16b), 1.99 – 1.85 (m, 2H), 1.82 – 1.74 (m, 1H), 1.73 – 1.46 (m, 9H, H-15b, H-3δ), 1.37 – 1.26 (m, 7H), 1.25 – 1.18 (m, 9H, OEt-2), 1.12 – 0.97 (m, 3H, H-5), 0.97 – 0.90 (m, 2H, H-15b), 0.86 (s, 1H), 0.84 (s, 3H, Me-18), 0.81 (d, *J* = 3.4 Hz, 1H), 0.76 (d, *J* = 1.5 Hz, 3H, Me-19), 0.74 – 0.69 (m, 1H).

^{13}C NMR (101 MHz, $CDCl_3$) δ 173.88, 69.75, 60.14, 54.25, 54.20, 53.43, 51.30, 51.24, 47.77, 40.34, 38.81, 37.33, 37.18, 36.96, 35.78, 34.33, 33.84, 33.81, 31.44, 31.39, 29.66, 29.62, 29.39, 29.33, 28.43, 26.48, 24.94, 21.74, 20.16, 14.23, 13.78, 13.38, 0.97.

m/z calculated for $[C_{27}H_{44}NaO_4]^+$ 455.3132. Found 455.3133.

methyl (3S,6R)-4-(6-hydroxy-17-oxoandrostane-3-yl)butyrate (**23**) was obtained in 90% yield from a mixture of **17** and **18**.

^{1}H NMR (400 MHz, $CDCl_3$) δ 3.83 (s, 1H, H-6), 3.68 – 3.63 (m, 3H, OMe), 2.44 (dd, *J* = 19.2, 8.9 Hz, 1H, H-16a), 2.28 (t, *J* = 7.4 Hz, 2H, H-3γ), 2.06 (dt, *J* = 18.7, 8.9 Hz, 1H, H-16b), 1.92 (d, *J* = 10.6 Hz, 3H), 1.80 (d, *J* = 13.1 Hz, 2H), 1.63 (d, *J* = 11.2 Hz, 5H, H-3β), 1.59 – 1.46 (m, 4H), 1.27 (tdd, *J* = 36.5, 13.1, 8.4 Hz, 15H, H-15), 1.14 – 1.06 (m, 1H, H-4), 0.99 (s, 3H, Me-18), 0.94 (d, *J* = 13.5 Hz, 1H), 0.88 (s, 3H, Me-19), 0.86 – 0.79 (m, 1H), 0.74 (d, *J* = 13.5 Hz, 2H).

^{13}C NMR (101 MHz, $CDCl_3$) δ 221.46, 174.27, 71.97, 54.57, 51.50, 51.26, 49.17, 47.87, 46.57, 40.07, 38.26, 38.08, 37.66, 36.77, 36.18, 35.85, 35.06, 34.34, 34.29, 32.41, 31.57, 31.50, 30.05, 29.69, 28.63, 22.36, 21.76, 20.07, 15.74, 13.86, 13.81, 1.01.m/z calculated for $[C_{24}H_{38}NaO_4]^+$ 413.2662. Found 413.2666.

ethyl (3S,6R)-(6-hydroxy-17-ketoandrostane-3beta-yl)acetate (**24**) was obtained in 95% yield from **22**

^{1}H NMR (400 MHz, $CDCl_3$) δ 4.12 (q, *J* = 7.1 Hz, 2H, OEt-1), 3.85 (q, *J* = 2.8 Hz, 1H, H-6), 2.45 (dd, *J* = 19.0, 9.1 Hz, 1H, H-16a), 2.24 (dd, *J* = 7.0, 1.2 Hz, 2H, H-3α), 2.13 – 2.01 (m, 1H, H-16b), 1.99 – 1.85 (m, 4H, H-7), 1.81 (dt, *J* = 12.7, 3.1 Hz, 1H, H-3), 1.72 – 1.29 (m, 11H, H-15), 1.28 – 1.22 (m, 7H, OEt-2), 1.22 – 1.18 (m, 1H, H-4), 1.01 (s, 3H, Me-18), 0.97 (dd, *J* = 13.2, 3.9 Hz, 1H), 0.89 (s, 3H, Me-19), 0.76 (ddt, *J* = 12.6, 6.7, 3.3 Hz, 1H).

^{13}C NMR (101 MHz, $CDCl_3$) δ 221.22, 172.96, 71.77, 60.18, 54.47, 51.24, 48.99, 47.84, 41.98, 39.83, 38.32, 35.90, 35.83, 35.50, 32.16, 31.50, 30.05, 28.40, 21.76, 21.73, 20.07, 15.68, 14.28, 13.86.

m/z calculated for $[C_{23}H_{36}NaO_4]^+$ 399.2506. Found 399.2509.

Experimental spectra of the compounds

1 ^{1}H $CDCl_3$ and ^{13}C in acetone

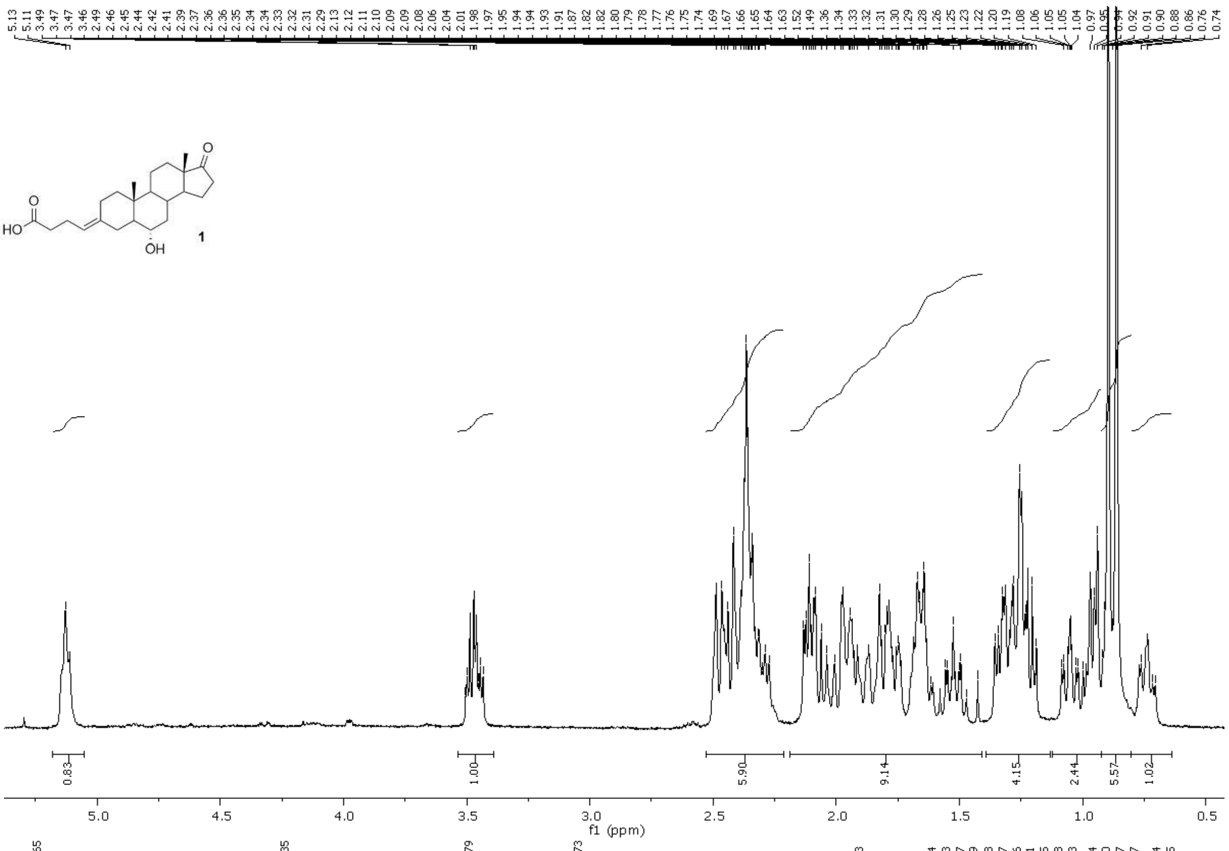

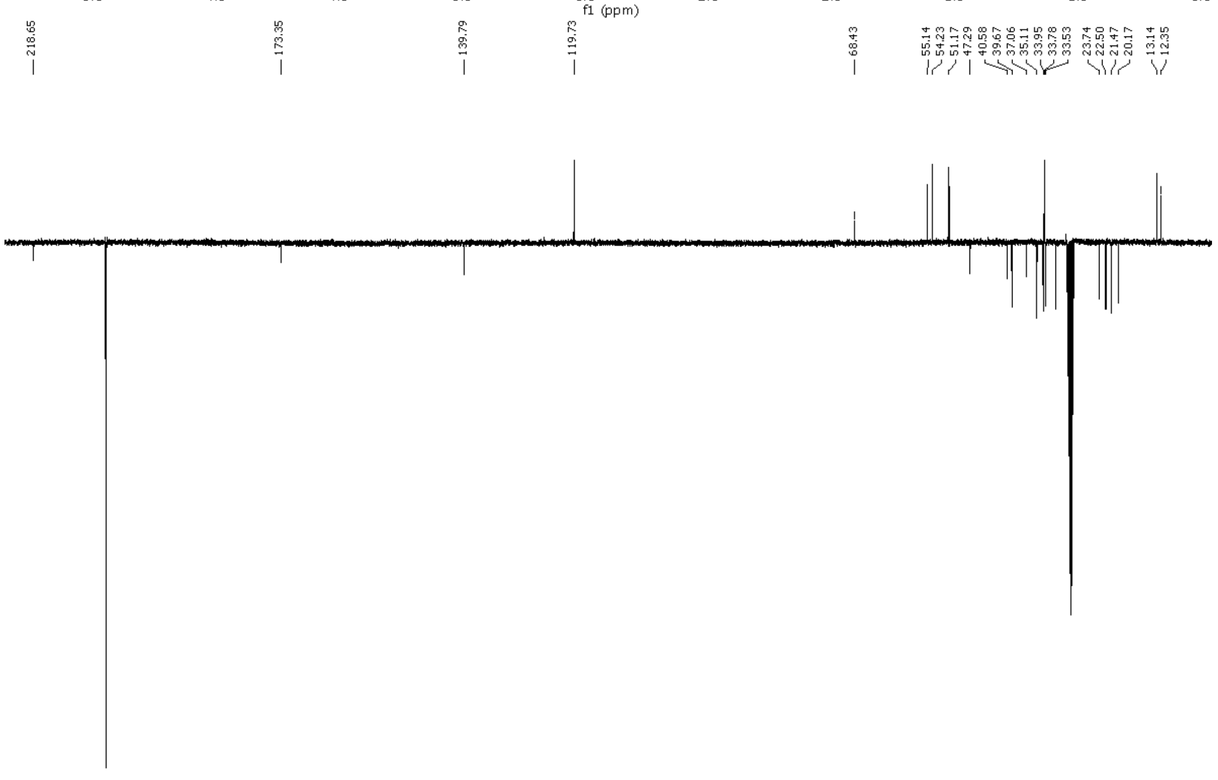

2 ^{1}H and ^{13}C CDCl$_3$

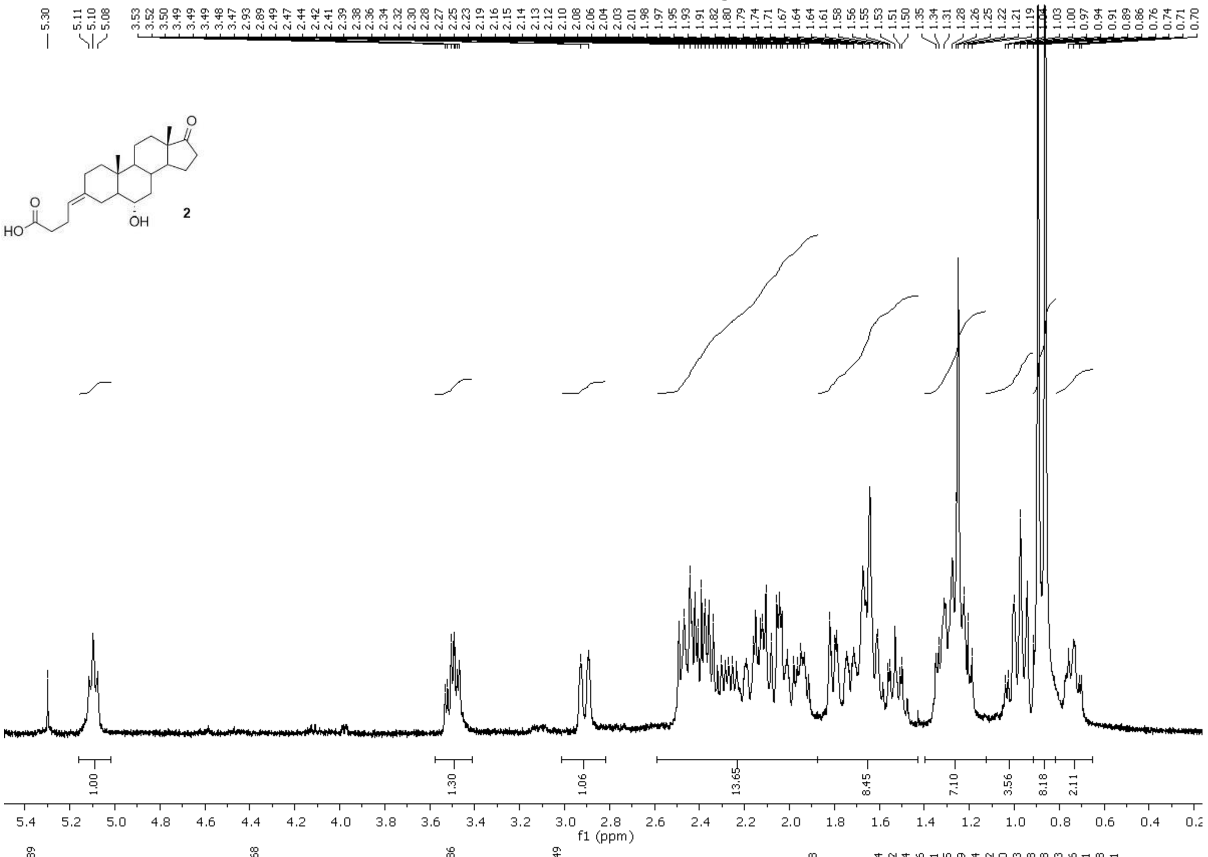

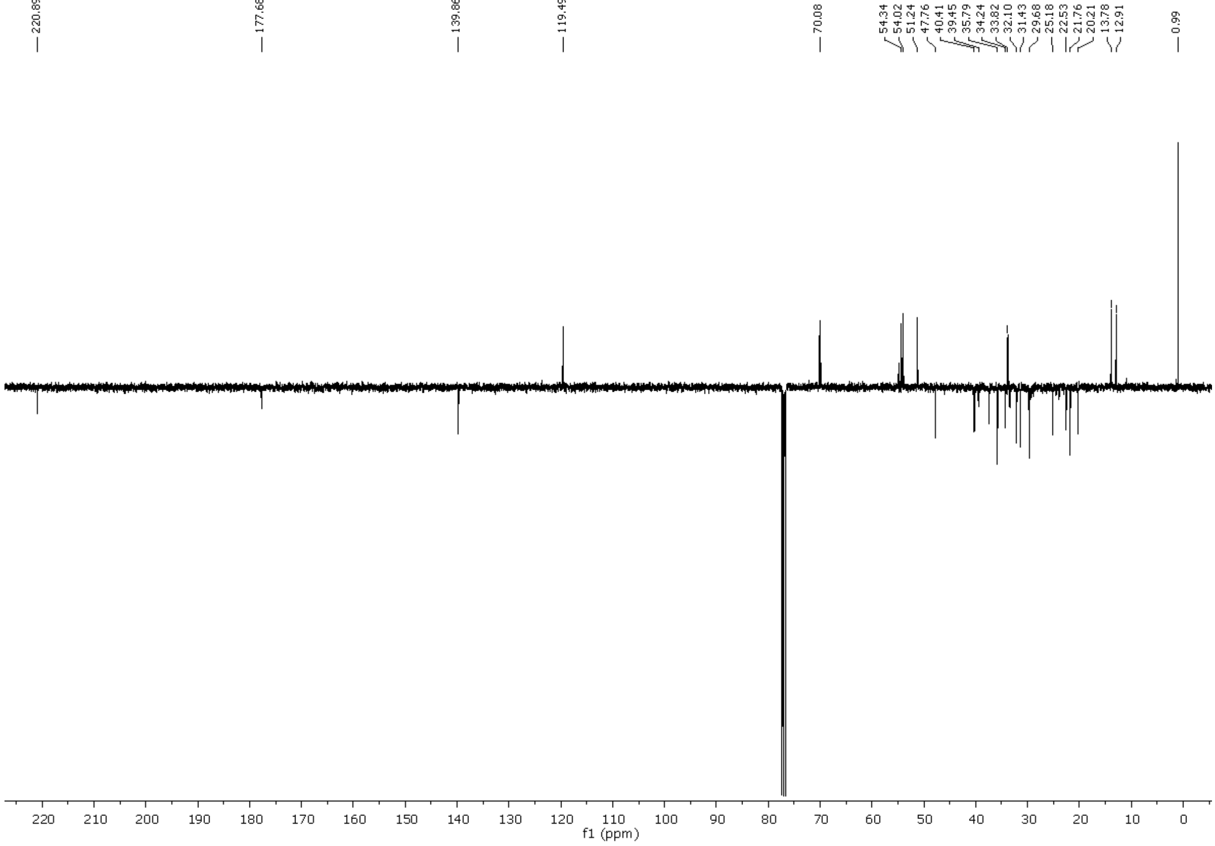

3 ^{1}H and ^{13}C Acetone-*d*6

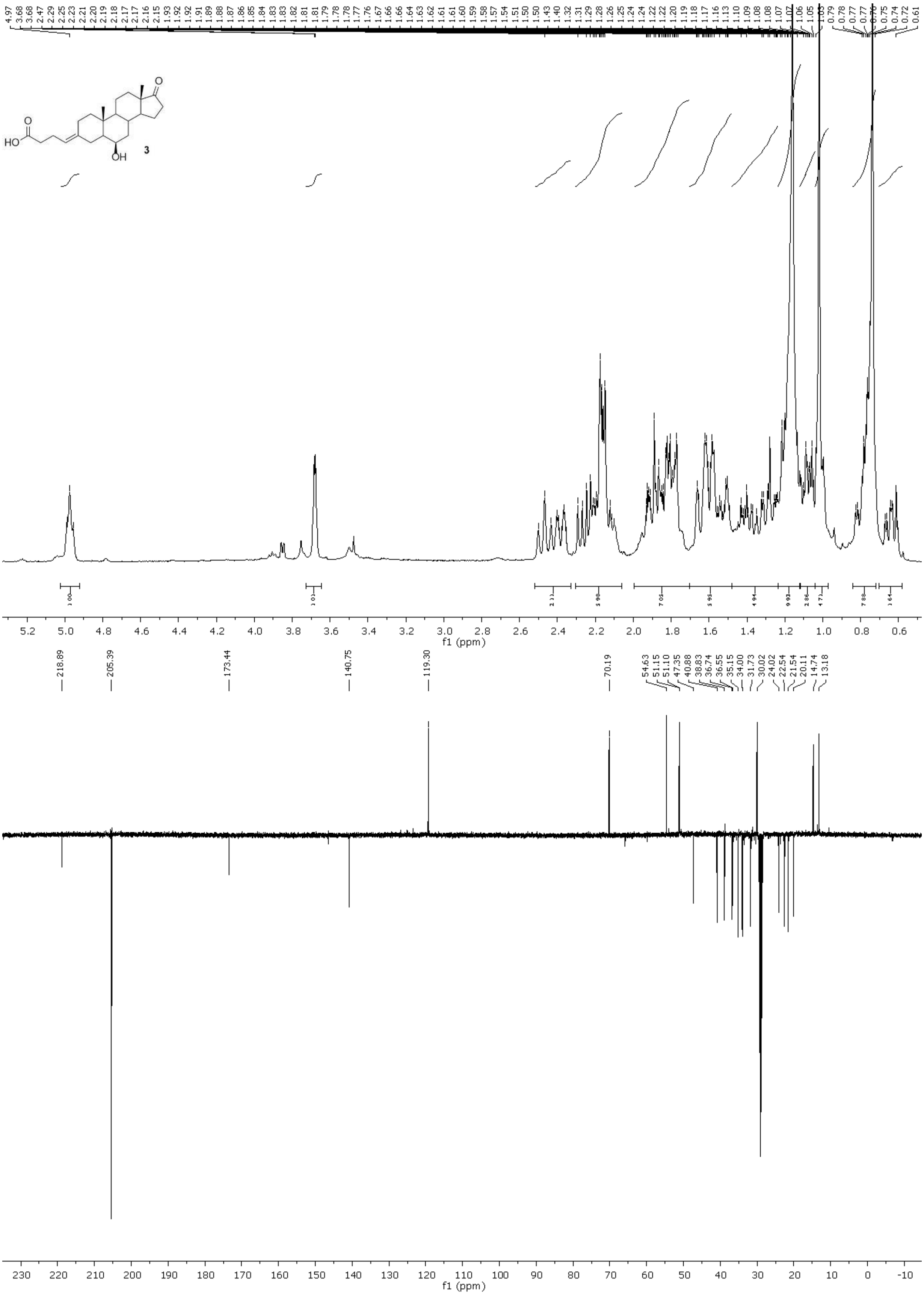

4 ^{1}H and ^{13}C Acetone-*d*6

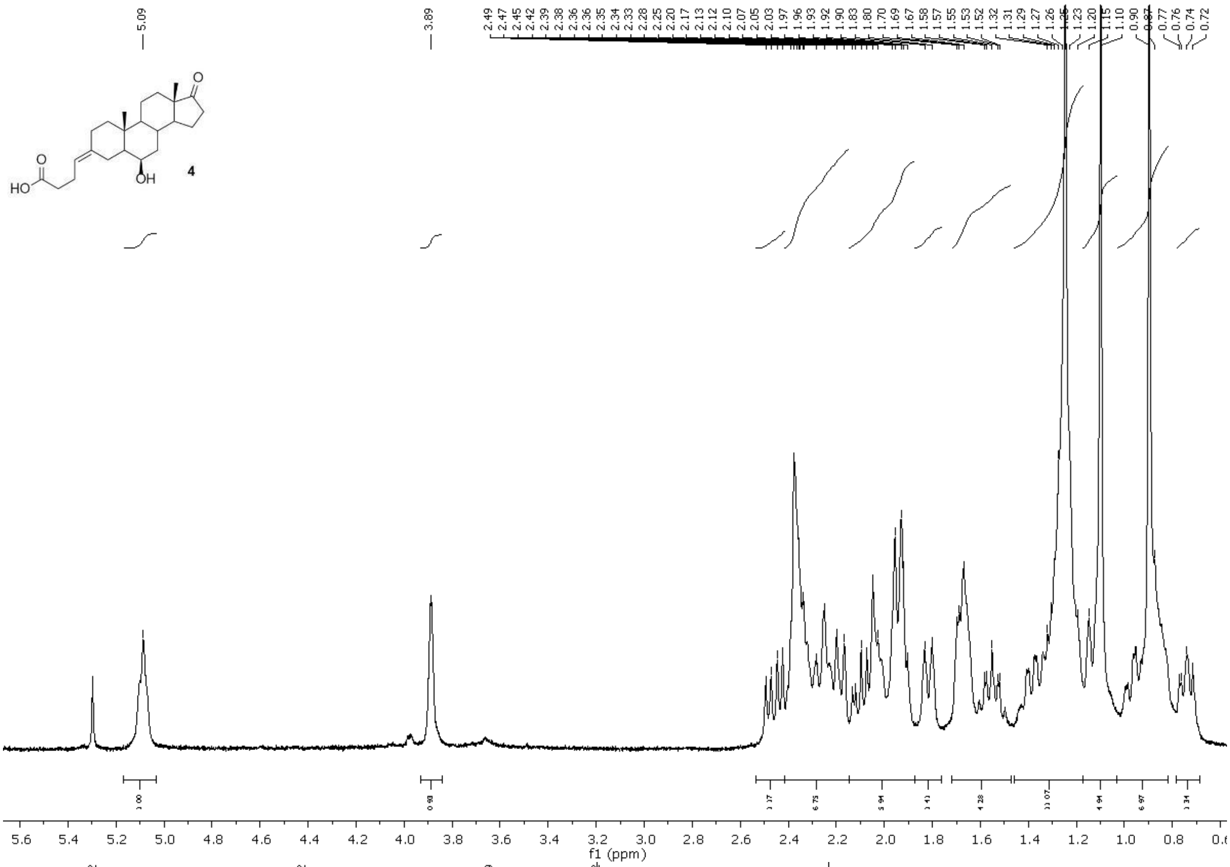

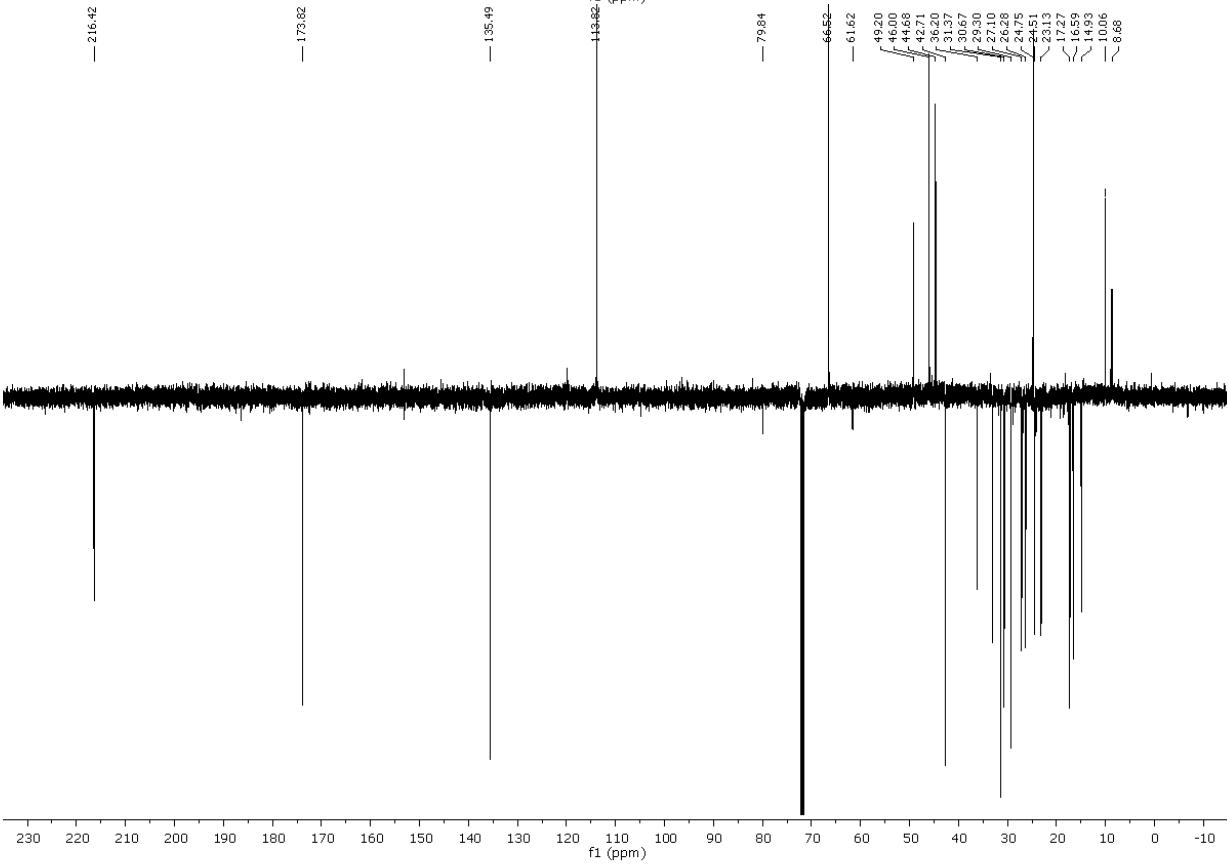

5 ^{1}H and ^{13}C $CDCl_3$

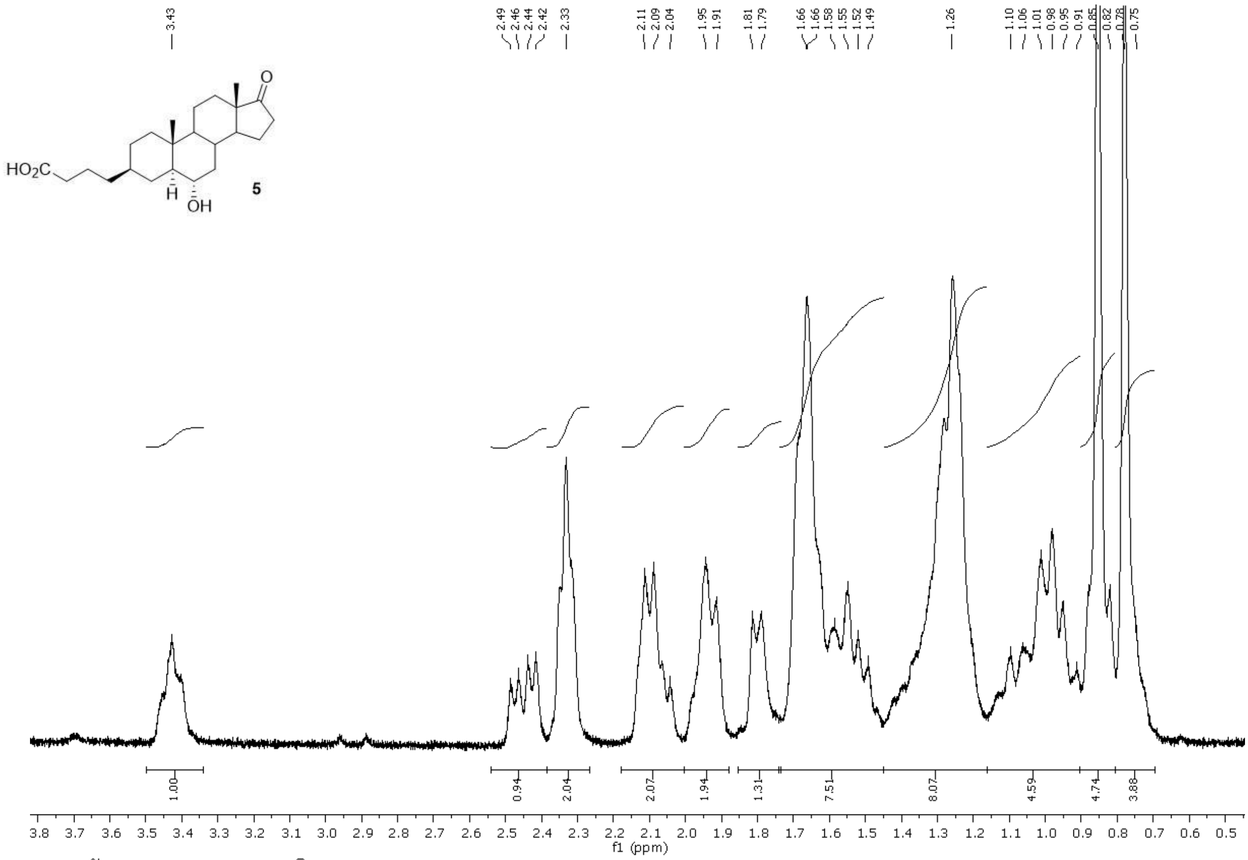

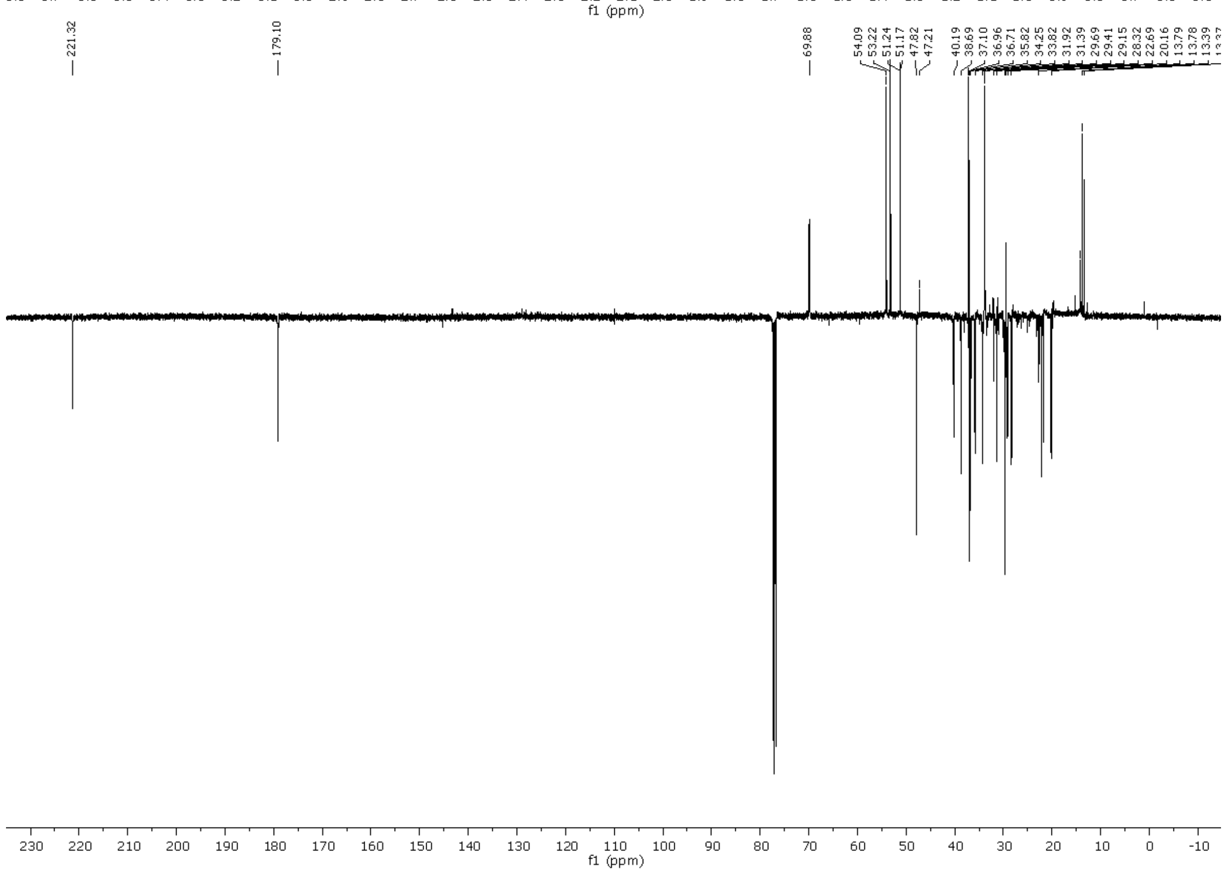

6 ^{1}H and ^{13}C $CDCl_3$

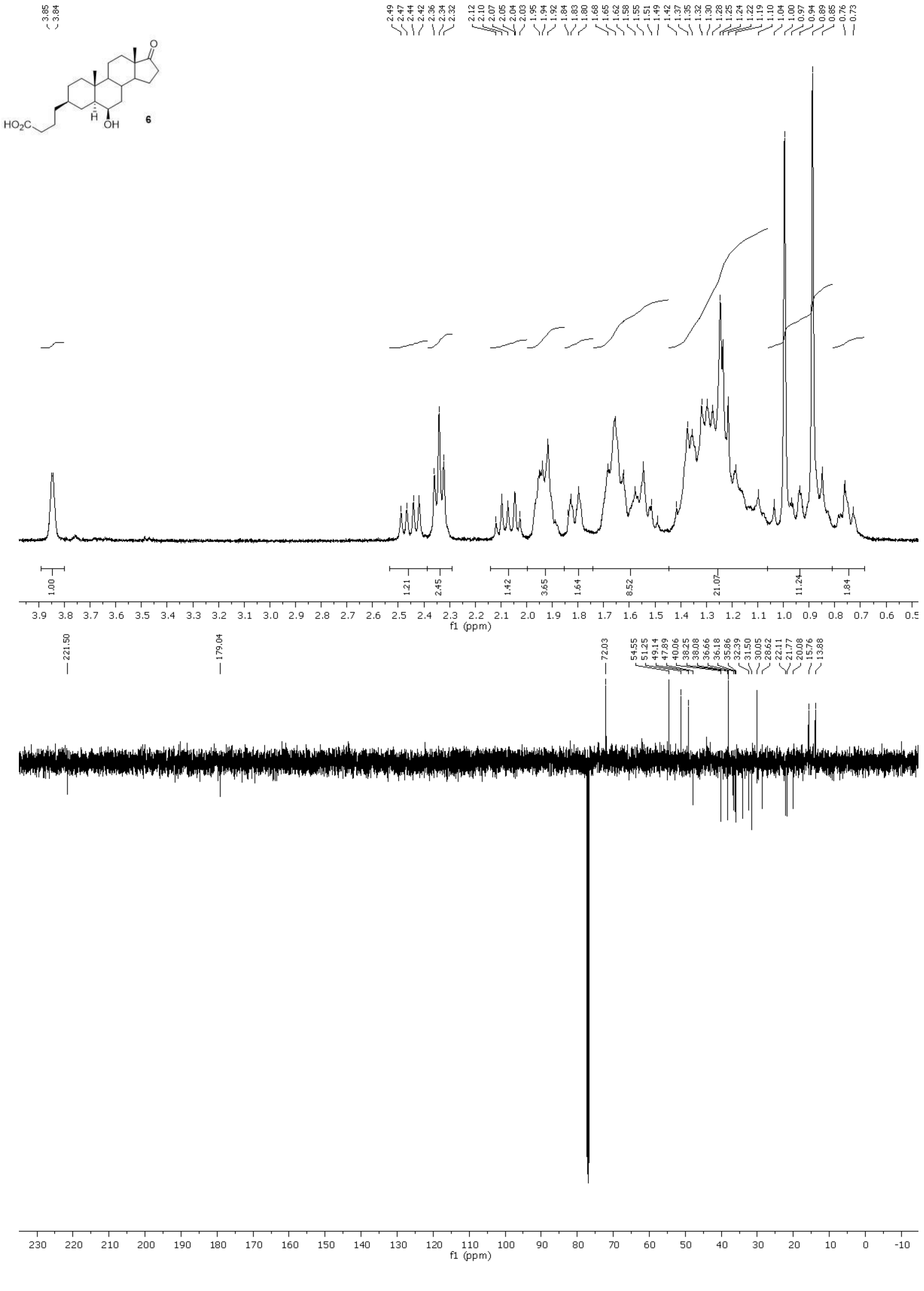

7 ^{1}H and ^{13}C $CDCl_3$

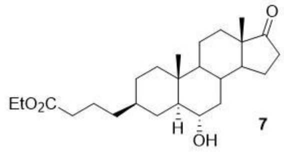

7

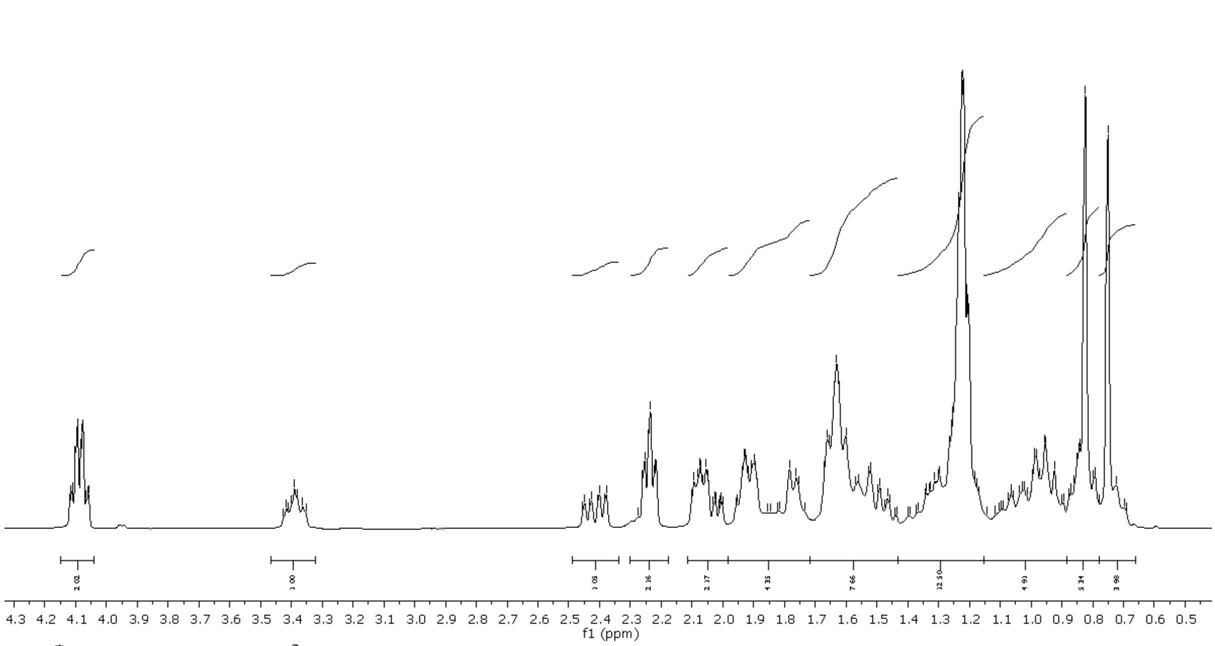

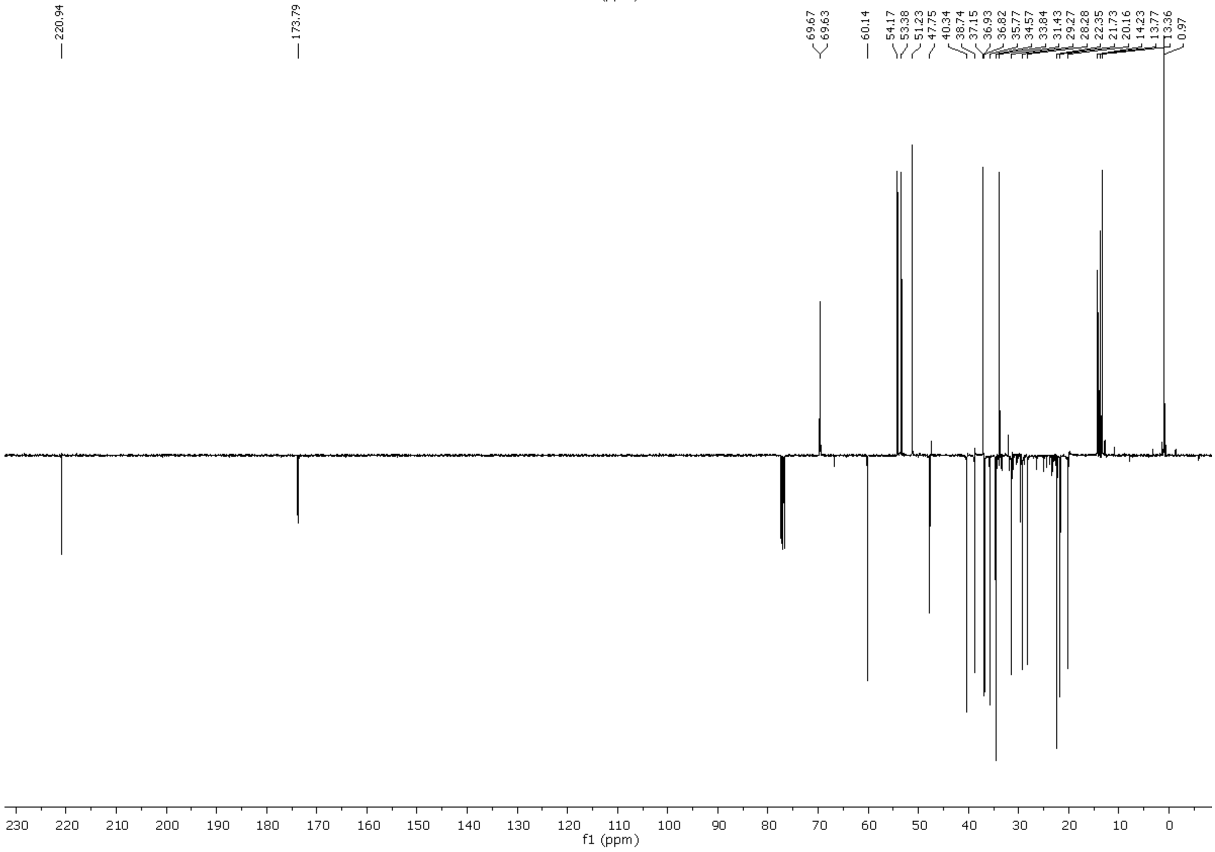

8 ^{1}H $CDCl_3$ and ^{13}C in acetone

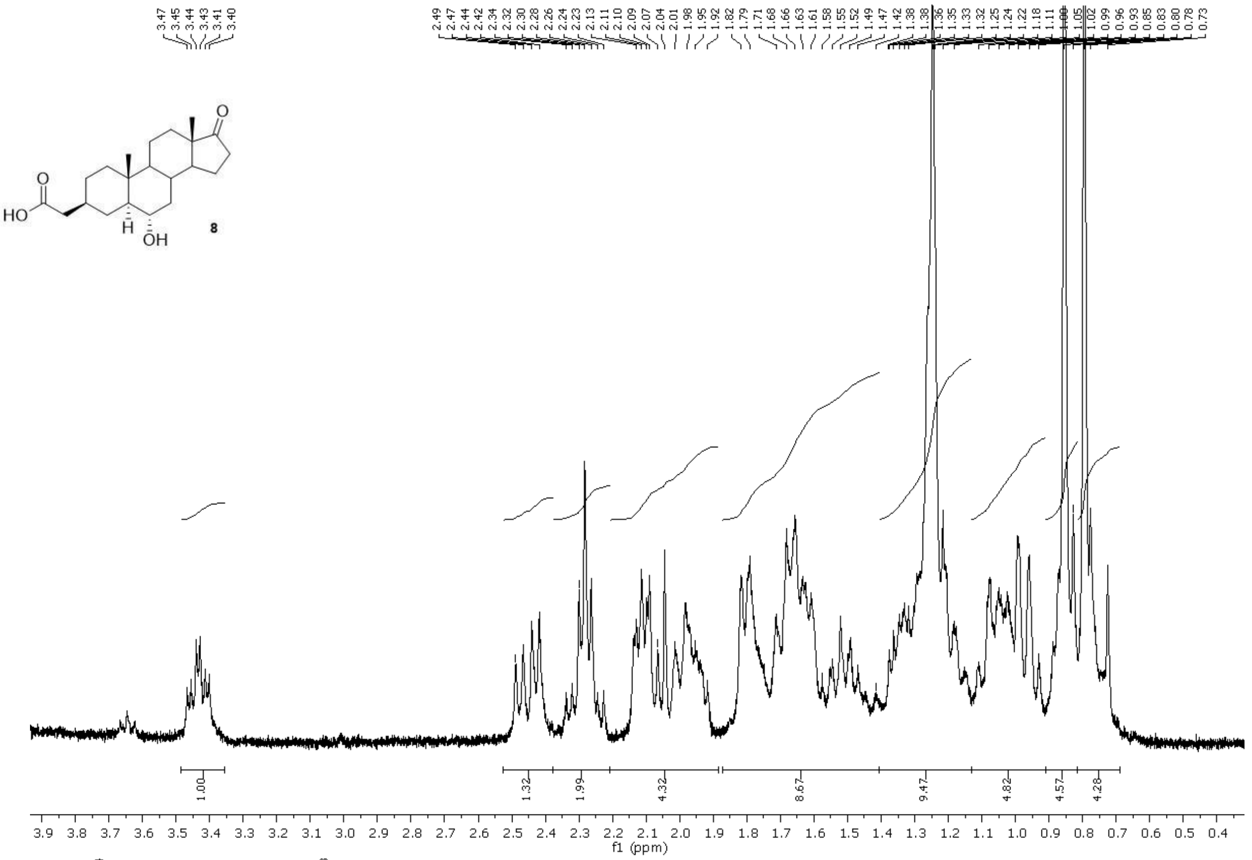

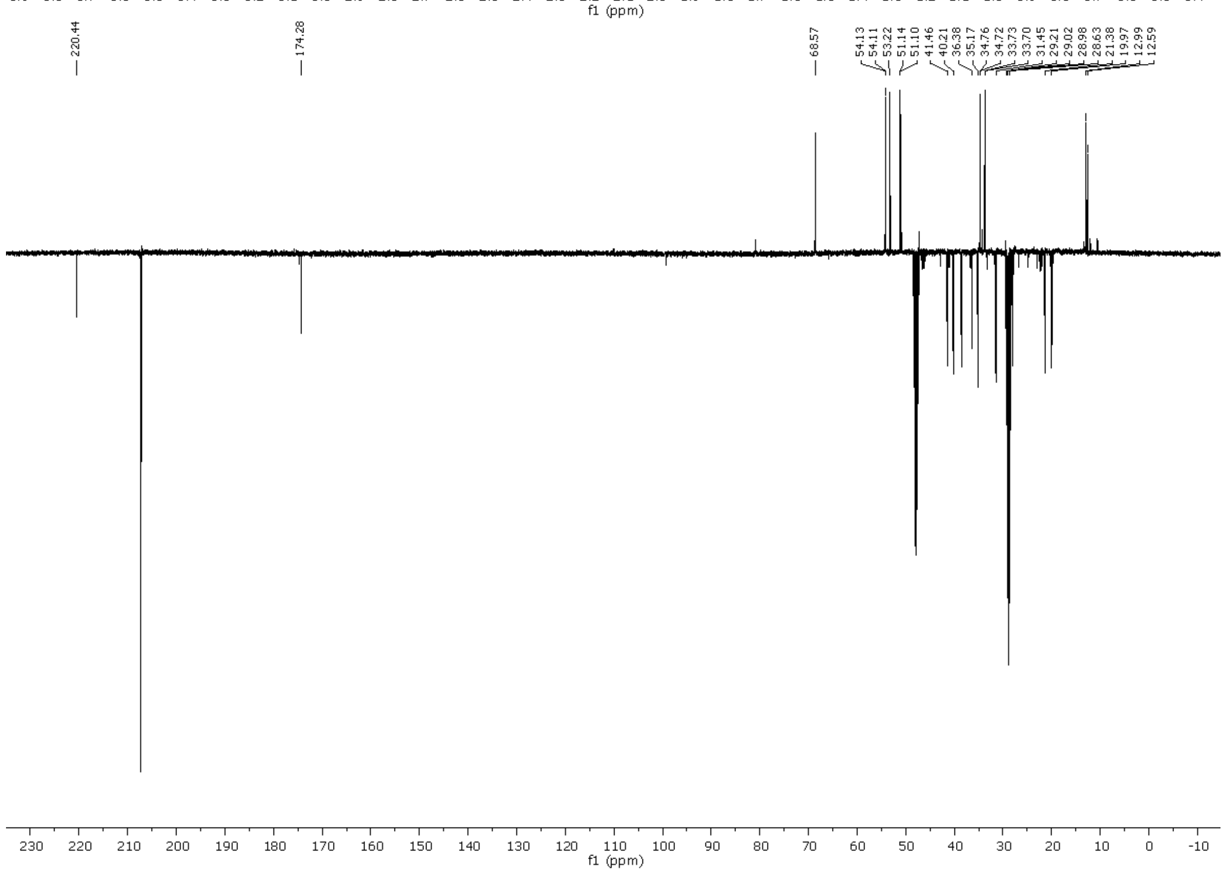

9 ^{1}H and ^{13}C $CDCl_3$

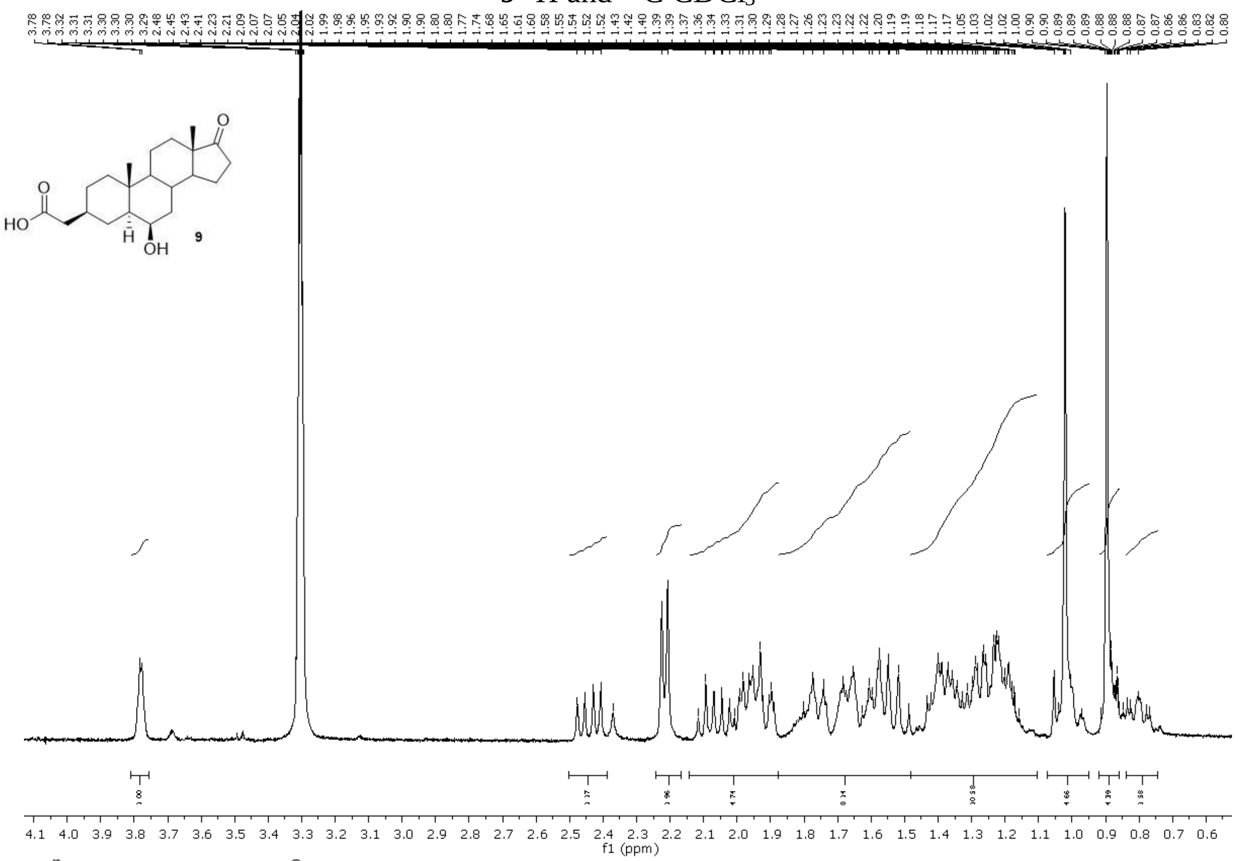

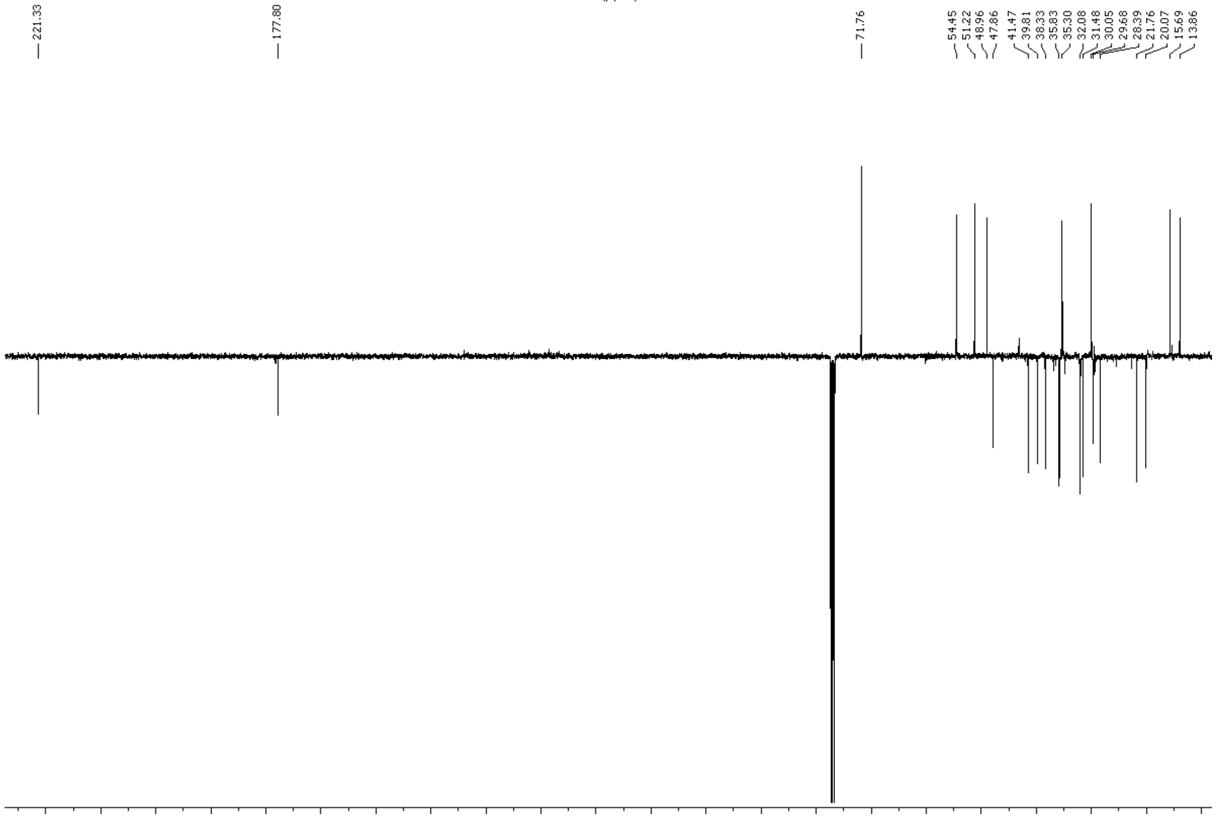

10 ^{1}H and ^{13}C $CDCl_3$

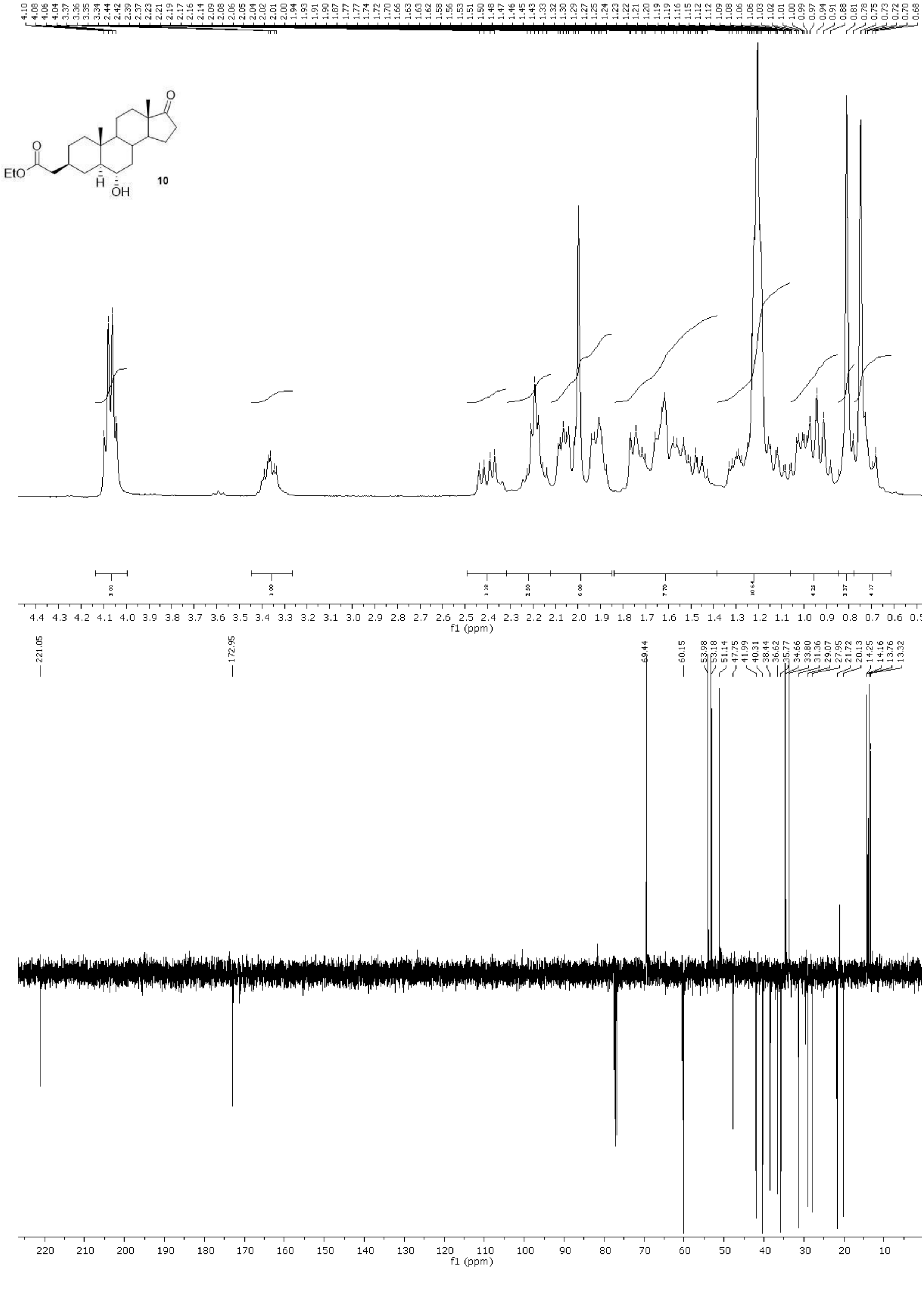

11 ^{1}H and ^{13}C $CDCl_3$

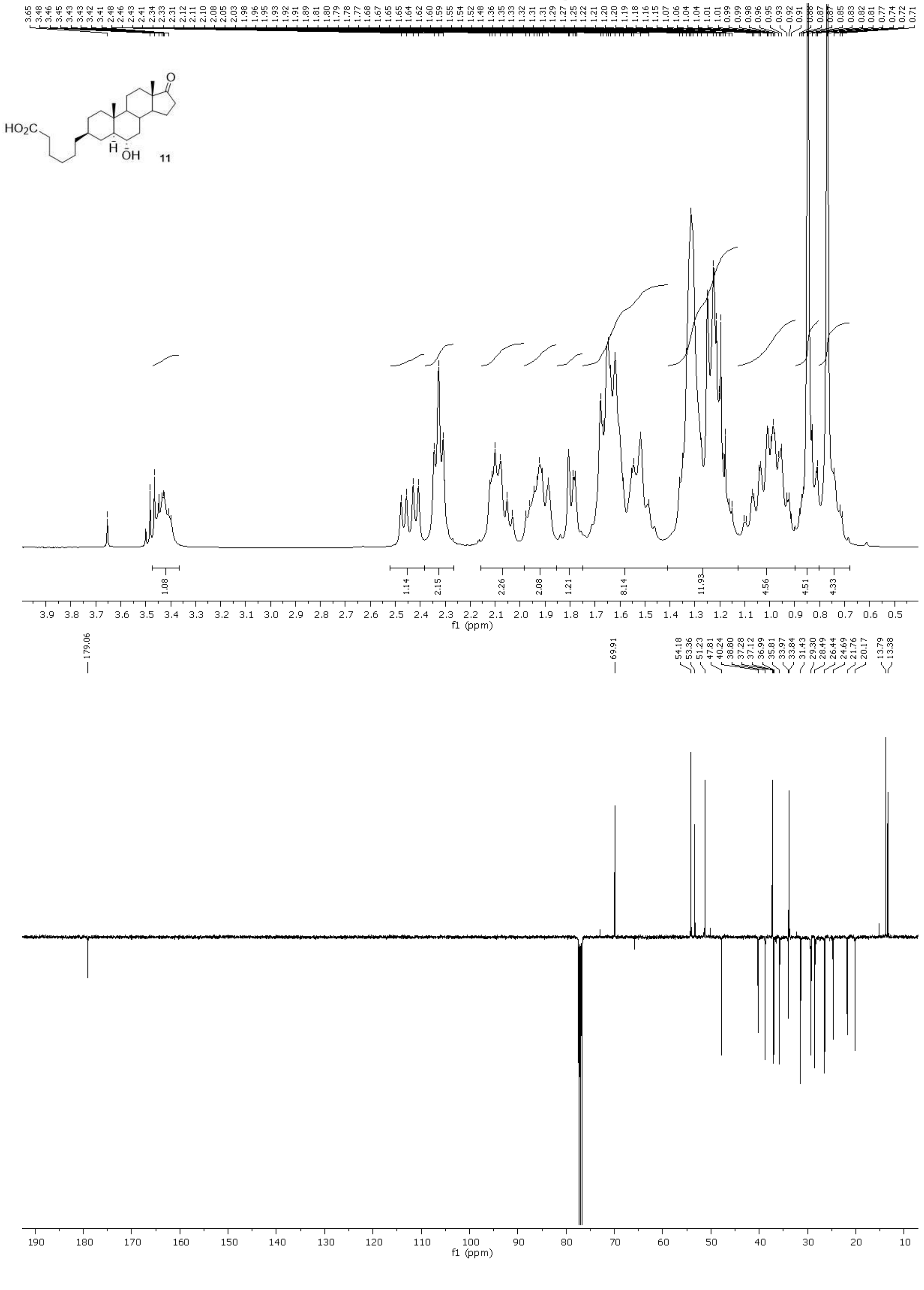

12 ^1H and ^{13}C $CDCl_3$

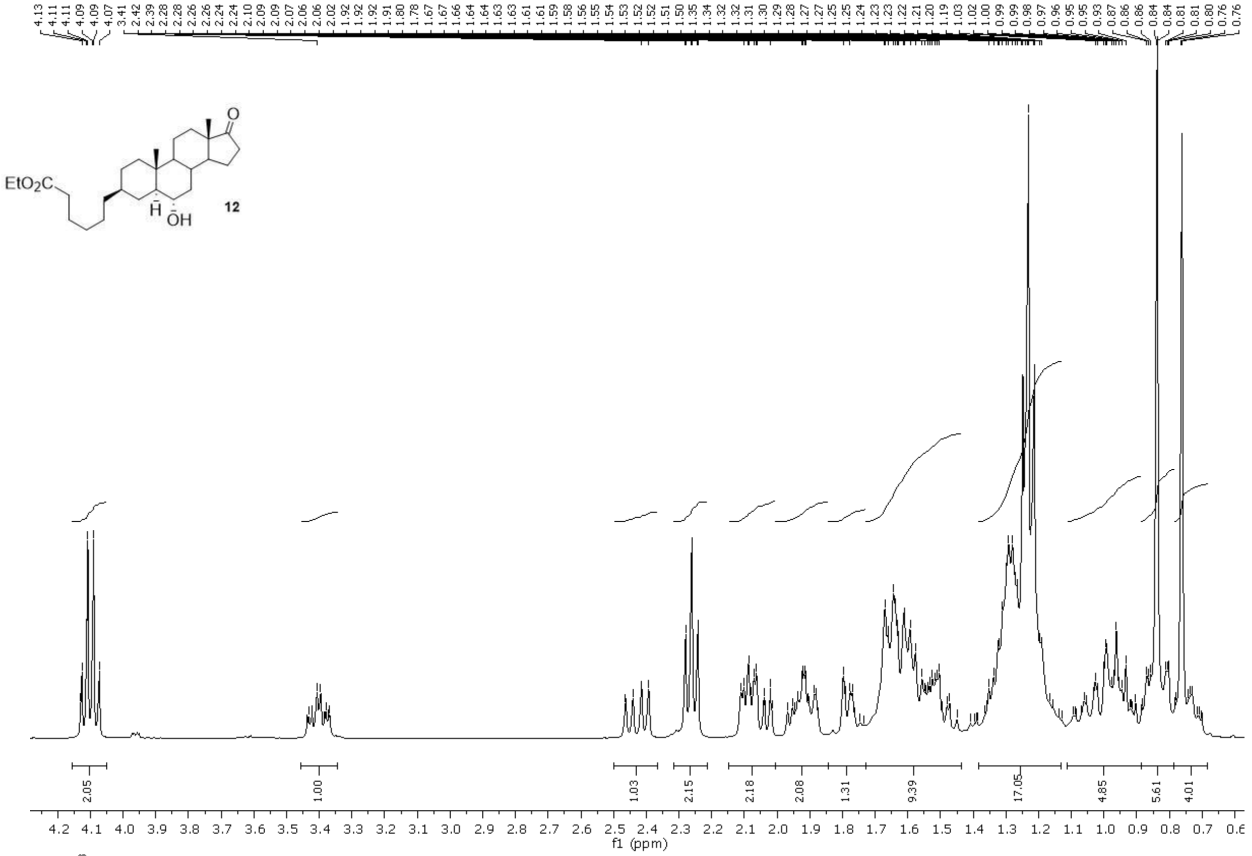

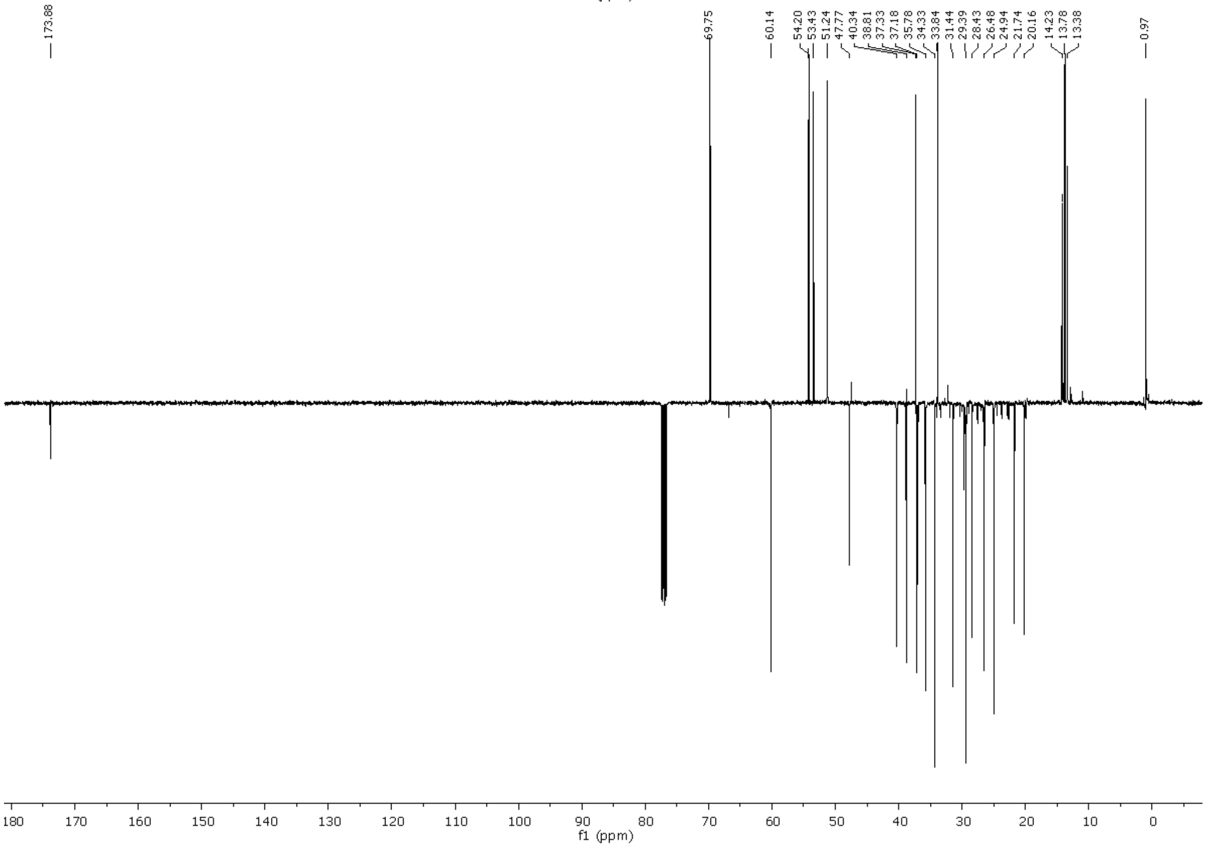

15 ^{1}H $CDCl_3$

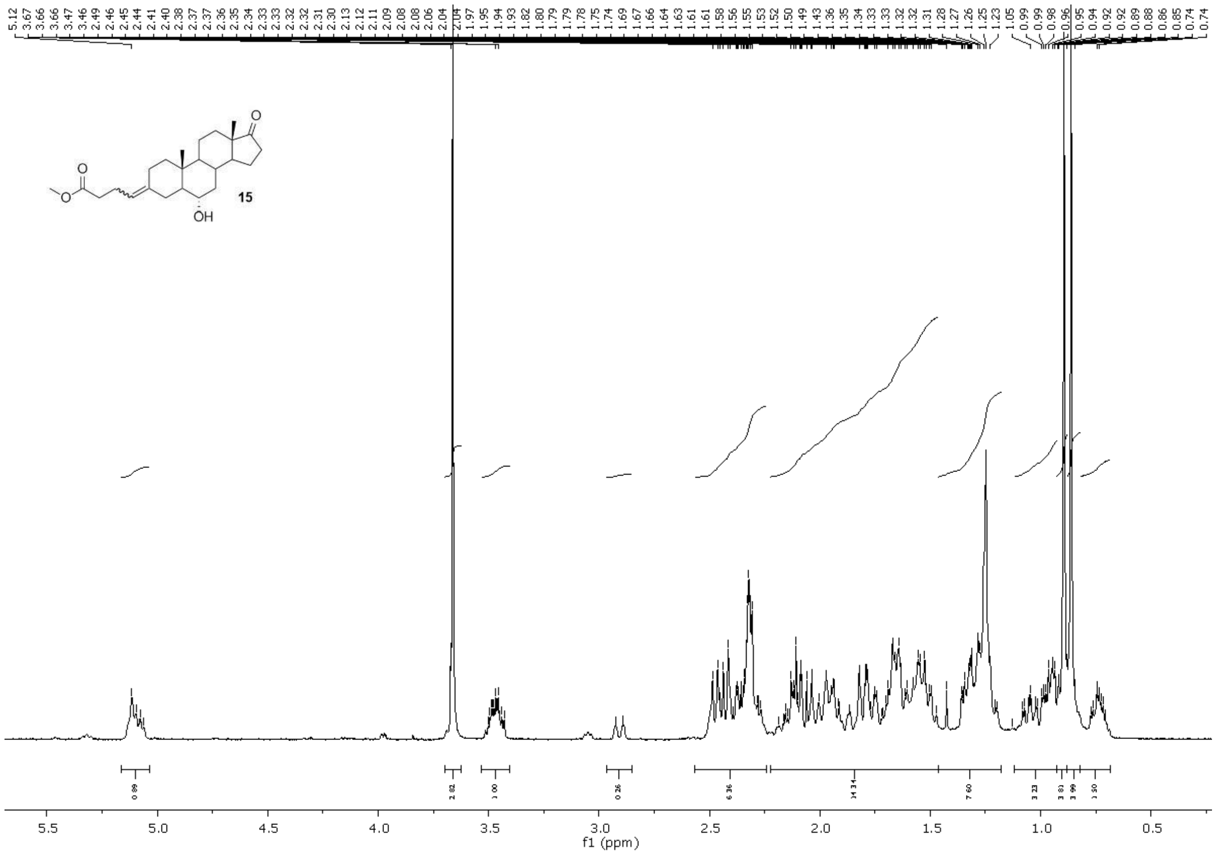

16 ^{1}H and ^{13}C CDCl$_3$

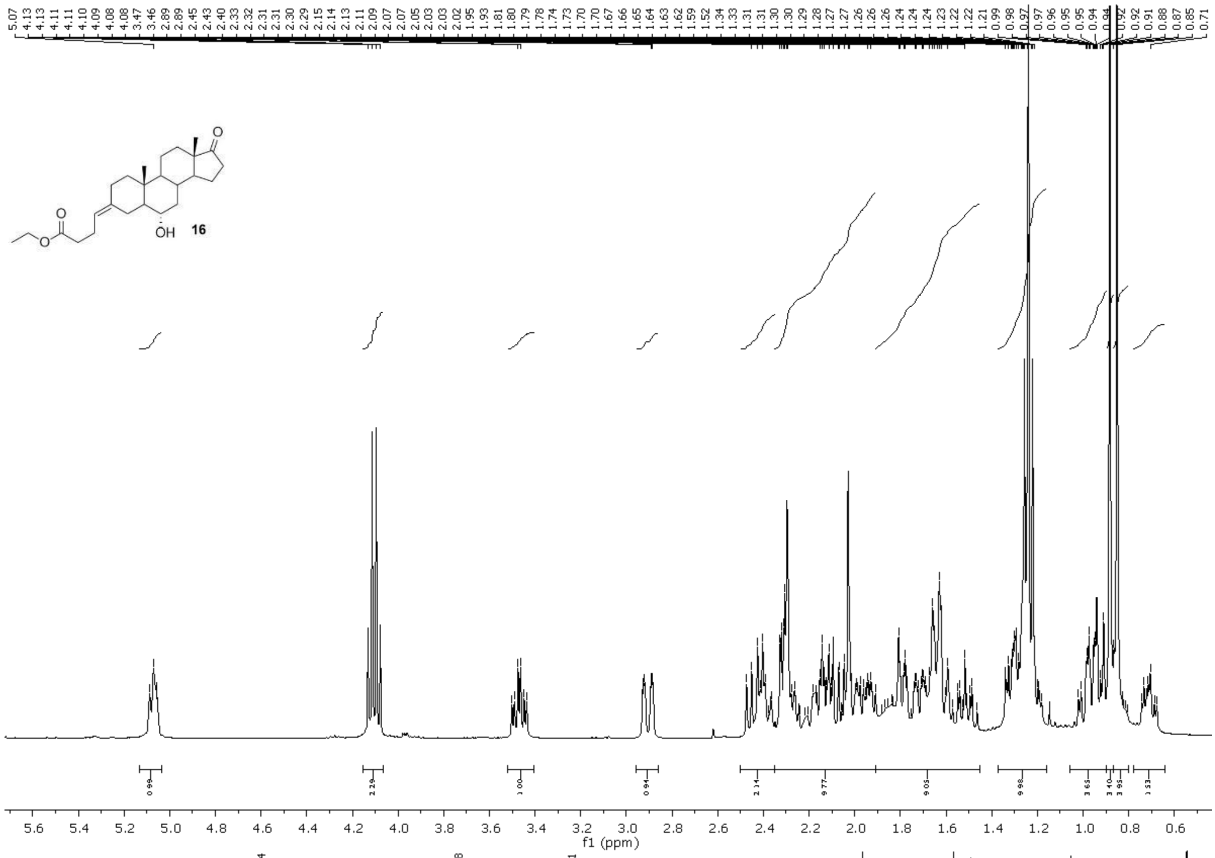

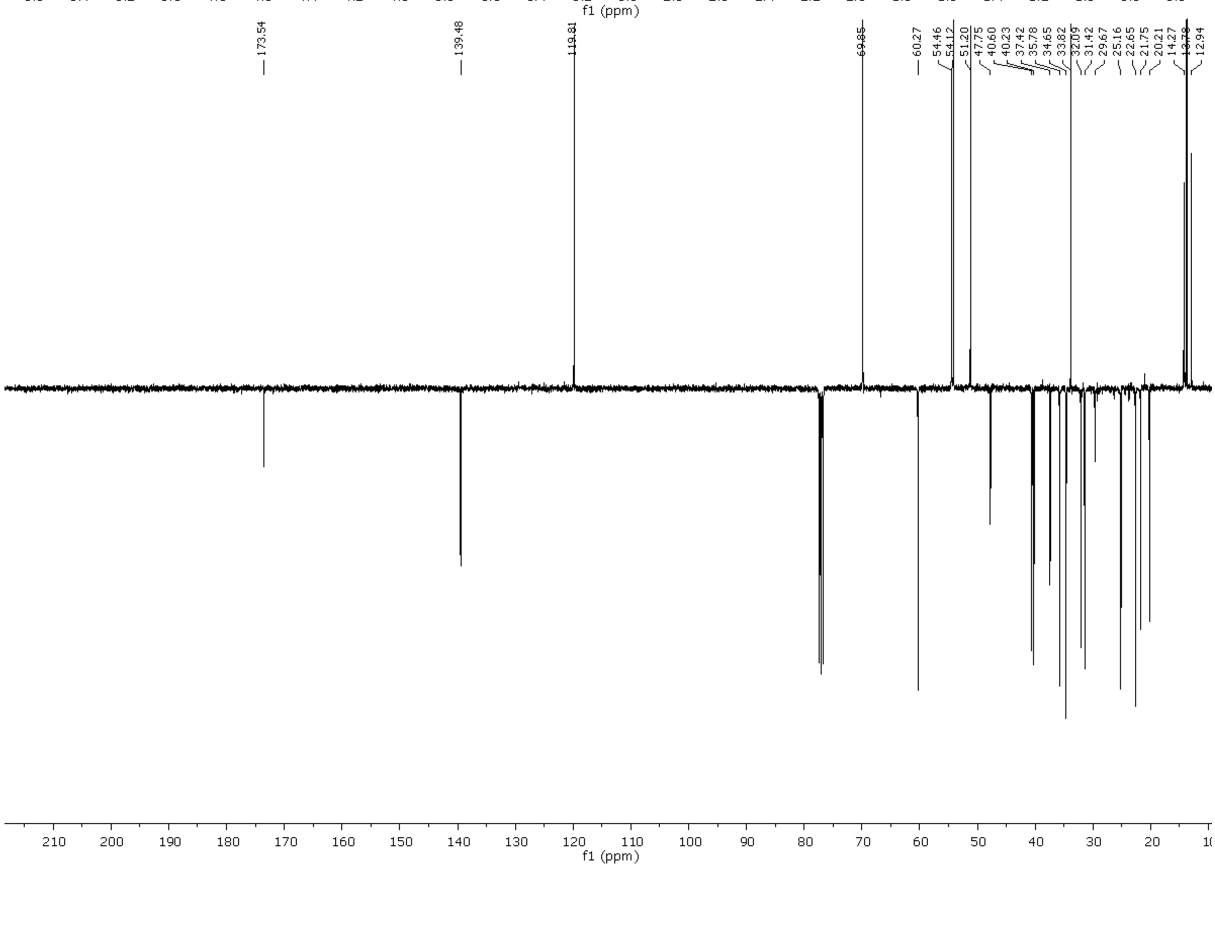

17 ^{1}H and ^{13}C $CDCl_3$

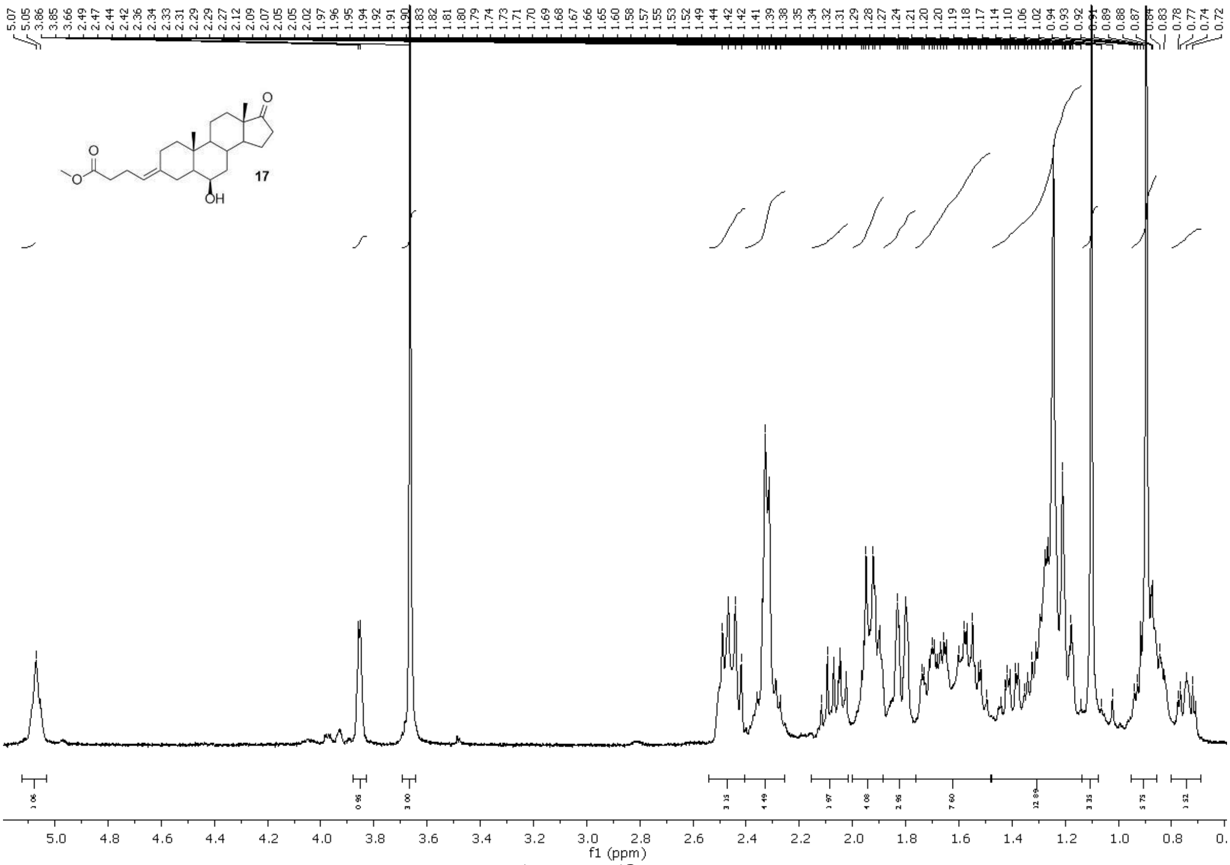

18 ^{1}H and ^{13}C $CDCl_3$

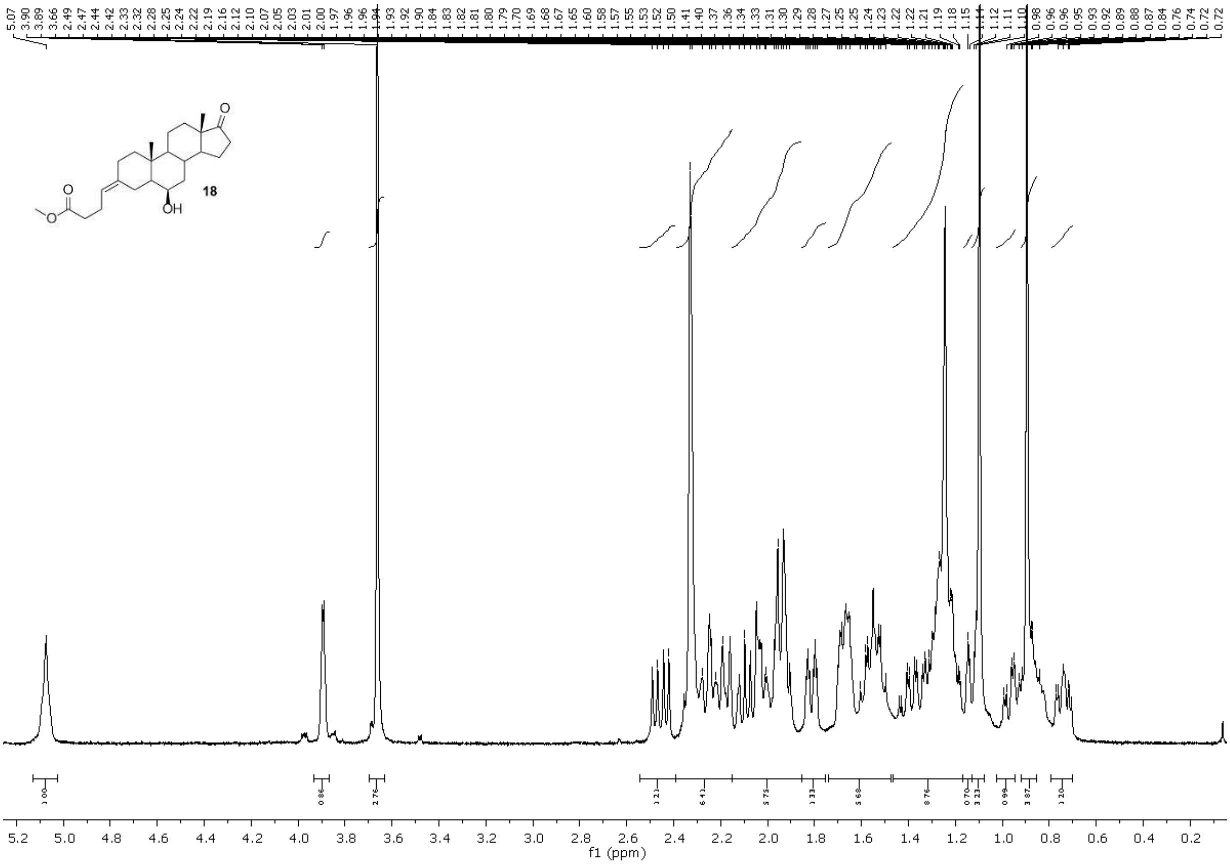

19 ^{1}H and ^{13}C CDCl$_3$

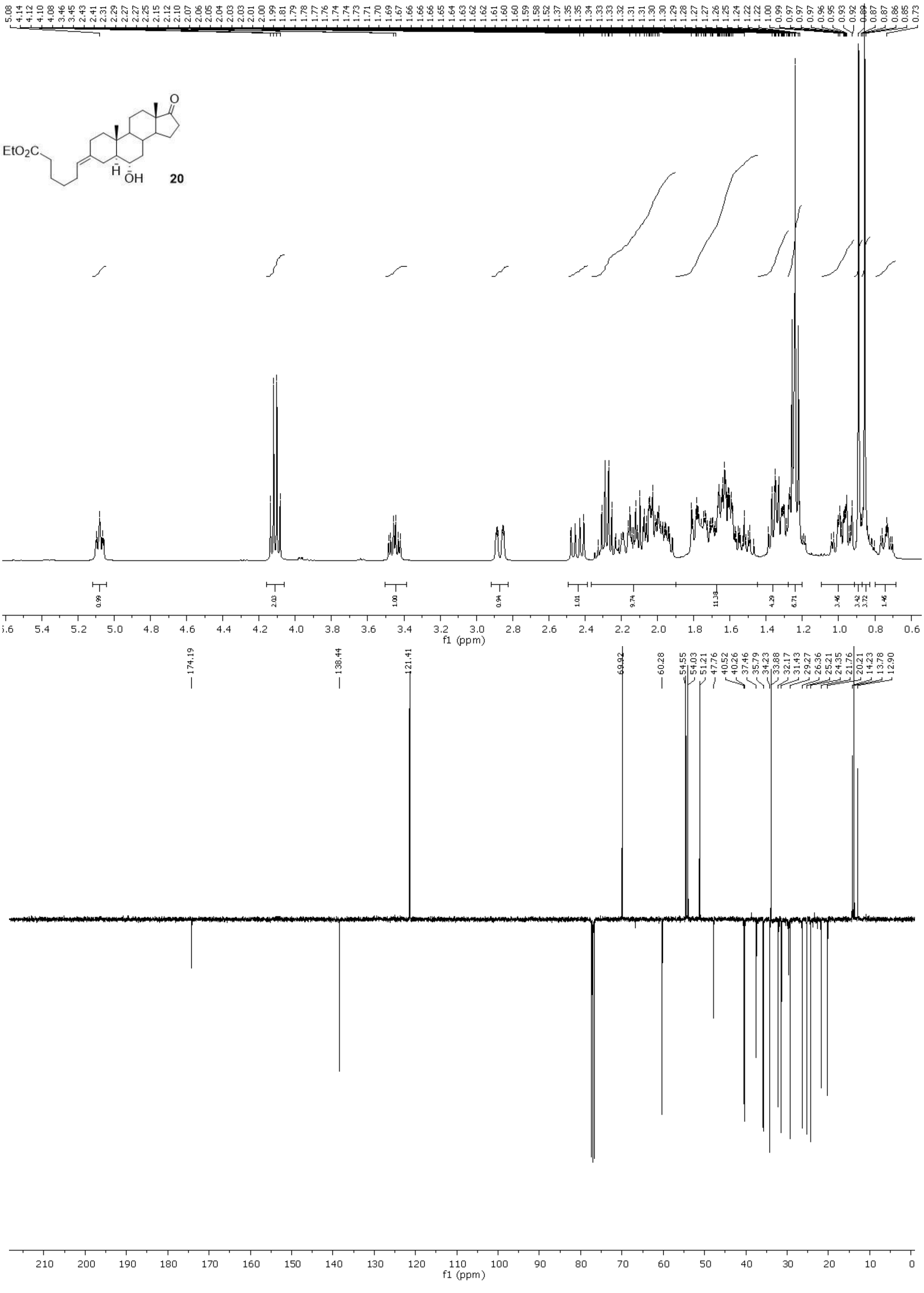

20 ^{1}H and ^{13}C $CDCl_3$

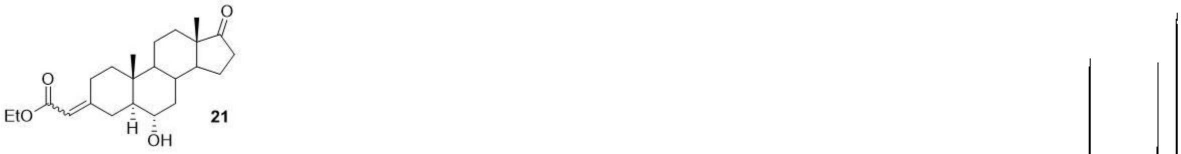

21

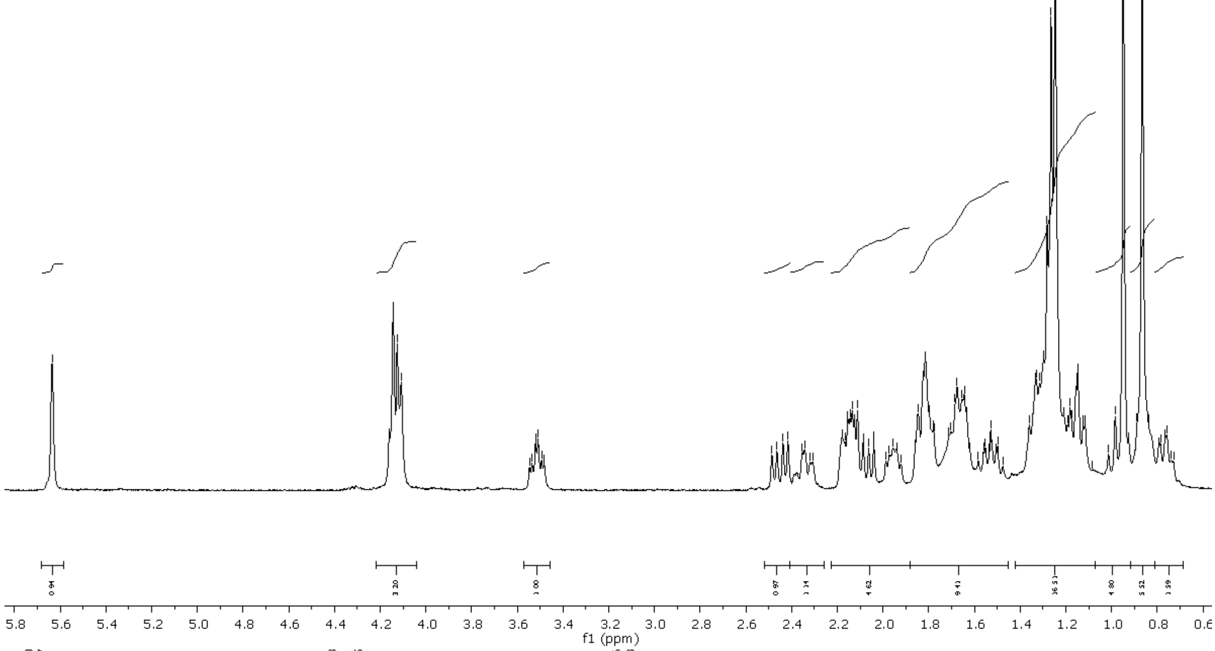

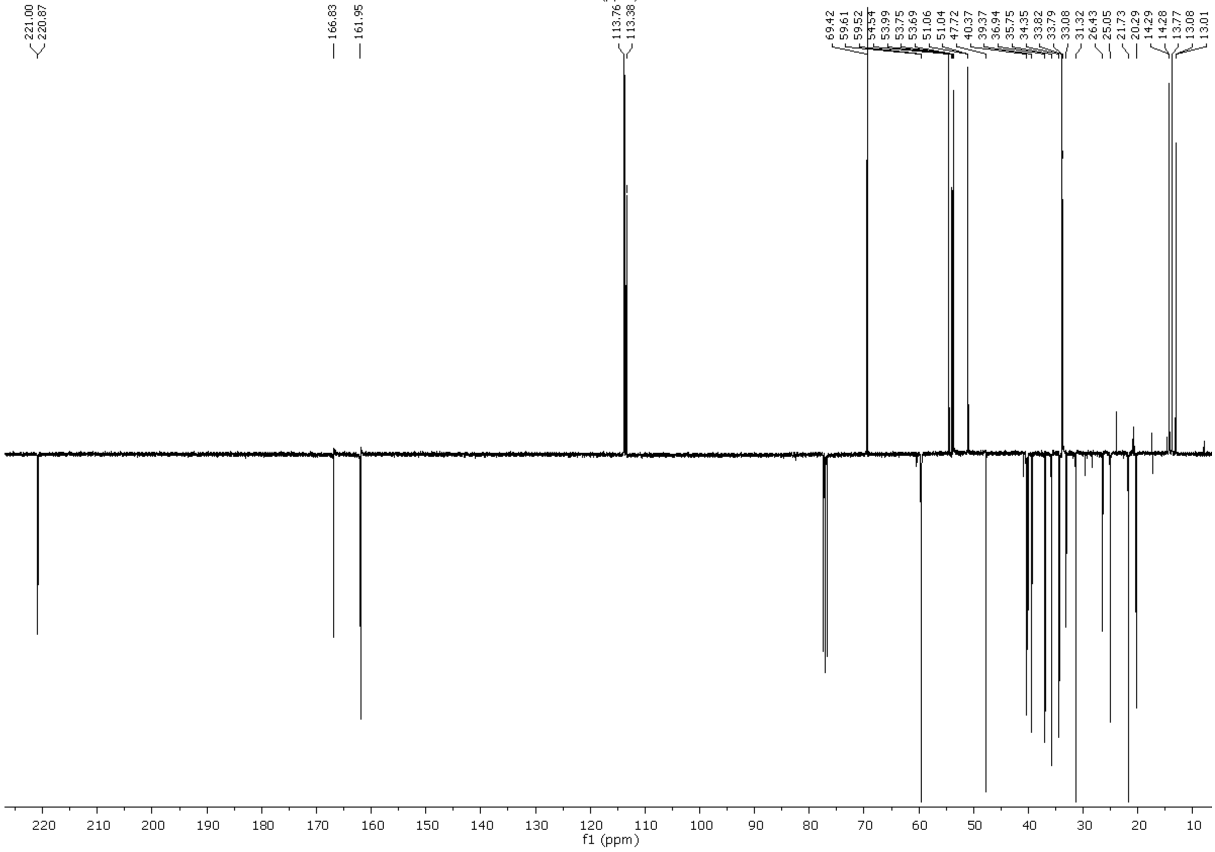

21 ^{1}H and ^{13}C $CDCl_3$

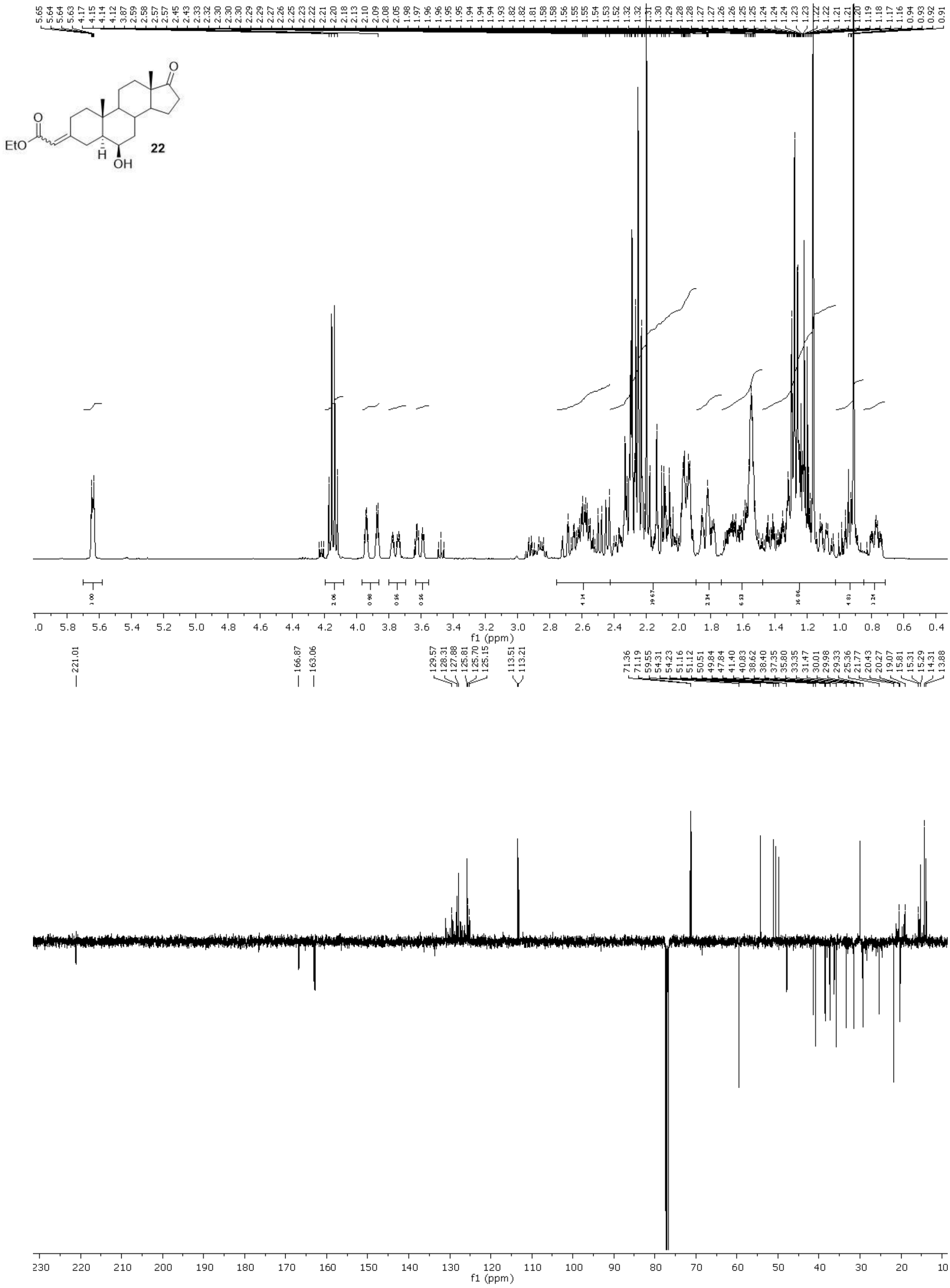

22 ^{1}H and ^{13}C $CDCl_3$

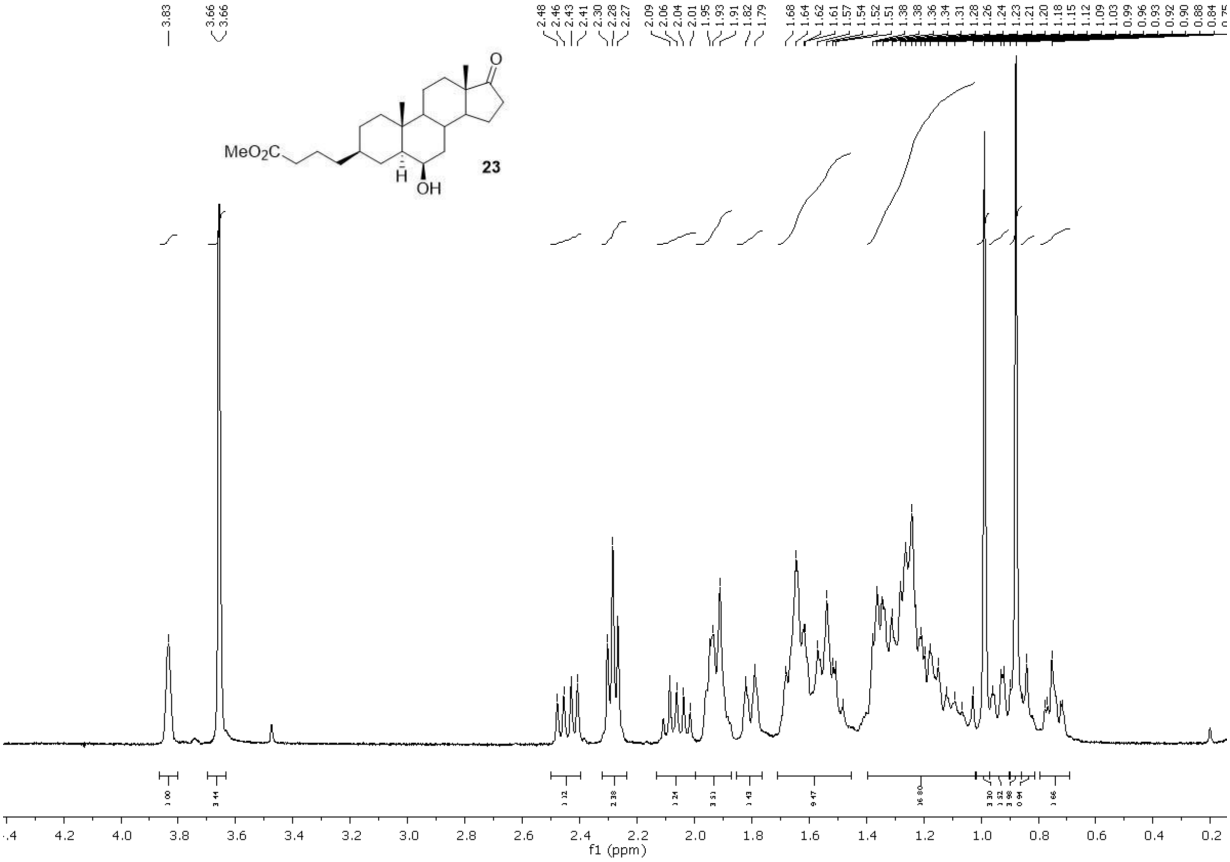

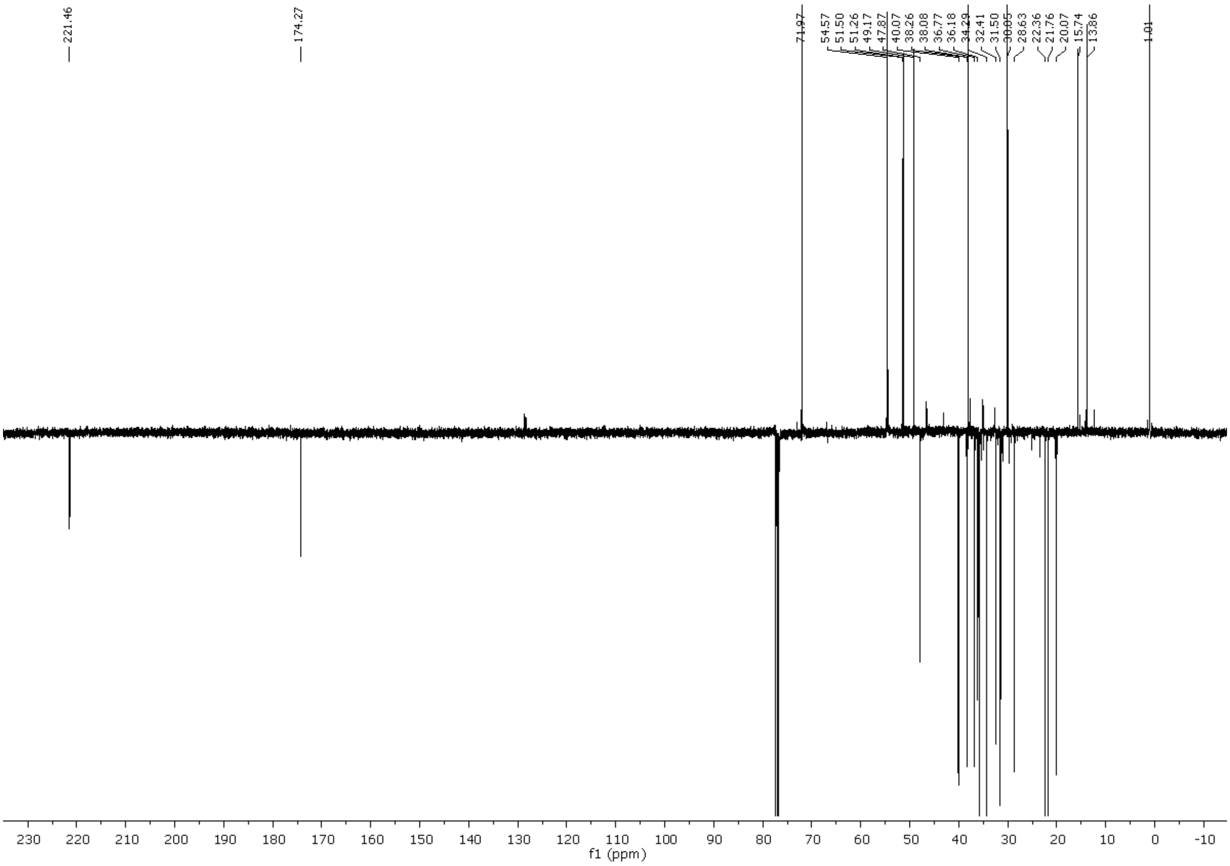

23 ^{1}H and ^{13}C $CDCl_3$

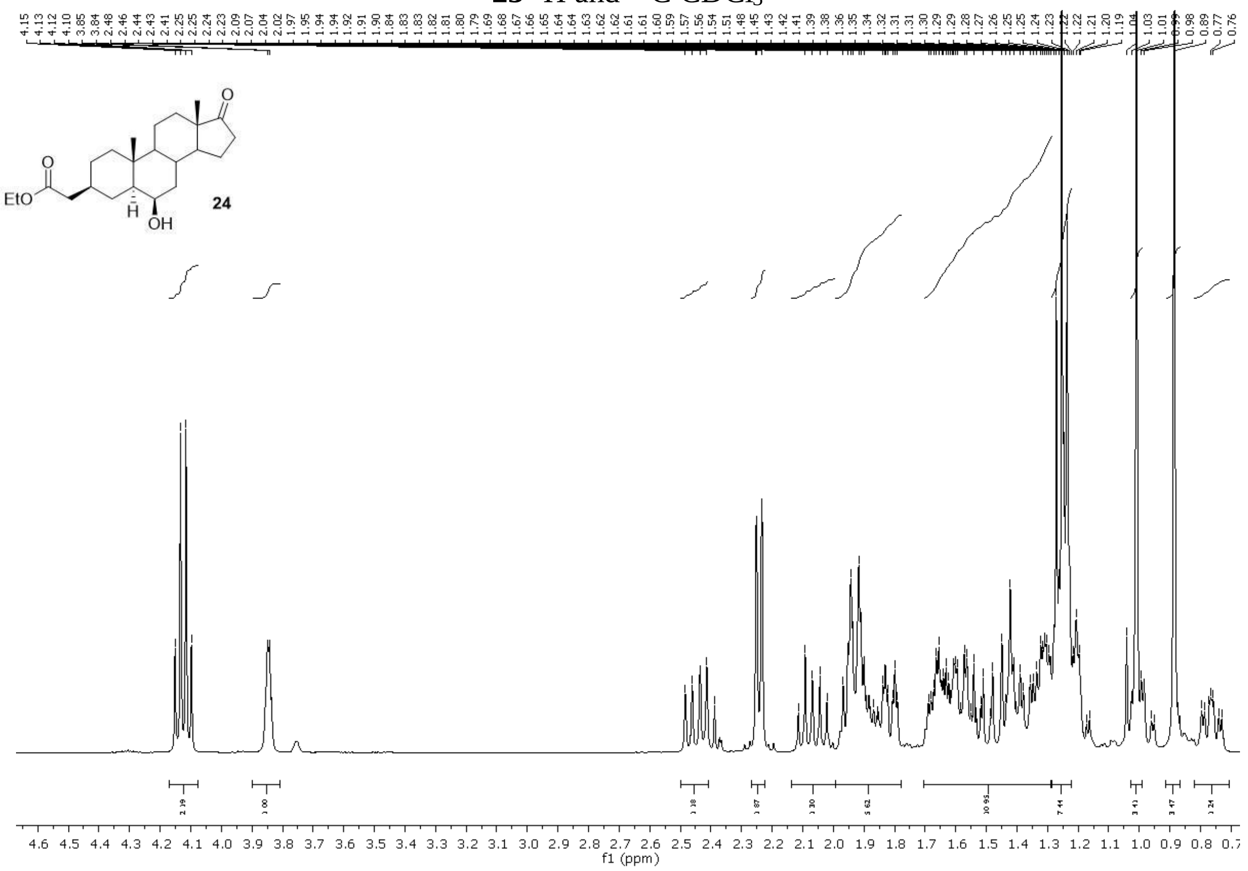

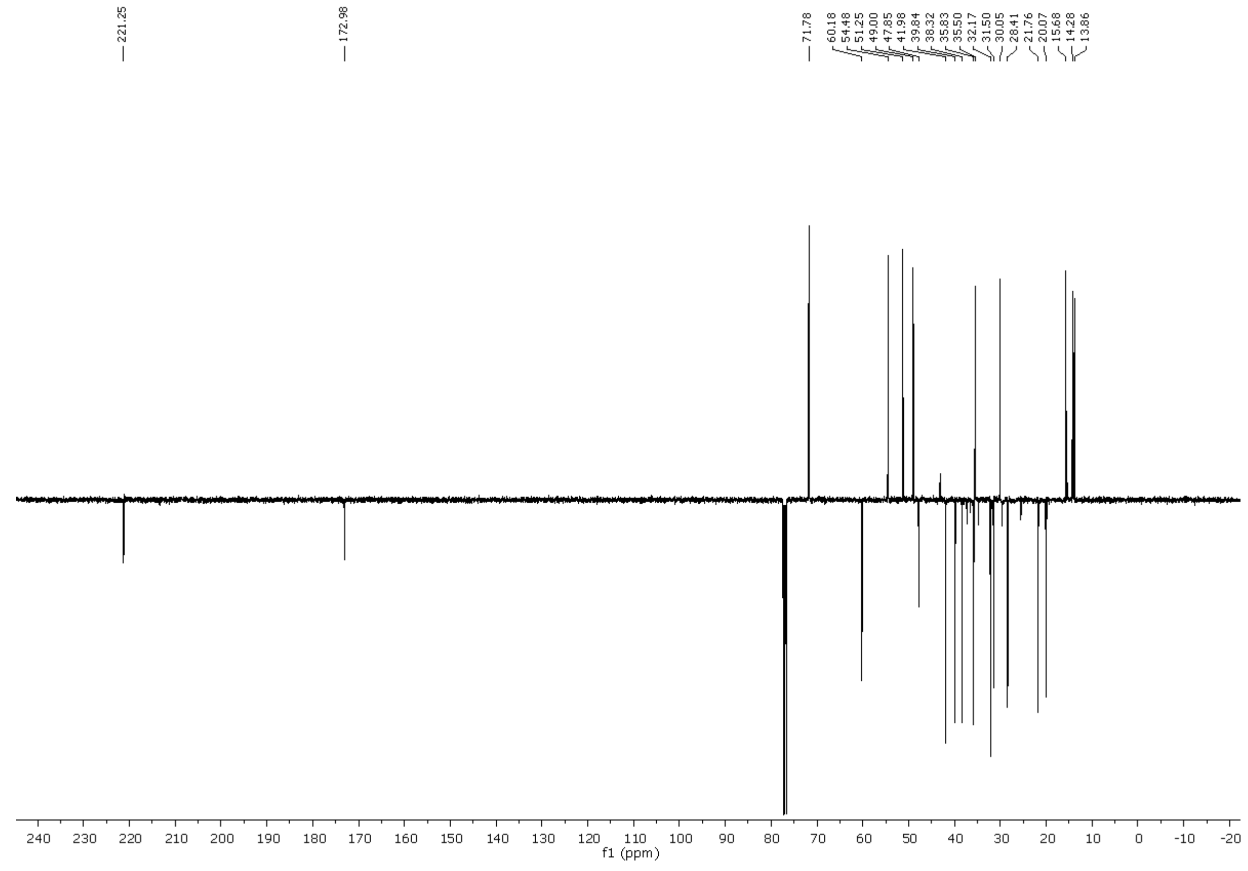

HPLC analysis

General. Column: InfinityLab Poroshell 120 EC- C18; 2.7 µm, 3.0 x 150 mm. Flow rate: 0.8 mL/min. Detection:292 nm.

Mobile phase	Start	End
H_2O+0.01 HCOOH	95%	5%
ACN+0,01 HCOOH	5%	95%

Compound 5

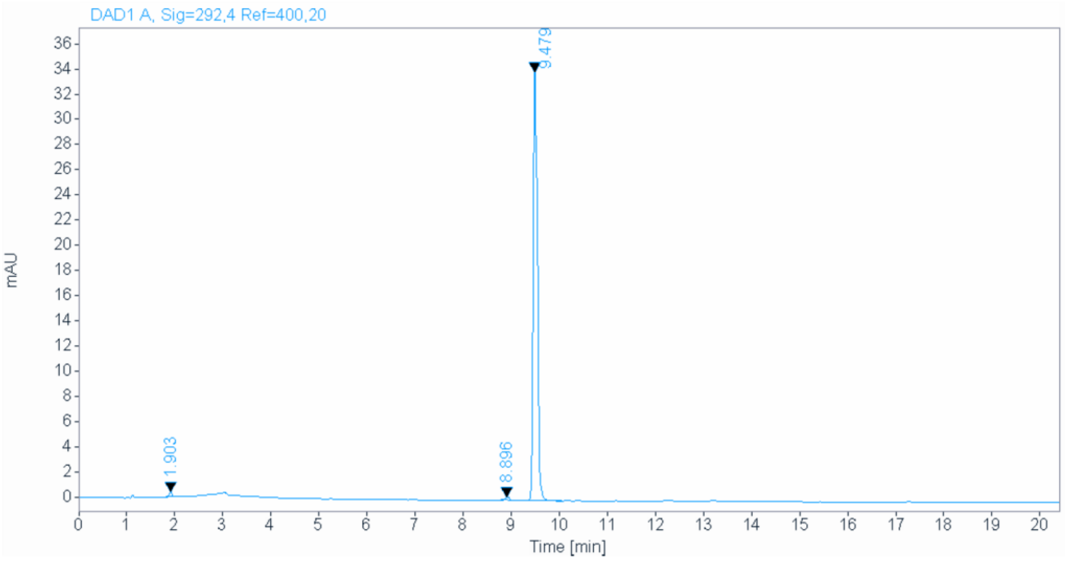

Compound 8

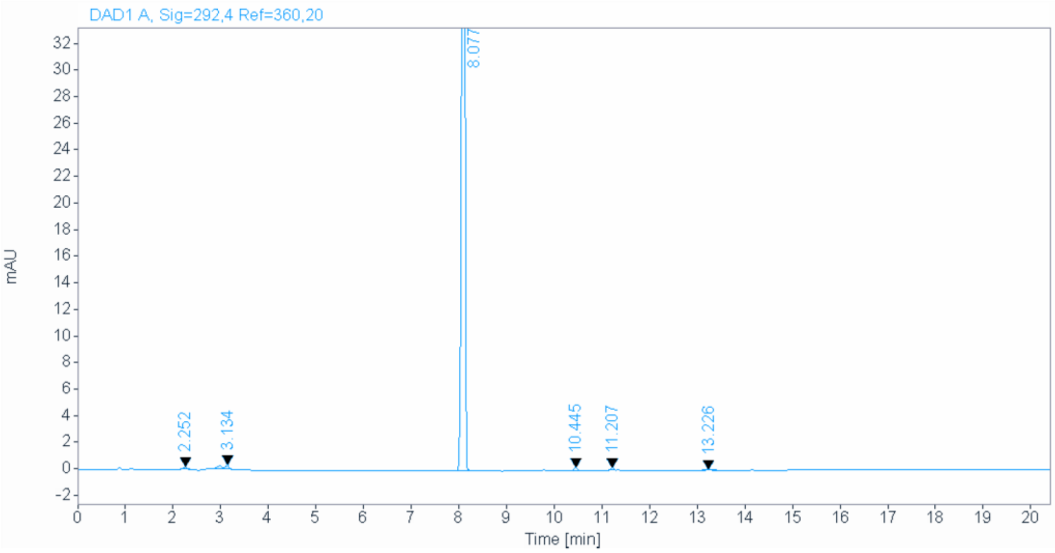

Supplementary methods

Animal models

Male Sprague Dawley rats (150-175 gr) were used to generate a streptozotocin (STZ)-induced diabetic cardiomyopathy model to test compounds *in-vitro* and *in-vivo*; female Dunkin-Hartley guinea-pigs (175-200 g) were used for cardiac and skeletal muscle preparations (male 450-500 g). Male Albino Swiss CD1 mice (30 g) were used for acute *in-vivo* toxicity.

Biochemical measurements

Renal Na^+/K^+ ATPase purification and activity

Purification of renal Na^+/K^+ ATPase was performed according to the method of Jørgensen[2]. Kidneys were excised from 1–3-year-old male beagle dogs, obtained from the General Pharmacology Department of Sigma-tau, Pomezia, Italy, under pentobarbital anesthesia. Kidneys were sliced and the outer medulla was dissected, pooled and suspended (1g in 10 ml) in a sucrose-histidine solution, containing 250 mM sucrose, 30 mM histidine and 5 mM EDTA, pH 7.2 and homogenized. The homogenate was centrifuged at 6,000 g for 15 min, the supernatant was decanted and centrifuged at 48,000 g for 30 min. The pellet was suspended in the sucrose-histidine buffer and incubated for 20 min with a sodium-dodecyl-sulphate (SDS) solution, dissolved in a gradient buffer, containing 25 mM imidazole and 1 mM EDTA, pH 7.5. The sample was layered on the top of a sucrose discontinuous gradient (10, 15 and 29.4%) and centrifuged at 60,000 g for 115 min. The final pellet was suspended in the gradient buffer.

Na^+/K^+ ATPase activity was assayed by measuring ^{32}P-ATP hydrolysis, as previously described[3]. Increasing concentrations of the standard ouabain, or tested compound, were incubated with 0.3 μg of purified dog kidney enzyme for 10 min at 37°C in 120 μl final volume of a medium, containing 140 mM NaCl, 3 mM $MgCl_2$, 50 mM Hepes-Tris, 3 mM ATP, pH 7.5. Then, 10 μl of incubation solution containing 10 mM KCl and 20 nCi of ^{32}P-ATP (3-10 Ci/mmol, Perkin Elmer) were added, the reaction continued for 15 min at 37°C and was stopped by acidification with 20% ice-cold perchloric acid.

^{32}P was separated by centrifugation with activated Charcoal (Norit A, Serva) and the radioactivity was measured. Effects of increasing concentrations of the tested compound were compared to ouabain, as positive standard, and to vehicle (control) at 37°C. The inhibitory activity was expressed as percent of activity in control. The concentration of compound causing 50% inhibition of the Na^+/K^+ ATPase activity (IC_{50}) was calculated by non-linear fitting of data points (KaleidagraphTM, Sinergy Software).

SERCA ATPase isolation and activity assay

To preserve interaction with proteins of the macromolecular complex within the native membrane lipid environment, SERCA activity was measured in cardiac SR microsomes (guinea-pigs) or homogenates

(rats). As verified by Western blot, cardiac microsomes preparations included SERCA2a and PLN and skeletal muscle ones included SERCA1 only. To confirm the involvement of PLN in compound-induced SERCA stimulation[4], compounds were also tested on SERCA1 in microsomes of guinea-pig skeletal muscle (PLN-free) before and after reconstitution with the PLN_{1-32} inhibitory fragment at a ratio of 300:1 for PLN:SERCA.

Left ventricles (LV) were dissected from rat and guinea-pig of healthy and failing preparations and frozen until use. Tissues were homogenized, subjected to centrifugation to obtain SR-enriched microsomes and sarcomeric proteins were extracted, as previously described[4].

In the case of guinea-pig hearts, LV tissues were homogenized in 4 volumes of 10 mM $NaHCO_3$, 1 mM PMSF, 10 μg/ml aprotinin and leupeptin (pH 7) and centrifuged at 12,000g for 15 minutes. Supernatants were filtered and centrifuged at 100,000 g for 30 min. Contractile proteins were extracted by suspending the pellets with 0.6 M KCl, 30 mM histidine, pH 7 and further centrifugation at 100,000 g for 30 min. Final pellets were reconstituted with 0.3 M sucrose, 30 mM histidine, pH 7, to obtain SR-enriched microsomes.

In the case of rat hearts, cardiac homogenates were used to have sufficient material to replicate the experiments within a single animal. Hearts were excised from rats eight weeks after STZ injection. LV tissues from healthy and STZ rats were homogenized (1 g in 4 ml buffer) in a medium containing 300 mM sucrose, 50 mM K-phosphate, 10 mM NaF, 0.3 mM PMSF, 0.5 mM DTT, pH 7, and centrifuged at 35,000 g for 30 min. The final pellet was resuspended in the same buffer.

SERCA2a ATPase activity was measured as the rate of ^{32}P-ATP release at multiple Ca^{2+} concentrations (100-2000 nM) in the absence and presence of tested compounds, as previously described[5]. Increasing concentrations of each compound were pre-incubated with 2 μg of cardiac preparations for 5 min at 4° C in 80 μl of a solution containing 100 mM KCl, 5 mM $MgCl_2$, 1 μM A23187, 20 mM Tris, pH 7.5. Then, 20 μl of 5 mM Tris-ATP containing 50 nCi of ^{32}P-ATP (3-10 Ci/mmol, Perkin Elmer) were added. The ATP hydrolysis was continued for 15 min at 37°C and the reaction was stopped by acidification with 100 μl of 20% ice-cold perchloric acid. ^{32}P was separated by centrifugation with activated charcoal (Norit A, Serva) and the radioactivity was measured. SERCA2a-dependent ATPase activity was identified as the portion of total hydrolytic activity inhibited by cyclopiazonic acid (CPA, 10 μM). Technical replicates were used to ensure the reliability of single values.

Reconstitution of SERCA1 with PLN_{1-32} synthetic fragment

Adult healthy male guinea-pigs were used to prepare SERCA1-enriched SR microsomes from fast-twitch hind leg muscles. Microsomes were prepared as described for SERCA2a preparations. For reconstitution experiments, SERCA1 (PLN-free) was pre-incubated with synthetic PLN_{1-32} fragment (canine sequence, Biomatik Corporation, Canada) in 20 mM imidazole, pH 7, at 1:300 SERCA1:PLN ratio for 30 min at room temperature. After pre-incubation, SERCA1 (PLN-free) alone, or reconstituted with PLN_{1-32}

fragment, was utilized for SERCA activity measurement by using ^{32}P-ATP hydrolysis method at different Ca^{2+} concentrations (25-2000 nM) in the absence and presence of increasing concentrations of tested compounds, as described for SERCA2a ATPase activity.

Ca^{2+} dose-response curves of SERCA ATPase activity were fitted by nonlinear regression (KaleidagraphTM, Sinergy Software); the parameters maximal hydrolytic velocity ($V_{max,}$ μmol/min/mg protein) and Ca^{2+} dissociation constant (KdCa, nM) were estimated. Either an increase of V_{max}, or a decrease of KdCa (increased Ca^{2+} affinity), stands for enhancement of SERCA function.

Functional measurements in isolated myocytes

Rat LV myocytes were isolated by using a retrograde coronary perfusion method previously published[6] with minor modifications. Rod-shaped, Ca^{2+}-tolerant myocytes were used within 12 h from dissociation. LV myocytes were clamped in the whole-cell configuration (Axopatch 200A, Axon Instru-ments Inc., Union City, CA). During measurements, myocytes were superfused at 2 ml/min with Tyrode's solution containing 154 mM NaCl, 4 mM KCl, 2 mM $CaCl_2$, 1 mM $MgCl_2$, 5 mM HEPES/NaOH, and 5.5 mM D-glucose, adjusted to pH 7.35. A thermo-stated manifold, allowing for fast (electronically timed) solution switch, was used for cell superfusion. All measurements were performed at 35 °C. Standard pipette solution contained 110 mM K^+ -aspartate, 23 mM KCl, 0.2 mM $CaCl_2$ (10^{-7} M calculated free-Ca^{2+} concentration), 3 mM $MgCl_2$, 5 mM HEPES-KOH, 0.5 mM EGTA-KOH, 0.4 mM GTP-Na^+ salt, 5 mM ATP-Na^+ salt, and 5 mM creatine phosphate Na^+ salt, pH 7.3. Membrane capacitance and series resistance were measured in every cell but left un-compensated. Current signals were filtered at 2 KHz and digitized at 5 KHz (Axon Digidata 1200). Trace acquisition and analysis was controlled by dedicated software (Axon pClamp 8.0).

Na^+/K^+ ATPase current (I_{NaK}) measurements

I_{NaK} was recorded in isolated LV myocytes from healthy rats as the holding current recorded at -40 mV in the presence of Ni^{2+} (5 mM), nifedipine (5 μM), Ba^{2+} (1 mM) and 4-aminopyridine (2 mM) to minimize contamination by changes in Na^+/Ca^{2+} exchanger (NCX), Ca^{2+} and K^+ currents, respectively. Tetra-ethylammonium-Cl (20 mM) and EGTA (5 mM) were added to the pipette solution and intracellular K^+ was replaced by Cs^+. To optimize the recording conditions, I_{NaK} was enhanced by increasing intracellular Na^+ (10 mM) and extracellular K^+ (5.4 mM)[7]. Compound 5 was dissolved in dimethyl sulfoxide (DMSO). Control and test solutions con-tained maximum 1:100 DMSO.

Intracellular Ca^{2+} dynamics

LV myocytes were incubated in Tyrode's solution for 30 min with the membrane-permeant form of the dye, Fluo4-AM (10 μM), and then washed for 15 min to allow dye de-esterification. Fluo4 emission was collected through a 535 nm band pass filter, converted to voltage, low-pass filtered (100 Hz) and digitized at 2 kHz after further low-pass digital filtering (FFT, 50 Hz). After subtraction of background

luminescence, a reference fluorescence (F_0) value was used for signal normalization (F/F_0). Cytosolic Ca^{2+} activity was dynamically measured in patch-clamped LV myocytes from STZ rats; membrane current, whose time-dependent component mainly reflected the sarcolemmal Ca^{2+} current (I_{CaL}), was simultaneously recorded with Fluo4 fluorescence. Drug effects on SR Ca^{2+} uptake rate were evaluated with a "SR loading protocol" specifically devised to rule out the contribution of NCX and to assess the SR Ca^{2+} uptake rate at multiple levels of SR Ca^{2+} loading[8,9] (protocol below in Figure S2). The protocol consisted in emptying the SR by a brief caffeine (10 mM) pulse and then progressively refilling it by voltage steps (-35 to 0 mV) activating Ca^{2+} influx through I_{CaL}. NCX was blocked by omission of Na^+ from intracellular and extracellular (replaced by equimolar Li^+ and 1 mM EGTA) solutions. The procedure is in agreement with published methods, with minor modifications[8,9]. Multiple parameters, suitable to quantify SR Ca^{2+} uptake, can be extracted from Ca^{2+} and I_{CaL} response to the protocol: the time constant (τ) of cytosolic Ca^{2+} decay within each V-step largely reflects net Ca^{2+} flux across the SR membrane (the faster SR Ca^{2+} uptake, the smaller τ decay). Because of the steep dependency of Ca^{2+} transient (Ca_T) amplitude on SR Ca^{2+} content, the rate at which Ca_T amplitude increases across the subsequent pulses of the protocol reflects the rate at which the SR refills. To rule out the potential contribution of changes in I_{CaL}, in each loading step, Ca_T amplitude was normalized to Ca^{2+} influx (estimated from I_{CaL} integral up to Ca_T peak) to obtain excitation-release (ER) gain. As expected from its strong dependency on SR Ca^{2+} content, this parameter progressively increases during the loading protocol. Diastolic Ca^{2+} of the first step was used as F_0 for signal normalization (F/F_0). Specificity of the "loading protocol" parameters in detecting SERCA2a activation is supported by the observation that they did not detect any effect of digoxin, an inotropic agent blocking the Na^+/K^+ pump and devoid of SERCA2a stimulating effect[10].

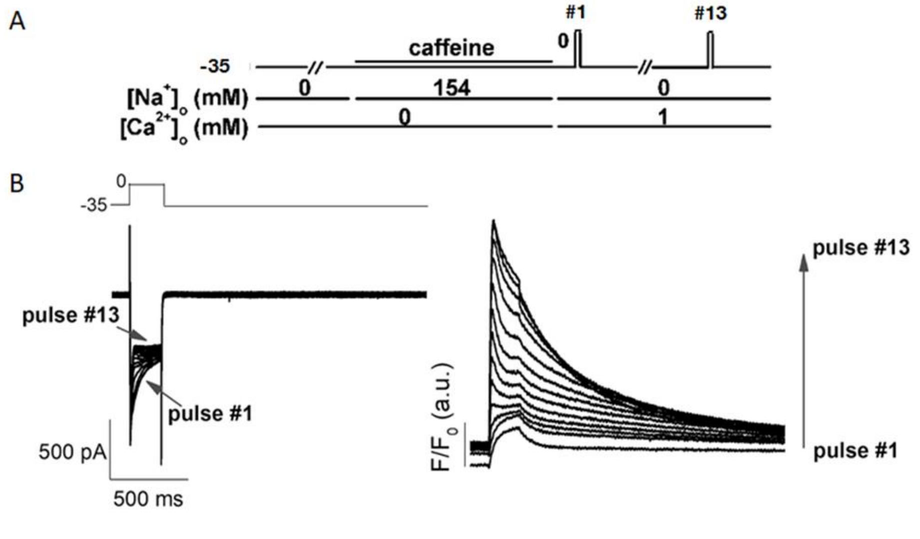

Figure S2. Protocol to evaluate SR Ca^{2+} uptake function under Na^+ free condition (NCX inhibition). **A)** Protocol outline. **B)** Transmembrane current (left) and Ca^{2+} transients (right) recordings during SR reloading after caffeine-induced SR depletion in patch-clamped cells.

***In-vivo* studies**

Drug toxicity studies in mice

Acute toxicity was determined in CD1 mice. Separate mice groups were either orally treated, or intravenously injected, with increasing doses of the tested substance to identify the dose causing 50% mortality (LD_{50}). Acute mortality was assessed at 30 min after compound administration; survival was assessed again at 24h.

Hemodynamic studies in rats with diabetic cardiomyopathy

Diabetes was selected as the *in-vivo* model because of its association with reduced SERCA2a function[9,11,12]. Diabetes was induced in male SD rats by a single injection into the tail vein of a solution of STZ (50 mg/kg, Sigma-Aldrich, dissolved in citrate buffer). Control (healthy group) rats received STZ vehicle (citrate buffer). Fasting glycaemia was measured after 1 week and rats with values >290 mg/dL were considered diabetic[9].

Eight weeks after STZ injection, rats were submitted to transthoracic echocardiographic evaluations, performed under 1.25 g/kg urethane i.p. anesthesia. Two-dimensionally guided M-mode recordings were used to obtain short-axis measurements of left ventricular (LV) end-diastolic diameter (LVEDD), LV end-systolic diameter (LVESD), posterior (PW) and interventricular septal (IVS) wall thickness according to the American Society of Echocardiography guidelines[13]. Fractional shortening was calculated as FS = (LVEDD-LVESD)/LVEDD. Trans-mitral flow velocity was measured by pulsed Doppler at the tips of mitral leaflets from an apical 4-chamber view to obtain early and late filling velocities (E, A waves) and E wave deceleration time (DT). The mitral deceleration time was also normalized to E wave amplitude (DT/E ratio). Mitral annular movements, i.e. peak myocardial systolic (s') and diastolic velocities (e' and a'), were measured by Tissue Doppler Imaging (TDI) in the apical 4-chamber view. Two-dimensional LV mass and its relative index to body weight, were estimated in healthy and STZ rats. All measurements were made by a blinded investigator, using an average of 3 consecutive cardiac cycles.

To evaluate the effect of i.v. infusion with compound **5**, a polyethylene 50 cannula was inserted into a jugular vein for drug infusion in rats. Rats were randomly submitted to an echocardiographic study performed before and during i.v. infusion of 0.2 mg/kg/min (0.16 ml/min) under urethane anesthesia. The echocardiography started 13 min and 27 min after the beginning of infusion, when a total cumulative dose of 2.6 and 5.4 mg/kg was infused. The echocardiography lasted on average 3.5 min, therefore at the last interrogation (always TDI), a total dose of approximately 6 mg/kg was infused.

At the end of the echocardiography, animals were sacrificed by i.v. administration of KCl.

Supplementary tables

Table S1. Effect of compounds **5** and **8** on SERCA2a activity in cardiac SR homogenates from **healthy rats.** Data are expressed as mean ± SEM, N = number of experiments.

**SERCA2a**						
			**KdCa (nM)**		**V_{max} (µmol/min/mg)**	
**Cpd**	**Concentration nM**	**N**	**control**	**Cpd**	**control**	**Cpd**
**5**	100	6	224 ± 25	221 ± 24	0,379 ± 0,023	0,371 ± 0,021
	300			221 ± 21		0,384 ± 0,023
	500	6	208 ± 30	220 ± 24	0,370 ± 0,023	0,374 ± 0,031
	1000			219 ± 32		0,377 ± 0,029
**8**	100	5	231 ± 18	214 ± 17	0,364 ± 0,025	0,344 ± 0,038
	300			210 ± 13		0,366 ± 0,027
	500	5	282 ± 29	260 ± 35	0,314 ± 0,021	0,313 ± 0,028
	1000			273 ± 32		0,320 ± 0,020

Table S2. Effect of compound **5** on echocardiographic and tissue Doppler parameters in STZ rats after intravenous infusion at the dose of 0.2 mg/kg/min. Echo parameters were measured at 15 and 30 min after infusion start and 10 min after drug interruption under urethane anesthesia. Data are mean ± SEM. *p<0.05 vs basal (one-way ANOVA for repeated measurements).

		STZ			
		**basal**	**after 15 min Cpd 5**	**after 30 min Cpd 5**	**after 10 min stop**
**Morphometric parameters**	**IVSTd, mm**	1,68±0,09	1,81±0,08	1,71±0,1	1,68 ± 0,07
	**PWTd, mm**	1,22±0,07	1,16±0,07	1,22±0,07	1,17 ± 0,07
	**LVEDD, mm**	6,73±0,15	6,86±0,17	6,94±0,15	6,84 ± 0,17
	**IVSTs, mm**	2,31±0,14	2,34±0,10	2,55±0,13	2,55 ± 0,10
	**PWTs, mm**	2,5±0,09	2,59±0,09	2,53±0,10	2,42 ± 0,10
	**LVESD, mm**	2,84±0,17	2,87±0,18	2,73±0,18	2,64 ± 0,17
**Systolic function**	**FS, %**	57,9±2,01	58,1±2,38	60,7±2,31	61,3±2,05
	**s', mm/s**	21,3±0,59	22,4±0,86	21,8±0,78	20,6±0,53
	**EF, %**	90,85±1,25	90,68±1,4	92,19±1,18	92,66±1,05
**Diastolic function**	**E, mm/s**	0,78±0,031	0,88±0,04*	0,91±0,05*	0,78±0,03
	**A, mm/s**	0,55±0,034	0,65±0,04*	0,69±0,032*	0,58±0,02
	**E/A**	1,46±0,10	1,36±0,05	1,34±0,06	1,37±0,06
	**DT, ms**	53,8±2,72	42,46±2,83*	42,15±2,74*	52,4±4,36
	**DT/E, s2/mm**	71,4±5,31	49,52±3,44*	48,51±4,56*	68,2±6,13
	**E/DT, m/s2**	15,14±1,4	21,49±1,61*	23,18±2,32*	16,5±1,78
	**e', mm/s**	20,38±0,64	23,33±0,73*	24,24±0,65*	19,4±0,60
	**a', mm/s**	22,99±1,57	29,13±1,82*	28,94±1,47*	26,22±1,31*
	**e'/a'**	0,95±0,09	0,84±0,07	0,86±0,05	0,76±0,047*
	**E/e'**	38,03±0,99	37,49±1,2	37,45±1,24	40,22±0,82
**Overall cardiac function**	**HR, bpm**	241±13	268±15	252±14	228±10
	**SV, ml**	0,64±0,04	0,68±0,05	0,71±0,04	0,68±0,05
	**CO, ml/min**	151,5±8,13	177,2±11,27*	175,15±9,4*	153,2±10,67
	**N**	13			

References

(1) De Munari, S.; Cerri, A.; Gobbini, M.; Almirante, N.; Banfi, L.; Carzana, G.; Ferrari, P.; Marazzi, G.; Micheletti, R.; Schiavone, A.; Sputore, S.; Torri, M.; Zappavigna, M. P.; Melloni, P. Structure-Based Design and Synthesis of Novel Potent Na+,K +-ATPase Inhibitors Derived from a 5α,14α-Androstane Scaffold as Positive Inotropic Compounds. *J. Med. Chem.* **2003**, *46* (17), 3644–3654. https://doi.org/10.1021/jm030830y.

(2) Jørgensen, P. L. Purification of Na+,K+-ATPase: Enzyme Sources, Preparative Problems, And Preparation from Mammalian Kidney. *Methods Enzymol.* **1988**, *156*, 29–43. https://doi.org/10.1016/0076-6879(88)56005-6.

(3) Ferrandi, M.; Tripodi, G.; Salardi, S.; Florio, M.; Modica, R.; Barassi, P.; Parenti, P.; Shainskaya, A.; Karlish, S.; Bianchi, G.; Ferrari, P. Renal Na,K-ATPase in Genetic Hypertension. *Hypertension* **1996**, *28*, 1018–1025. https://doi.org/10.1161/01.HYP.28.6.1018.

(4) Ferrandi, M.; Barassi, P.; Tadini-Buoninsegni, F.; Bartolommei, G.; Molinari, I.; Tripodi, M. G.; Reina, C.; Moncelli, M. R.; Bianchi, G.; Ferrari, P. Istaroxime Stimulates SERCA2a and Accelerates Calcium Cycling in Heart Failure by Relieving Phospholamban Inhibition. *Br. J. Pharmacol.* **2013**, *169*, 1849–1861. https://doi.org/10.1111/bph.12278.

(5) Micheletti, R.; Palazzo, F.; Barassi, P.; Giacalone, G.; Ferrandi, M.; Schiavone, A.; Moro, B.; Parodi, O.; Ferrari, P.; Bianchi, G. Istaroxime, a Stimulator of Sarcoplasmic Reticulum Calcium Adenosine Triphosphatase Isoform 2a Activity, as a Novel Therapeutic Approach to Heart Failure. *Am. J. Cardiol.* **2007**, *99*, 24A-32A. https://doi.org/10.1016/j.amjcard.2006.09.003.

(6) Rocchetti, M.; Sala, L.; Rizzetto, R.; Irene Staszewsky, L.; Alemanni, M.; Zambelli, V.; Russo, I.; Barile, L.; Cornaghi, L.; Altomare, C.; Ronchi, C.; Mostacciuolo, G.; Lucchetti, J.; Gobbi, M.; Latini, R.; Zaza, A. Ranolazine Prevents INaL Enhancement and Blunts Myocardial Remodelling in a Model of Pulmonary Hypertension. *Cardiovasc. Res.* **2014**, *104*, 37–48. https://doi.org/10.1093/cvr/cvu188.

(7) Rocchetti, M.; Besana, A.; Mostacciuolo, G.; Ferrari, P.; Micheletti, R.; Zaza, A. Diverse Toxicity Associated with Cardiac Na+/K+ Pump Inhibition: Evaluation of Electrophysiological Mechanisms. *J. Pharmacol. Exp. Ther.* **2003**, *305*, 765–771. https://doi.org/10.1124/jpet.102.047696.

(8) Arici, M.; Ferrandi, M.; Barassi, P.; Hsu, S.-C.; Torre, E.; Luraghi, A.; Ronchi, C.; Chang, G.-J.; Peri, F.; Ferrari, P.; Bianchi, G.; Rocchetti, M.; Zaza, A. Istaroxime Metabolite PST3093 Selectively Stimulates SERCA2a and Reverses Disease-Induced Changes in Cardiac Function Corresponding Authors. *BioRxiv* **2022**. https://doi.org/10.1101/2021.08.17.455204.

(9) Torre, E.; Arici, M.; Lodrini, A. M.; Ferrandi, M.; Barassi, P.; Hsu, S.-C.; Chang, G.-J.; Boz, E.; Sala, E.; Vagni, S.; Altomare, C.; Mostacciuolo, G.; Bussadori, C.; Ferrari, P.; Bianchi, G.; Rocchetti, M. SERCA2a Stimulation by Istaroxime Improves Intracellular Ca2+ Handling and Diastolic Dysfunction in a Model of Diabetic Cardiomyopathy. *Cardiovasc. Res.* **2021**. https://doi.org/10.1093/cvr/cvab123.

(10) Rocchetti, M.; Besana, A.; Mostacciuolo, G.; Micheletti, R.; Ferrari, P.; Sarkozi, S.; Szegedi, C.; Jona, I.; Zaza, A. Modulation of Sarcoplasmic Reticulum Function by Na+/K + Pump Inhibitors with Different Toxicity: Digoxin and PST2744 [(E,Z)-3-((2-Aminoethoxy)Imino)Androstane-6,17-Dione Hydrochloride]. *J. Pharmacol. Exp. Ther.* **2005**, *313*, 207–215. https://doi.org/10.1124/jpet.104.077933.

(11) Choi, K. M.; Zhong, Y.; Hoit, B. D.; Grupp, I. L.; Hahn, H.; Dilly, K. W.; Guatimosim, S.; Jonathan Lederer, W.; Matlib, M. A. Defective Intracellular Ca2+ Signaling Contributes to Cardiomyopathy in Type 1 Diabetic Rats. *Am. J. Physiol. - Hear. Circ. Physiol.* **2002**, *283*, H1398–H1408. https://doi.org/10.1152/ajpheart.00313.2002.

(12) Vasanji, Z.; Dhalla, N. S.; Netticadan, T. Increased Inhibition of SERCA2 by Phospholamban in the Type I Diabetic Heart. *Mol. Cell. Biochem.* **2004**, *261*, 245–249. https://doi.org/10.1023/B:MCBI.0000028762.97754.26.

(13) Lang, R. M.; Bierig, M.; Devereux, R. B.; Flachskampf, F. A.; Foster, E.; Pellikka, P. A.; Picard, M. H.; Roman, M. J.; Seward, J.; Shanewise, J.; Solomon, S.; Spencer, K. T.; St John Sutton, M.;

Stewart, W. American Society of Echocardiography's Nomenclature and Standards Committee; Task Force on Chamber Quantification; American College of Cardiology Echocardiography Committee; American Heart Association; European Association of Echocardiography; European So. *Eur J Echocardiogr* **2006**, *7*, 79–108.
